# Differential gene expression analysis tools exhibit substandard performance for long non-coding RNA-sequencing data

**DOI:** 10.1186/s13059-018-1466-5

**Published:** 2018-07-24

**Authors:** Alemu Takele Assefa, Katrijn De Paepe, Celine Everaert, Pieter Mestdagh, Olivier Thas, Jo Vandesompele

**Affiliations:** 1 Department of Data Analysis and Mathematical Modeling, Ghent University, Ghent, Belgium; 2 National Institute for Applied Statistics Research, University of Wollongong, Wollongong, Australia; 3 Department of Pediatrics and Medical Genetics, Ghent University, Ghent, Belgium; 4 Global Advanced Analytics Group, Bain & Company Belgium, Inc., Brussels, Belgium

**Keywords:** RNA-seq, mRNA, lncRNA, Differential gene expression

## Abstract

**Background:**

Long non-coding RNAs (lncRNAs) are typically expressed at low levels and are inherently highly variable. This is a fundamental challenge for differential expression (DE) analysis. In this study, the performance of 25 pipelines for testing DE in RNA-seq data is comprehensively evaluated, with a particular focus on lncRNAs and low-abundance mRNAs. Fifteen performance metrics are used to evaluate DE tools and normalization methods using simulations and analyses of six diverse RNA-seq datasets.

**Results:**

Gene expression data are simulated using non-parametric procedures in such a way that realistic levels of expression and variability are preserved in the simulated data. Throughout the assessment, results for mRNA and lncRNA were tracked separately. All the pipelines exhibit inferior performance for lncRNAs compared to mRNAs across all simulated scenarios and benchmark RNA-seq datasets. The substandard performance of DE tools for lncRNAs applies also to low-abundance mRNAs. No single tool uniformly outperformed the others. Variability, number of samples, and fraction of DE genes markedly influenced DE tool performance.

**Conclusions:**

Overall, linear modeling with empirical Bayes moderation (limma) and a non-parametric approach (SAMSeq) showed good control of the false discovery rate and reasonable sensitivity. Of note, for achieving a sensitivity of at least 50%, more than 80 samples are required when studying expression levels in realistic settings such as in clinical cancer research. About half of the methods showed a substantial excess of false discoveries, making these methods unreliable for DE analysis and jeopardizing reproducible science. The detailed results of our study can be consulted through a user-friendly web application, giving guidance on selection of the optimal DE tool (http://statapps.ugent.be/tools/AppDGE/).

**Electronic supplementary material:**

The online version of this article (10.1186/s13059-018-1466-5) contains supplementary material, which is available to authorized users.

## Background

Messenger RNA (mRNA) has been the primary target of transcriptome studies. However, RNA sequencing technology has revealed that the human genome is pervasively transcribed, resulting in thousands of novel non-coding RNA genes. Hence, attention is expanding to one of the most poorly understood, yet most common RNA species: long non-coding RNAs (lncRNAs) [1, 2]. These lncRNAs form a large and diverse class of transcribed RNA molecules, constituting up to 70% of the transcriptome with a defined length of 200 nucleotides. While they do not encode proteins, lncRNAs are strong regulators of gene expression [3]. The discovery and study of lncRNAs are of major relevance to human health and disease because they represent an extensive, largely unexplored, and functional component of the genome [3–5]. In contrast to mRNAs, lncRNAs are generally expressed in low amounts, typically an order of magnitude lower than mRNA expression levels [2, 6, 7]. Furthermore, several studies [7–9] demonstrated that lncRNA expression levels are very noisy, which is a characteristic shared with low count data from massively parallel RNA sequencing.

Following the advent of RNA-sequencing (RNA-seq) technologies, several statistical tools for differential gene expression (DGE) analysis have been introduced. However, low and noisy read counts, such as those coming from lncRNAs, are potentially challenging for the tools [10, 11]. For example, it is commonly observed that low count genes show large variability of the fold-change estimates and thus exhibit inherently noisier inferential behavior. The majority of the methods suggest removal of low expressed genes before the start of data analysis, but this procedure essentially blocks researchers from studying lncRNAs. In our study, no such severe filtering was applied, leaving almost all lncRNAs in the dataset. To our knowledge, no statistical method has been specifically developed for the analysis of lncRNA-seq data and therefore transcriptome studies make use of statistical methods that assume sufficient expression levels. In this paper, we evaluated and compared the performance of many popular statistical methods (Table 1) developed for testing DGE of RNA-seq data (hereafter referred to as “DE tools”), with special emphasis on lncRNAs and low-abundance mRNAs. All tools considered in this study are popular (in terms of number of citations), available as R software packages [12], and use gene or transcript level read counts as input. Our conclusions are based on six RNA-seq datasets and many realistic simulations, representing various typical gene expression experiments.

**Table 1:** List of DE tools and pipelines along with their reference and number of citations

Tool (package version)	Pipelines	Reference	Citations^a^
edgeR (3.14.0)	(1) Exact test based on NB distribution, (2) GLM with NB family, (3) QL, (4–7) robust GLM with four different prior DF	[32]	5406
DESeq (1.24.0)	(1) Default, exact test based on NB distribution	[45]	4655
DESeq2(1.12.4)	Fits GLM with NB family. (1) Default, (2) independent filtering disabled (setting1), (3) independent filtering disabled and outlier-detection off (setting2)	[10]	1364
limma (3.25.21)	Fits linear models on log-transformed counts. (1) Voom, (2) voom (robust), (3) trended, (4) trended (robust), (5) voom+QW, (6) limmaVST, (7) limmaQN	[33]	1828
NOISeq (2.12.1)	(1) Default, data-adaptive and non-parametric method	[53]	524
baySeq (2.6.0)	(1) Default, Bayesian methods with empirical prior distributions	[52]	315
SAMSeq (samr, 2.0)	(1) Default, non-parametric method based on Wilcoxon rank sum statistic	[19]	140
PoissonSeq (1.1.2)	(1) Default, uses poisson log-linear model	[46]	92
QuasiSeq (1.0.8)	Fits GLM with NB family. (1) QL, (2) QLShrink, (3) QLSPline	[34]	57

^a^As reported by Web of Science (http://www.webofknowledge.com/; April 25, 2018). Further description about the pipelines can be found in the “Methods” section.

*Abbreviations*: *DF*=degrees of freedom, *GLM*=generalized linear models, *NB*=negative binomial, *QL*=quasi-likelihood, *QN*=quantile normalization, *QW*=quality weight, *VST*=variance stabilizing transformation

Previous comparative studies of DE tools [11, 13–18] focused on mRNA and some of these concluded that DE tools show inferior performance for genes or transcripts with low counts. We extend previous studies by including lncRNAs and low expressed mRNAs separately. Further, our results are based on diverse types of RNA-seq datasets that vary with respect to their biological and technical features, such as species (human, mouse, rat), experimental design (control versus treatment, diseased versus non-diseased, and tissue comparisons), and level of biological variability. We assess the degree of concordance among results returned from the DE tools, and we study important statistical properties of DE tools, such as their ability to control the false discovery rate (FDR) and their sensitivity for the detection of DE. The latter are empirically investigated using a non-parametric resampling-based simulation procedure. The simulation method essentially resamples data from a real RNA-seq dataset to create realistic gene expression scenarios. Consequently, our results reflect the genuine behaviour of the DE tools under study, in contrast to simulation studies based on parametric assumptions. Note that the evaluation of a method that relies on a parametric assumption (e.g., edgeR, DESeq, and DESeq2 assume a negative binomial distribution) by means of simulated counts using the same distribution as used in [14] will give too optimistic results. Moreover, these results do not reflect a realistic setting because the distributional assumption cannot be expected to hold in general [19]. By starting from a variety of real and representative RNA-seq datasets, the scope of our findings is wide. To our knowledge, our study is the largest empirical evaluation conducted so far in terms of the number of real datasets used, the number of performance metrics evaluated, and the number of DE pipelines included (Additional file 1: Figure S1).

**Fig. 1:**
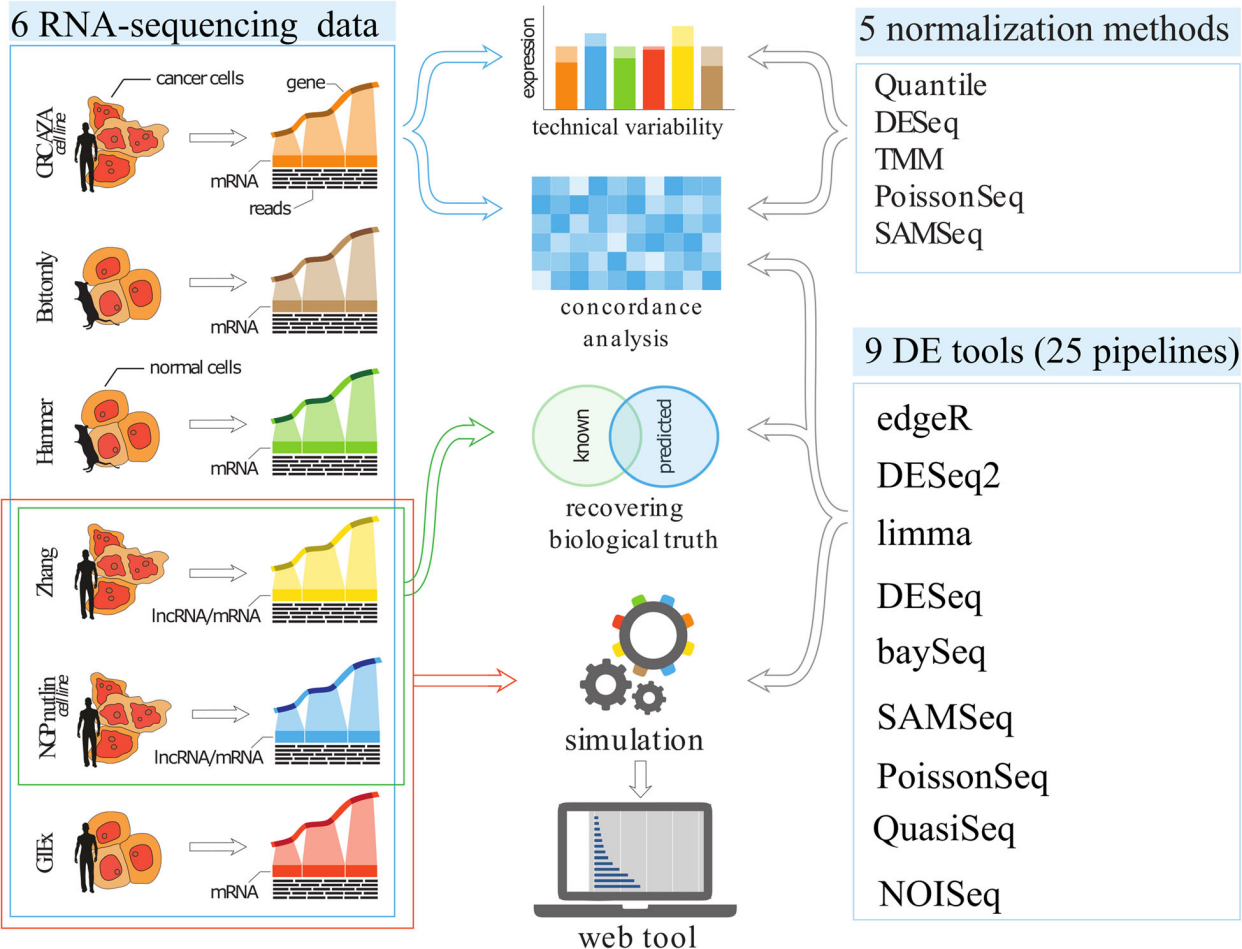
DE tools assessment work flow. The study has four components: evaluation of five normalization methods, concordance analysis of DE tools, evaluating the capability of DE tools to recover genes with known biological evidence of differential expression, and simulation procedures to study the statistical properties of DE tools, such as their ability to control the FDR and their sensitivity for the detection of differential expression. Six diverse types of RNA-seq datasets were used for comparison of the normalization methods and concordance analysis of DE tools. RNA-seq datasets were obtained from two cultured cell line datasets (CRC AZA and NGP nutlin), inbred animals (Bottomly and Hammer), normal human tissues (GTEx), and human cancer cells (Zhang). Three series of simulations were performed, each starting from a different RNA-seq source dataset: Zhang, NGP nutlin, and GTEx data. Results of the simulation study are made available through a user-friendly web application

Our study consists of four parts: first we evaluated various normalization procedures; second we compared the level of agreement among DE pipelines using various publicly available RNA-seq datasets; third we explored the ability of the DE pipelines to recover known evidence of differential expression; and fourth we used simulation procedures to evaluate and compare the performance of the tools under a variety of gene expression experiment scenarios (Fig. 1), such as variability, sample size, and fraction of DE genes.

## Results and discussion

### RNA-seq datasets

Six publicly available benchmark RNA-seq datasets were used for the concordance analysis. Three of them were used as source datasets for generating non-parametric simulated data. The description of the datasets can be found in the “Methods” section; a summary is presented in Table 2.

**Table 2:** Summary of datasets

					Library size (× 10^6^)				
Dataset and reference	Replicate size	Species	Type	Number of genes	Min.	Max.	Correlation among replicates^a^	Sequencing instrument	Library type
CRC AZA	{3, 3}	Human	mRNA	16,499	3.3	4.2	(0.99, 0.99)	HiSeq 2000	PE, 100, poly(A)
Hammer [39]	{2, 2}	Rat	mRNA	15,908	17.3	23.5	(0.99, 0.99)	GAII	SR, 50, poly(A)
Bottomly [40]	{10, 11}	Mouse	mRNA	12,784	2.7	7.3	(0.82, 0.99)	GAIIx	SR, 30, poly(A)
GTEx [41]	{28, 30}	Human	mRNA	18,632	10.2	76.8	(0.45, 0.99)	HiSeq 2000	PE, 76, poly(A)
Zhang [36]	{81, 91}	Human	mRNA	19,254	10.8	31.7	(0.29, 0.98)	HiSeq 2000	PE, 90, poly(A)
			lncRNA	10,051	0.3	1.0	(0.19, 0.97)		
NGP nutlin	{10, 10}	Human	mRNA	17,489	13.4	18.1	(0.98, 0.99)	NextSeq 500	PE, 75, poly(A)
			lncRNA	8929	0.2	0.4	(0.83, 0.99)		

^a^Pearson’s correlation is calculated among replicates within conditions based on read counts

Data include the species, gene biotype, number of genes (annotated genes with at least one count in each condition), number of replicates per condition, library size (minimum, maximum), Pearson correlation among replicates (minimum, maximum), sequencing instrument, library type (*SR*=single read, *PE*=paired-end read, read length (nucleotides), sequencing type poly(A)/total).

The degree of homogeneity among samples, as measured by Pearson’s correlation coefficient, was lowest for the Zhang dataset followed by GTEx (see also the estimated biological coefficients of variation in Additional file 1: Figure S2). As expected, the other datasets had replicates that are more homogeneous because they were obtained from inbred animals or cultured cell lines, in contrast to the GTEx or Zhang datasets containing tissues for different human individuals. For the Zhang and NGP nutlin datasets, lncRNAs showed relatively higher heterogeneity across samples than mRNAs. In addition, lncRNAs showed, on average, lower expression than mRNAs (Additional file 1: Figure S3).

### Comparison of normalization methods

Comparing DE tools requires careful attention to the normalization methods. Previous studies [13, 16, 20, 21] have pointed out that the normalization procedure can affect DE results. The aim of our study is not to perform a comprehensive comparison of all normalization methods. Instead, we compared five normalization methods that are used in conjunction with the DE methods evaluated in this study. This will allow us to better understand the general behavior of the DE tools as evaluated in the subsequent sections. The normalization methods were compared using the metrics from Dillies et al. [20], such as their capability to reduce technical variability and to eliminate bias due to library size differences, and their effect on DGE analysis.

**Fig. 2:**
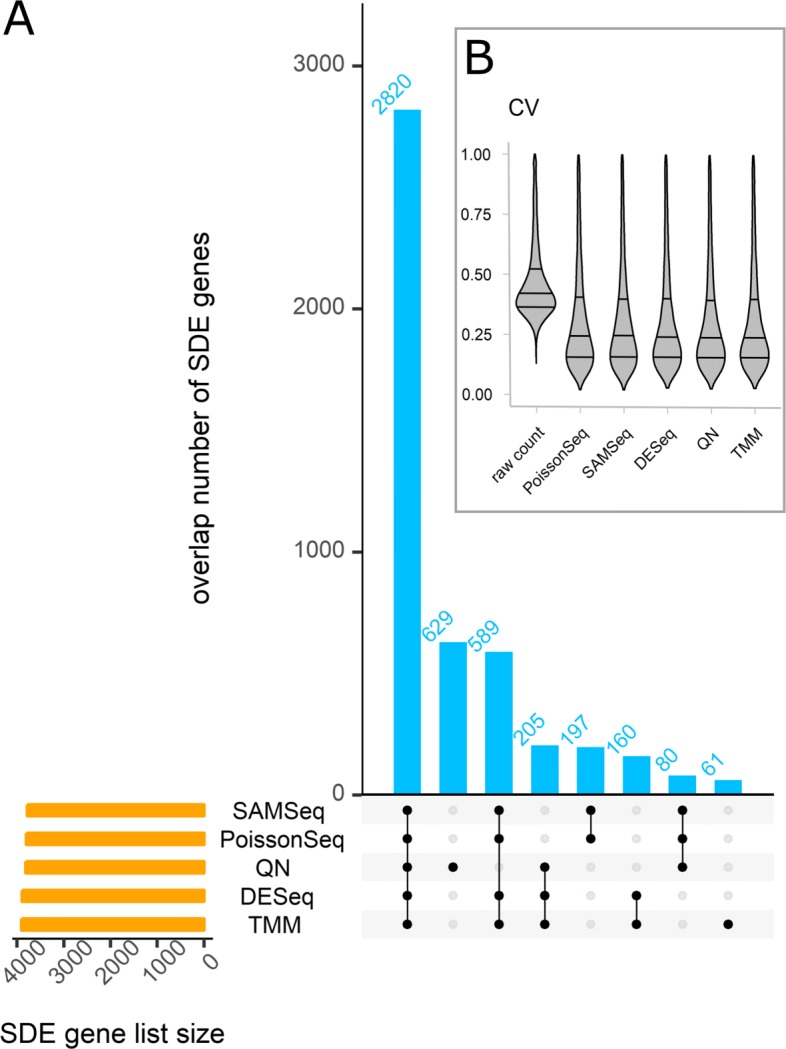
Effect of normalization methods on DGE analysis. **a** The UpSet diagram shows the intersection size among DGE analyses (at 5% FDR), each using different normalization methods but the same statistical test (moderated *t*-test from limma package). This result is particularly for the CRC AZA data. All considered normalization methods generally show strong concordance except for quantile normalization (*QN*). DGE analysis with all normalization methods commonly identified 2820 significantly differentially expressed (*SDE*) genes, whereas QN resulted in 629 SDE genes that are not shared with other normalization methods. **b** Distribution of gene-wise coefficients of variation (*CV*) from the Bottomly data. Each violin plot indicates the quartiles of the distribution (*solid horizontal lines*). Results based on all six datasets can be found in Additional file 2

Box plots of the relative log expressions show that for all six datasets all normalization methods succeed in aligning the sample-specific distributions and hence no library size effects were noticeable after normalization (Additional file 2: Section 2.2). Furthermore, the condition-specific gene-wise coefficient of variation (CV), which is a proxy for intra-group variability, was lower for all datasets upon normalization (Fig. 2b and Additional file 2: Section 2.3). Nearly equal levels of biological variability across methods were observed, even with quantile normalization that was found to result in high CV in other studies [20, 22]. The overlap of DE genes with different normalization methods was high (Fig. 2a and Additional file 2: Section 2.4). Ignoring quantile normalization (QN), on average (across the six dataset) a minimum of 86% similarity was observed. QN-based DE analysis gives deviating results, particularly for designs with small numbers of replicates (< 5); the average minimum proportion of similarity was 70.1% (average minimums are calculated across datasets). Overall, the results suggest that all normalization methods perform almost equally, except QN. Nevertheless, for the concordance analysis of the DE tools (see next section) we include a limma pipeline that uses QN (named limmaQN) to further investigate its effect on other performance metrics of DE tools.

### Concordance analysis

Twenty-five DE pipelines were run on six RNA-seq datasets, and (dis)similarities among the results were examined. The concordance analysis focused on five quantitative and one qualitative metric: (1) number of genes identified as significantly differentially expressed (SDE); (2) similarity in terms of the set of SDE genes; (3) the degree of agreement on gene ranking; (4) similarity of fold-change estimates; (5) handling of genes with special characteristics (lncRNAs, genes with low counts, genes with outliers); and (6) computation time. The results for individual datasets are presented in Additional file 3.

**Fig. 3:**
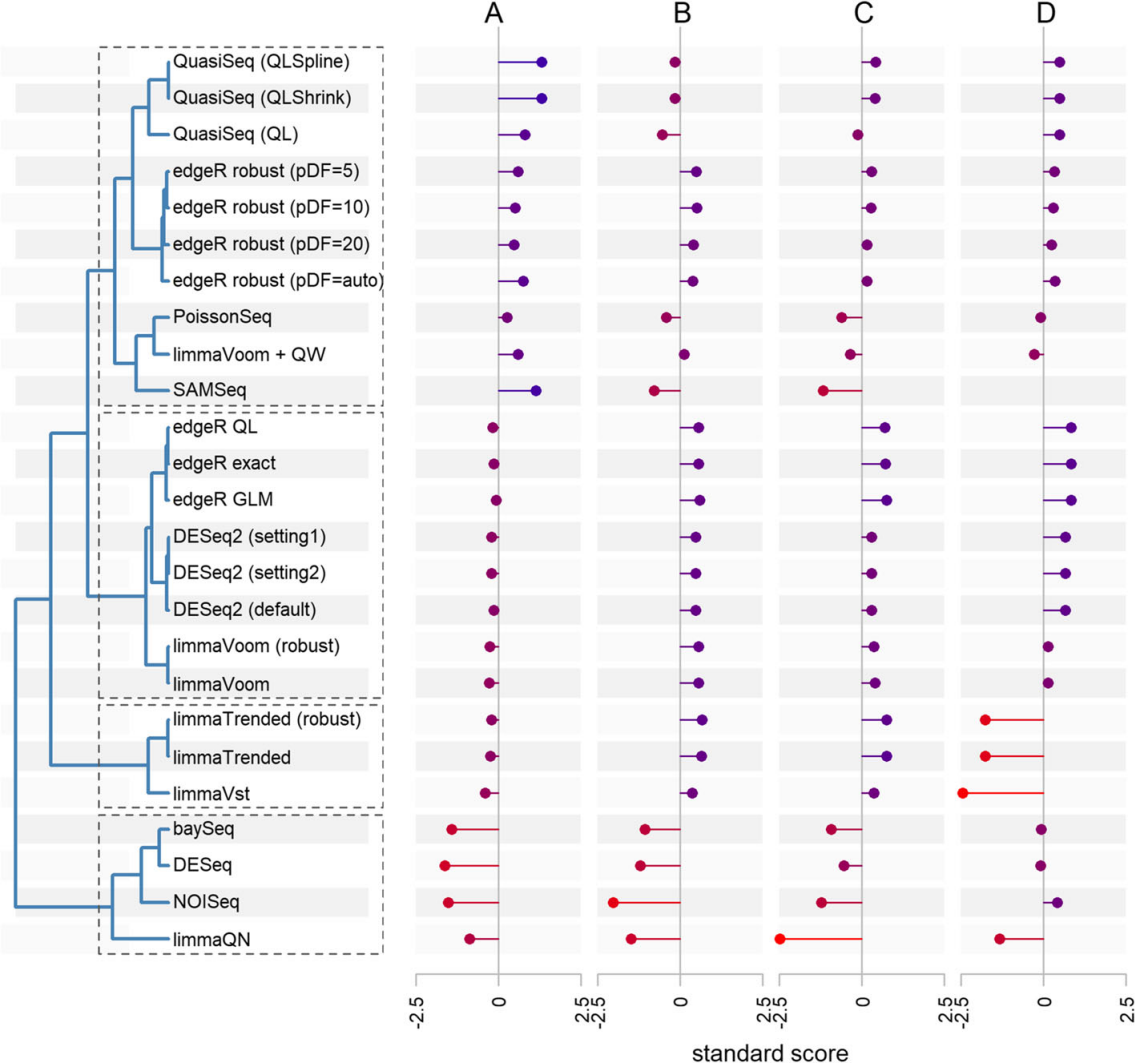
Summary of concordance analysis results. Hierarchical clustering of 25 DE pipelines based on standard scores of four concordance metrics (**a** fraction of significantly differentially expressed (SDE) genes detected at 5% FDR, **b** overlap among pipelines in detecting SDE genes at 5% FDR, **c** gene ranking agreement, and **d** similarity of log fold-change (LFC) estimates). Scores are averaged across the six datasets. First, observed values (let *y*_*i*_, i = 1,2,..., 25) of concordance metrics (proportions and correlations) for each pipeline from a given dataset are converted to standard scores (*z*_*i*_ = (*y*_*i*_ − *ȳ*)/*s*_*y*_, where *ȳ* and *s*_*y*_ are the mean and standard deviation of *y*_*i*_, respectively). Afterwards, the average of standard scores of each pipeline across datasets are presented. A negative value, for example, for the fraction of SDE genes indicates that the number of SDE genes detected by the pipeline is lower than the average across all the 25 pipelines. Subsequently, the Euclidean distance among the marginal standardized scores of the four comparison metrics are computed and the complete linkage method of agglomerative clustering is applied, resulting in four clusters. The bar plots to the right side of the cluster show the individual marginal scores of each DE tool for the four concordance measures. Since the fold-change estimate from SAMSeq is in terms of rank-sum, it was excluded from comparison of LFC estimates

Results show that the pipelines show substantial variability in the numbers of SDE genes. The marginal summary across all datasets indicates that DESeq, NOISeq, baySeq, and limmaQN detected the smallest number of SDE genes, whereas QuasiSeq and SAMSeq returned the largest numbers (Fig. 3). The variability among DE pipelines with respect to the number of SDE genes seems to be related to the biological variability in the dataset. For the Zhang and GTEx RNA-seq datasets, characterized by the largest intra-group biological variability, the numbers of SDE genes were quite different among the DE pipelines. In contrast, the numbers of SDE genes from the NGP nutlin and CRC AZA datasets, all displaying low biological variability, were relatively similar among pipelines. lncRNAs and low-abundance genes in general were under-represented among the SDE genes (Additional file 3). For example, 25% of the SDE genes were lncRNAs, whereas the data contain 40% lncRNAs.

Many of the DE pipelines showed agreement to each other in terms of the set of SDE genes (Fig. 3). On average, NOISeq, limmaQN, DESeq, baySeq, and SAMSeq showed the smallest concordance with all other tested pipelines. It was also observed that the overlap of SDE is smaller for lncRNAs than for mRNAs (Additional file 1: Figure S4). In the Zhang dataset, there is less than 70 and 60% SDE overlap across all DE tools for mRNAs and lncRNAs, respectively.

Accurate gene ranking is an essential step for downstream analysis such as gene set enrichment analysis (GSEA) [23]. The degree of agreement among the 25 DE pipelines’ gene ranking is studied using the rank of *π* scores; taking into account both the significance and magnitude of differential expression [24]. Summarized results across datasets (Fig. 3) indicate that all pipelines strongly agree, except for baySeq, NOISeq, SAMSeq, and limmaQN. Apart from baySeq, this is somewhat in contrast to the findings in Soneson and Delorenzi [14]. This might be due to the difference in the score used to rank genes, as only *p* values were used to rank genes in Soneson and Delorenzi [14]. Except for limmaQN, gene ranking agreement among all pipelines was nearly the same for lncRNAs and mRNAs from analyzing the NGP nutlin data. A slightly lower agreement for lncRNAs was observed when the most variable dataset (Zhang) was used (Additional file 1: Figure S4).

Moreover, the log fold-change (LFC) estimates from all DE tools were strongly correlated, with a minimum of 0.8 Pearson correlation coefficient (on average) for limmaVst, limmaQN, and limmaTrended pipelines (Fig. 3 and Additional file 3). However, the correlations become relatively stronger for the datasets with higher numbers of samples per group. In addition, the correlations for lncRNAs were lower than for mRNAs (Additional file 1: Figure S4 and Additional file 3: Sections 5.4 and 6.4).

In addition, we qualitatively examined the handling of genes with outlier expression (Additional file 1: Section 3.1). A set of genes with outlier count in only one of the samples (from the Zhang data) was chosen (Additional file 1: Figure S5). The adjusted *p* values for these outlier genes shows that edgeR exact, edgeR GLM, edgeR QL, PoissonSeq, QuasiSeq (both settings), and baySeq declared most of them SDE at 5% nominal FDR (Additional file 1: Table S2), suggesting that they can be affected by outlier expression.

To come to an overall conclusion, the results were combined in a hierarchical clustering analysis of the DE pipelines, resulting in 4 clusters (Fig. 3). DESeq, baySeq, limmaQN, and NOISeq cluster together, generally showing the lowest number of SDE genes, lower overlap, and lower gene ranking agreement with all other DE pipelines. The second cluster includes edgeR exact, edgeR GLM, edgeR QL, DESeq2 (both settings), and limmaVoom (robust and not robust), showing the highest concordance with respect to calling SDE, gene ranking, and LFC estimates. Pipelines in this cluster generally identify more SDE genes than methods in the first cluster. LimmaTrended (robust and not robust) and limmaVst appear in a separate cluster because of their relatively weakly correlated LFC estimates with that of other pipelines, but these pipelines strongly resemble the second cluster with respect to the other concordance metrics. The last cluster includes QuasiSeq (both settings), edgeR robust (with both tested prior degrees of freedom), limmaVoom+QW, PoissonSeq, and SAMSeq. They detect the most SDE genes and show a modest proportion of overlap, gene ranking agreement, and LFC similarity.

Moreover, with respect to identifying DE genes among genes that are detected only in one group of samples, DESeq, baySeq, and PoissonSeq fail to estimate a meaningful fold change. On the other hand, edgeR exact test, DESeq, and SAMSeq return no *p* value for such genes with a low signal-to-noise (STN) ratio (Additional file 1: Section 3.2). STN is defined as the ratio of the mean to the standard deviation of normalized counts in the group with detected gene expression [13]. In general, and not unexpectedly, all the pipelines assign significant *p* values for such genes with a high STN ratio (Additional file 1: Figure S6). This suggests that researchers need to be cautious when interpreting the DE results, particularly when the 0 read counts in one of the groups is likely caused by technical artefacts. Moreover, for lncRNAs (also for low-abundance mRNAs), the STN ratio is typically low, and hence all the DE pipelines fail to detect true DE among such genes. However, from the relationship between the STN and adjusted *p* values, one can learn that limma and QuasiSeq tools (and edgeR robust and DESeq2 to a lesser extent) detect such genes as SDE even at low STN (Additional file 1: Figure S6).

Results obtained with the three settings of DESeq2 were not markedly different, except that the independent filtering excluded more lncRNAs (29% from the Zhang data) than mRNAs (Additional file 1: Figure S7). Among the seven limma pipelines, voom and trended (with and without robust estimate of the prior degrees of freedom) showed relatively better concordance. In addition, voom with sample quality weight (limmaVoom+QW) tend to identify more SDE genes. Similarly, edgeR pipelines attained similar concordance except that edgeR robust detects slightly more SDE genes than the average. Although the three QuasiSeq pipelines cluster together, the quasi-likelihood (QL) method with an independent estimate of the gene-wise QL dispersion showed worse agreement in terms of the set of SDE genes.

The computation time to run DGE analysis presented in Additional file 1: Figure S8 shows that baySeq and DESeq require the longest time, whereas limma tools and PoissonSeq run fast. For RNA-seq data with ten replicates per group and 19,150 mRNAs, the slowest tools, baySeq and DESeq, were approximately 8000 and 2000 times slower than the fastest pipeline, limmaQN, respectively.

### Recovering biological truth

In addition to the concordance analysis, we also assessed the capability of the DE tools to recover genes with known biological evidence of DE in the benchmark datasets. To this purpose, results from three published studies were used to define the truth: genes with gender-biased expression [25], MYCN regulated genes [26], and TP53 pathway genes [27] (see “Methods” for description). The ability to recover the truth is evaluated using four metrics: number of recovered genes, similarity among DE pipelines in terms of the set of recovered genes, gene classification agreement with the truth, and GSEA. Detailed results can be found in Additional file 4.

Despite the challenge of defining biological truth, several pipelines show relatively good performance in recovering the known truth, definitely when considering that the experimental conditions are not identical in the benchmark studies and the truth studies. However, in terms of the number of recovered genes and the degree of similarity to each other, the pipelines show substantial variation. In line with the concordance analysis, conservative tools (DESeq, baySeq, and NOISeq) recovered a relatively lower number of genes with low similarity to other tools (Additional file 4: Figure S8). In contrast, tools such as SAMSeq and PoissonSeq that were categorized as liberal (highest number of SDE genes) according to the concordance analysis now ranked generally low in recovering the biological truth across the three control studies and exhibited the least agreement with other pipelines. Across the four metrics assessing biological truth, DESeq2 (both settings), edgeR (robust), and limma (voom+QW, voom, and trended) outperformed all other tools, whereas PoissonSeq, SAMSeq, NOISeq, DESeq, and QuasiSeq (both settings) showed inferior capability.

### Simulation results

The non-parametric SimSeq [28] procedure was applied to realistically simulate RNA-seq expression data. The simulation technique involves sub-sampling of replicates from a real RNA-seq dataset with a sufficiently large number of replicates. In this way, the underlying characteristics of the source dataset are preserved, including the count distributions and variability. The representativeness of the simulated data was examined using various quality metrics, including those proposed by Soneson and Robinson [29] (see “Methods” section). Three series of simulations were performed, each starting from a different RNA-seq source dataset: Zhang, NGP nutlin, and GTEx data. The degree of homogeneity among the replicates in these datasets varies, reflecting different levels of intra-group biological variability (Table 2 and Additional file 1: Figure S2). The Zhang and NGP nutlin datasets include annotated lncRNAs along with mRNAs, whereas the GTEx RNA-seq dataset contains only annotated mRNA genes. Therefore, simulated counts for mRNA and lncRNA are sampled from mRNA and lncRNA counts of the source dataset, respectively.

Gene expressions were simulated under a wide range of scenarios that may affect the performance of DE tools: different numbers of replicates ranging from 2 to 40, different proportions of true DE genes (0 to 30%), two gene biotypes (mRNA and lncRNA), and different levels of intra-group biological variability (as present in the three source datasets). From the simulation results, the actual FDR, true positive rate (TPR), and false positive rate (FPR) were computed for each DE pipeline. The comparison between the two gene biotypes was done in two ways: simulating lncRNA data only or simulating lncRNA and mRNA jointly, but analyzing separately.

### False discovery rate and true positive rate

FDR refers to the average proportion of incorrect discoveries among SDE genes (genes identified as DE at a particular nominal FDR threshold). A good DE tool has actual FDR close to the nominal level, and has high TPR. The TPR, also known as sensitivity, is the average proportion of SDE genes among the true DE genes. The TPR should be sufficiently large, otherwise one cannot expect to find many of the true DE genes. Therefore, it is customary to look for a DE pipeline that has the highest TPR among those that control FDR (i.e., actual FDR is close to nominal FDR). The FDR versus TPR curve is used to compare the performance of DE pipelines at various nominal FDR threshold (ranging from 0 to 100%).

**Fig. 4:**
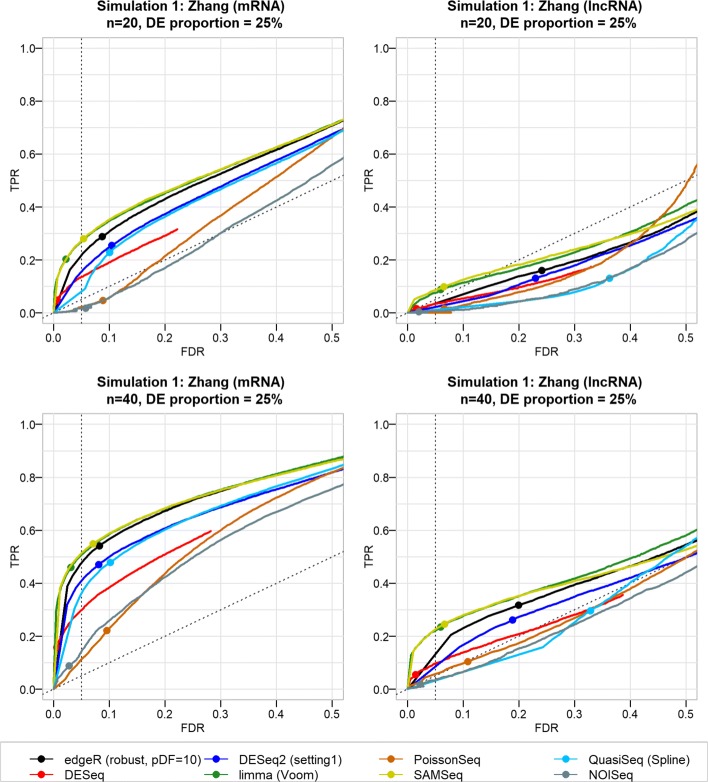
False discovery rate and true positive rate of DE tools using simulated data from the Zhang RNA-seq dataset. The actual FDR and TPR (at various nominal FDR) of eight DE tools from joint simulation and DGE analysis of mRNA and lncRNA. These particular results are from simulations with 25% true DE genes among 10,000 genes (constituting approximately 30% lncRNAs and 70% mRNAs) for designs with n = 20 and 40 replicates per group. The curves represent the trade-off between the average TPR and the average actual FDR at different nominal FDR (ranging from 0 to 100%). The points on the curve indicate the actual FDR and TPR values at 5% nominal FDR threshold. Although negative binomial models (edgeR, DESeq2, and QuasiSeq) showed higher sensitivity, in general they tend to lose FDR control for simulated data with lower numbers of replicates. In contrast, DESeq, NOISeq, and PoissonSeq showed better capability of controlling FDR, with actual FDR below the threshold level (5%), but these tools have lower sensitivity than all other DE tools. For simulated data with at least ten replicates per group, SAMSeq and limma tools consistently showed better FDR control and comparable TPR to negative binomial models (more results can be found in Additional file 1). DE pipelines generally exhibited substandard performance (high FDR and low TPR) for lncRNAs than for mRNAs

**Fig. 5:**
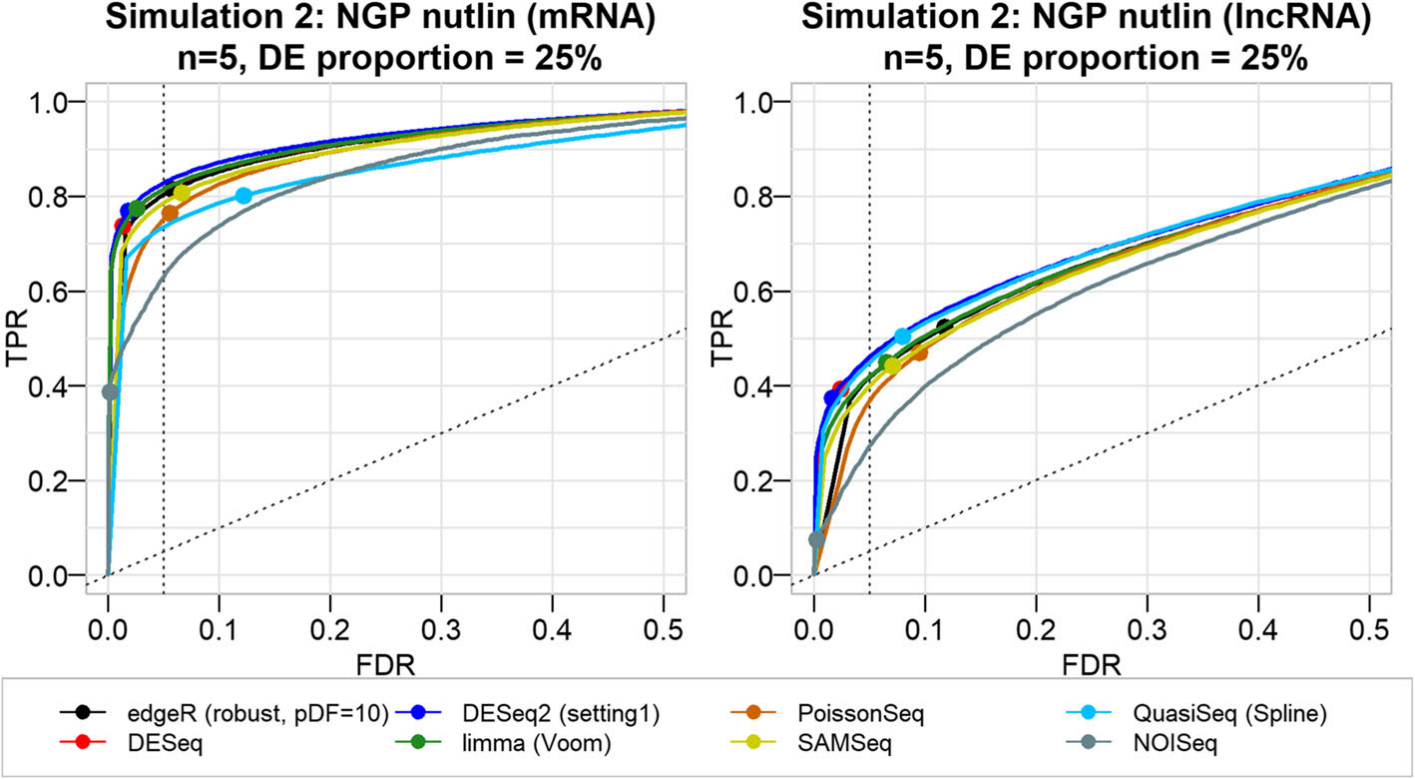
False discovery rate and true positive rate of DE tools using simulated data from the NGP nutlin datasets. The actual FDR and TPR (at various nominal FDR) of eight DE tools from joint simulation and DGE analysis of mRNA and lncRNA. These particular results are from simulations with 25% true DE genes among 10,000 genes (constituting approximately 35% lncRNAs and 65% mRNAs) for designs with replicates per group. The curves represent the trade-off between the actual FDR and TPR at different nominal FDR (ranging from 0 to 100%). The points on the curve indicate the actual FDR and TPR values at 5% nominal FDR threshold. In general, DE tools’ performance for gene expression data simulated from the NGP nutlin dataset is better (low FDR and high TPR) than the performance observed from the Zhang based simulation, which can be explained by the difference in the intra-group biological variability. In line with the first simulation, DE tools’ performance appeared to be relatively lower for lncRNAs than for mRNAs

Results from the first simulation (starting from the Zhang data) generally indicate that the FDR is not controlled well by many DE pipelines (Fig. 4). Among the pipelines that control the FDR relatively well, many have a small TPR. Besides the gene biotype (mRNA versus lncRNA), the performance is correlated with the level of the intra-group variability, the number of replicate samples, and the fraction of DE genes. Many DE tools show severe FDR inflation and slightly lower TPR when only a small fraction of genes is DE (Additional file 1: Figures S9 and S10). The actual FDR may even exceed 50%, which means that more than half of the called SDE genes may be false discoveries. For most DE tools, better FDR control and higher sensitivity were attained with increasing number of replicates (Fig. 4 and Additional file 1: Figures S11 and S12). Performance of all DE pipelines is considerably poorer for lncRNAs than for mRNAs (Figs. 4 and 5). However, very similar results (poor performance in terms of FDR control and TPR) were obtained for low-abundance mRNAs based on a simulation starting from the GTEx data (Additional file 1: Figure S13).

For the simulation that started from the (homogeneous) NGP nutlin data, the results were better (Fig. 5), with good FDR control and high TPR for all DE tools, even for small numbers of replicates. Only for simulations with 5% of true DE genes was the FDR control lost (Additional file 1: Figure S10). The difference in performance between the Zhang and NGP nutlin simulations can be explained by their intra-group variability (Table 2 and Additional file 1: Figure S2): the NGP nutlin data come from cell line replicates that are characterized by low biological variability. For the simulations starting from the GTEx dataset, which has intermediate biological variability, the performance of the DE tools is somewhere in between those for the Zhang and NGP nutlin datasets (Additional file 1: Figure S14).

Because of the trade-off between FDR and TPR, a high TPR is expected for DE tools with a high actual FDR. This was observed for edgeR, DESeq2, and QuasiSeq pipelines, particularly for small numbers of replicates (Fig. 4). limma and SAMSeq showed better FDR control, while retaining a high TPR. Their better performance is true for both biotypes with at least ten and four samples per group for the Zhang and NGP nutlin simulations, respectively (Additional file 1: Figures S11 and S12). DESeq, PoissonSeq, and NOISeq showed better FDR control, but at a cost of severe TPR loss.

Among the seven edgeR pipelines, edgeR robust showed generally better performance for the Zhang data simulations (Additional file 1: Figure S15). However, only a small difference was observed in the simulation that starts with the less variable NGP nutlin data. edgeR robust with data-specific prior degrees of freedom seems more beneficial in maximizing the TPR. Only small performance variation was observed among the limma pipelines, except limmaQN, which deviated substantially (lower performance) in the second simulation (Additional file 1: Figure S16). This deviation may be due to the number of replicates, as only five samples were used in each group. Among all limma pipelines except limmaQN, voom with sample quality weight (limmaVoom+QW) lost control of FDR. Similarly, minor differences were observed among the DESeq2 pipelines (Additional file 1: Figure S17). However, as indicated in the concordance analysis, the independent filtering should be used carefully for lncRNAs. Similarly, among the QuasiSeq pipelines, the one with QL dispersion estimated independently for each gene, appeared to have worse performance (Additional file 1: Figure S18).

The simulation study demonstrated that large heterogeneity among samples has a potential to negatively affect the performance of DE tools, particularly leading to a failure to detect biological signals. The heterogeneity can result from both biological and technical factors. The technical artefacts can be alleviated by filtering low quality or aberrant samples that substantially contribute to the intra-group variability [30]. Such samples can be recognized by the sample-to-sample distances projected into a two-dimensional space using, for example, principal component analysis [10, 32]. This is confirmed by an extra simulation that starts from the Zhang data whereby the most distant (outlying) samples were excluded beforehand (Additional file 1: Section 4.2.3). The results generally indicate that DE tools perform better with respect to FDR control and sensitivity if outlying samples are excluded (Additional file 1: Figures S19 and S20).

Methods for controlling the FDR, for example, Benjamin and Hochberg (BH) [31], rely on the assumption that the raw *p* values have a flat distribution near *p* = 1. This assumption, however, might not always hold, especially for low-abundance genes such as lncRNAs and for small numbers of replicates. This concern is demonstrated by (1) a simulation with no DE genes, so that all *p* values correspond to the null hypothesis, and (2) using the *p* values from the DE results from the six benchmark RNA-seq datasets. For comparison purposes, the *p* value distributions from the analysis of a simulated dataset with 30% DE genes is also included. The *p* values associated with the null hypotheses are supposed to be uniformly distributed between 0 and 1. For datasets with a fraction of SDE genes, a spike near *p* = 0 and a flat distribution near *p* = 1 is expected if the DE tool works fine. For many DE pipelines, the observed *p* value distribution looks as expected (Additional file 1: Figures S21–S27 and Additional file 2). When the number of replicates is small, a slightly conservative *p* value distribution (a spike near *p* = 1) is noticeable for lncRNAs, and to a lesser extent for mRNAs. The underlining cause may be the high variability of lncRNAs. This may result in loss of power to detect true DE lncRNAs, as confirmed by our simulation study. Correct calibration of *p* values under the null hypothesis and a large sample size can overcome this issue. Overall, QuasiSeq, DESeq, edgeR (exact test), and limma tools (for small numbers of replicates) return *p* values that do not well satisfy the assumption of *p* value uniformity.

### False positive rate

The FPR refers to the probability of calling a gene SDE in a scenario with no DE genes at all. FPR of DE tools was evaluated using a simulated RNAseq data with 0% DE genes (also known as mock comparison). Results shown in Additional file 1: Figure S28 demonstrate that all DE pipelines resulted in a FPR of less than 1%. The results were similar for both gene biotypes (mRNAs and lncRNAs), except for a slightly higher FPR for lncRNAs than for mRNAs. The FPR was generally larger for methods relying on the negative binomial distribution. This finding is in line with conclusions from a previous comparative study [13] in which it was concluded that the number of false predictions of differential expression from DE tools (most of these DE tools are also the part of our study) is sufficiently low even for genes with low counts (the lowest 25% expressed genes).

### Simulation of lncRNA expression data only

Results presented up to this point came from simulating, normalizing, and analyzing lncRNAs and mRNAs together. Of note, joint analysis of the two gene biotypes may affect results. For example, estimates of gene-specific dispersion parameters for negative binomial models are often done by sharing information across all genes using empirical Bays strategy [32–34], and hence the results for lncRNAs depend on mRNA read counts and vice versa. In addition, adjusted *p* values aimed at controlling FDR are calculated taking into account the total number of genes included in the analysis [31]. Therefore, we also evaluated the performance of the DE tools with only lncRNA data, using the same simulation procedures. Our conclusions remain the same. The results are shown in Additional file 1: Figure S29. The FDR control is generally worse when analyzing lncRNA separately, particularly for small replicate sizes. Only a small reduction in TPR is observed.

### Web application

All simulation results can be consulted and visualized with a web application [35].

## Conclusions

The discovery and study of lncRNAs is of major relevance to human health and disease because they represent an extensive, largely unexplored, and functional component of the genome [3–5]. Several gene expression studies indicated that the expression of the majority of lncRNAs is characterized by low abundance [2, 7, 9], high noise [8], and tissue-specific expression [7]. These characteristics are very challenging for DE tools and may potentially negatively affect tool performance [10, 11]. Our study evaluated the performance of widely used statistical tools for testing DGE in RNA-seq data, with separate analysis of mRNA and lncRNA.

Under the assumption that there is no batch effect, all considered normalization methods perform equally well with respect to correcting the library size differences and reducing technical variability. However, QN tends to substantially deviate in terms of its effect on DGE analysis. This result was also confirmed by the poor performance of the limma DE pipeline that uses QN. Therefore, it is fair to suggest not to use QN to normalize RNA-seq data. Concordance analysis based on six diverse types of RNA-seq datasets demonstrated that the DE pipelines lack strong consensus on identifying a set of significantly differentially expressed genes, gene ranking, and fold-change estimates. For datasets with a small number of replicates and/or heterogeneous replicates, the disagreement is even worse. Lower concordance was observed generally for lncRNAs than for mRNAs. In particular, limmaQN, NOISeq, baySeq, and DESeq (also PoissonSeq and SAMSeq to a smaller extent) showed lower concordance with other DE tools. In contrast, edgeR, DESeq2, and limma (except limmaQN) tools showed better agreement with other DE tools and consistent characteristics across datasets. In terms of recovering known evidence of a biological truth, DESeq2 (both settings), edgeR (robust), and limma (voom+QW, voom, and trended) showed better capability than all other tools, whereas PoissonSeq, SAMSeq, NOISeq, DESeq, and QuasiSeq (both settings) showed inferior capability.

Results of the non-parametric simulation study revealed that there are substantial differences between methods with respect to FDR control and sensitivity. The DE tool performance is strongly affected by the sample size and biological variability. FDR control at the nominal level is good for all methods for datasets with small biological variability, even with only five biological replicates per condition. On the other hand, for datasets that are more variable, the FDR control is only guaranteed for larger sample sizes, and only with the following methods: PoissonSeq, limma tools, SAMSeq, and DESeq. All other DE methods result in actual FDR levels far above the nominal level, up to an FDR exceeding 50% for lncRNAs even when studying 40 replicates per condition. Differences between the tools in terms of sensitivity are not very large, except for PoissonSeq, NOISeq, DESeq, and limmaQN to a smaller extent, which showed the lowest sensitivities among all pipelines. For highly variable data, a maximum sensitivity of 50% for mRNAs is obtained with at least 40 replicates per condition. For homogeneous samples, this level of sensitivity is reached with four to five replicates per condition. In addition, we have demonstrated that the performance of DE tools can be improved by filtering aberrant samples that substantially contribute to the intra-group variability.

In the light of promoting reproducible science, it is essential to select a DE tool that succeeds in controlling the FDR level under a large range of conditions. Among these DE tools, one can select one with a high sensitivity. If a DE tool has an actual FDR far larger than the nominal level, many of the claimed discoveries will be false discoveries. If one accepts a large proportion of false discoveries, it is generally better still to use a DE tool with good FDR control, but to apply the method at a larger nominal FDR level. In this way, the researcher still controls the error rate. This reasoning implies that the selection of a DE tool may never rely purely on the sensitivity. High sensitivities may be expected from methods with a large actual FDR (and hence poor FDR control). These high sensitivities are illusive in the light of the large proportion of false discoveries.

Combining all results, we conclude that limma (with variance stabilizing transformation; voom with or without quality weighting; trend) and SAMSeq control the actual FDR reasonably well, while not sacrificing sensitivity. However, desirable performance is guaranteed only for a reasonably large number of replicates and for samples with low variability. Our results also indicate that accurate differential expression inference of lncRNAs requires more samples than that of mRNAs. Although we concluded that DE tools exhibit substandard performance for lncRNAs, the substandard performance of DE tools also applies to low-abundance mRNAs.

## Methods

### RNA-seq datasets

A total of six datasets are used in this study. All of them are publicly accessible. The Zhang dataset, containing 498 neuroblastoma tumors, was retrieved from Zhang et al. [36] (GEO accession number GSE49711). In short, unstranded poly(A)+ RNA sequencing was performed on the HiSeq 2000 instrument (Illumina). Paired-end reads with a length of 100 nucleotides were obtained. To quantify the full transcriptome, raw fastq files were processed with Kallisto [37] v0.42.4 (index build with GRCh38-Ensembl v85). The pseudo-alignment tool Kallisto was chosen above other quantification methods as it is performing equally good but faster. For this study, a subset of 172 patients with high-risk disease were selected, forming two groups: the MYCN amplified (*n*_1_ = 91) and MYCN non-amplified (*n*_2_ = 81) tumors. Twenty samples randomly selected from each group were used to study concordance analysis of the DE tools, whereas all samples were considered for the simulation study.

Second, the NGP nutlin-3 dataset (GEO accession number GSE104756) contains ten biological replicates of NGP neuroblastoma cells grown in six-well culture flasks treated with either nutlin-3 (8M racemic mixture) or vehicle (ethanol). RNA was extracted using the RNeasy mini kit (Qiagen), quantified using Nanodrop-100, and quality controlled using FragmentAnalyzer (High Sensitivity RNA Analysis Kit, DNF-472-0500, Advanced Analytical). Total RNA (100 ng) was used for TruSeq stranded mRNA library prep (Illumina), followed by paired-end sequencing (2 × 75 nucleotides) on a NextSeq 500 instrument (Illumina). On average, 20 million read pairs per sample were generated. Raw fastq files were processed with Kallisto2 v0.42.4 (index build with GRCh38-Ensembl v85).

From the Gene Expression Omnibus (GEO) repository of analysis-ready RNA-seq dataset [38], we accessed two datasets: Hammer (GEO accession number GSE20895) and Bottomly mRNA-seq datasets (GEO accession number GSE26024). The Hammer mRNA-seq dataset contains the results of a study on the L4 dorsal root ganglion (DRG) of rats with chronic neuropathic pain induced by spinal nerve ligation (SNL) of the neighbouring (L5) spinal nerve (two controls and two with L5-SNL induced chronic neuropathic pain) [39]. The study includes RNA-seq samples of each treatment at two different time points, and in our study we only used samples from the first time point. The Bottomly mRNA-seq data are an expression set obtained from the brain striatum tissues of two mice strains (it includes ten biological replicates of the C57BL/6J (B6) strain and 11 biological replicates of the DBA/2J (D2) strain) [40]. Details about library preparation and features of these datasets can be obtained from the original papers cited above.

We also used a subset of the RNA-seq dataset from the GTEx (Genotype-Tissue Expression) project [41], which provides a large database of human gene expression data with more than 3000 RNA-seq samples from 54 different tissues. For our study, we selected all biological replicates of hippocampus (*n*_2_ = 28) and hypothalamus (*n*_1_ = 30) mRNA-seq counts. All information about this dataset can be obtained from the GTEx project database. Twenty samples randomly selected from each tissue were used for concordance analysis of the DE tools and all samples were considered for the third simulation study.

The colorectal cancer (CRC AZA) RNA-seq dataset contains three biological replicates of HCT-116 cells grown in six-well culture flasks treated with either azacitidine (1 μM) or vehicle (DMSO). RNA was extracted using the RNeasy mini kit (Qiagen), quantified using Picogreen, and quality controlled using Bioanalyser FragmentAnalyzer. Total RNA (500 ng) was used for TruSeq stranded mRNA library prep (Illumina), followed by paired-end sequencing (2 × 100 nucleotides) on a HiSeq instrument (Illumina). On average, 40 million read pairs per sample were generated. Raw fastq files were processed with Bowtie2/Cufflinks (index build with GRCh37-Ensembl v75). The count data can be accessed in Additional file 6 [42].

### DE tools and normalization methods

Five normalization methods were evaluated in this study: quantile normalization (QN) [43] implemented in limma (limmaQN), Trimmed Mean of M-values (TMM) [44] implemented in edgeR, limma (limmaVoom, limmaVoom+QW, and limmaTrended), baySeq, NOISeq, and QuasiSeq, Medians of ratios [45] implemented in DESeq, DESeq2, and limma (limmaVst), the goodness-of-fit statistics [46] approach in PoissonSeq, and the Poisson re-sampling technique [19] implemented in SAMSeq. To examine the effect of normalization on DGE analysis, we applied moderated *t*-test of the limma package in combination to each of the five normalization methods. Afterwards, the extent of dissimilarity of results was used as an indicator of normalization effect on DGE analysis.

Nine DE tools were evaluated through a total of 25 pipelines (various settings of the DE tools). Seven edgeR pipelines were evaluated: (1) exact test based on negative binomial distribution (edgeR exact, also known as edgeR classic) [47], (2) generalized linear model for multi-factor design (edgeR GLM) [48], (3–6) robust GLM [49], and (7) quasi-likelihood method (edgeR QL) [50]. edgeR robust was run with four different prior degrees of freedom (pDF) to estimate robust gene-wise dispersions: pDF = 10 (the default), 5, 20, and estimated pDF obtained using the *getPriorN()* function of the edgeR package. The latter is referred to us “pDF=auto”. The pDF determines the degree of shrinkage of gene-wise dispersions towards the common estimate. The shrinkage influences the precision of the DE inference, and it is indicated to consider various pDF for different data, depending on the number of samples and complexity of the design [11, 32].

DESeq2 [10] was applied with three different settings. The default DESeq2 pipeline applies independent filtering of low-abundance genes prior to calculating the FDR. It also flags outliers using Cook’s distance. It replaces flagged observations by imputed values if the number of samples is larger than 6, or excludes otherwise. Therefore, besides the default setting, two pipelines were considered: the first setting disables independent filtering (setting 1); the second disables both independent filtering and outlier detection (setting 2).

Similarly, seven limma pipelines were tested: (1) limma-voom [51], (2) limma-trended [51], in which the mean-variance trend is incorporated into the empirical Bayes moderation. Both voom and trended limma pipelines were also applied with a robust estimation of pDF and variance as separate pipelines (3 and 4). (5) limma voom was also applied in conjunction with sample quality weights (limmaVoom+QW) to down-weight low quality samples [30]. Other limma pipelines are (6) limma with QN (limmaQN) [13] and (7) limma with variance stabilizing transformation using DESeq tool (limmaVst) [14].

QuasiSeq [34] applies quasi-likelihood inference to test DE with three different proposed pipelines to estimate gene-specific dispersion (referred to as quasi-dispersion): (1) independent estimates (QL), (2) shrinkage towards a common estimate (QLShrink), and (3) spline-smoothing (QLSpline). DESeq [45], baySeq [52], PoissonSeq [46], SAMSeq [19], and NOISeq (particularly NOISeqBIO) [53] were applied with their default settings. For PoissonSeq the filtration cut-point for the sum and average counts of genes across samples were set to 1 and 0.01, respectively.

All computations were performed in R version 3.3.2 [12]. baySeq was not included in the simulation study due to its slow computation time. The reader can also access the R scripts in Additional file 6 [42].

### Concordance analysis

The numbers of SDE genes detected for each dataset were compared among the 25 DE pipelines. For the Zhang and NGP nutlin datasets, we explored the proportion of mRNAs and lncRNAs within the set of SDE genes. For the other datasets, we explored the proportion of low and high expressed mRNAs among the set of SDE mRNAs. In particular, we divided the genes into four equally large groups based on the quartiles of their average normalized read counts across samples (DESeq normalization). To study the concordance in SDE calling between any two DE pipelines, we calculated the extent of overlap as the proportion of SDE genes commonly detected by the two tools. To rank genes in the order of significance of differential expression, taking into account the biological importance of the significance, we calculated the *π* score [24]:

$$ {\pi}_i=-{\log}_{10}{p}_i\times {\varphi}_i $$

where *φ*_*i*_ is the absolute value of the estimated LFC for the *i*^*th*^ gene and *p*_*i*_ is the raw *p* value, except for SAMSeq, for which *p*_*i*_ is replaced by *w*_*i*_^− 1^, where *w* is the Wilcoxon statistic [19]. For baySeq, *p*_*i*_ is the estimated Bayesian false discovery rate (BFDR) [52]. For NOISeq, *p*_*i*_ is replaced by an equivalent measure of the significance as proposed by the authors (1 − the probability of DE) [53]. Afterwards, we used Spearman’s rank correlation to evaluate the degree of agreement among DE pipelines with respect to gene ranking.

### Recovering biological truth

Results from three published studies were used to define the truth. (1) A list of 54 genes with sex-biased expression evidence using microarray data in at least one central nervous system region (passing FDR 1%) were obtained from Trabzuni et al. [25]. The ability of the DE tools to recover these genes was examined by comparing two groups of neuroblastoma samples of the Zhang data: girls (*n*_1_ = 44) versus boys (*n*_2_ = 48). (2) MYCN is a bona fide oncogenic transcription factor, activated in high-risk neuroblastoma through high-level gene amplification. A set of 157 MYCN pathway genes was obtained from a microarray study on primary tumors and shRNA model systems [26]. By comparing MYCN amplified (*n*_1_ = 20) and MYCN not-amplified neuroblastoma (*n*_2_ = 20) samples from the Zhang RNA-seq data, the ability of the DE pipelines to recover the 157 MYCN pathway genes was examined. (3) TP53 is the most frequently mutated and best studied gene in cancer. The protein itself is a transcription factor regulating a set of genes involved in cell-cycle control and programmed cell death. The consensus between SDE genes (identified by the 25 DE pipelines from analysing the NGP nutlin data at 1% FDR) and the TP53 pathway was examined using a set of 116 TP53 pathway genes obtained from a meta-analysis consensus list [27]. Nutlin-3a liberates TP53 from MDM2-mediated inhibition and hence activates the TP53 transcriptional response pathway.

To come to an overall conclusion, consensus ranking of the 25 DE pipelines was established using ConsRank (version 2.0.1) R software package [54]. A detailed description of methods and results is available in Additional file 4.

### Non-parametric simulation methods

SimSeq [28] simulates a matrix of RNA-seq read counts by sub-sampling samples from a source RNA-seq dataset. It creates a set of DE genes based on prior information from analyzing the source dataset (which has a larger number of replicates than the simulated expression data). Details about the method are given in Benidt and Nettleton [28] and in Additional file 1: Section 4.1.

Three sets of simulations were performed, each starting from a different source dataset: the Zhang, NGP nutlin, and GTEx datasets. Gene expression levels are simulated containing a given proportion of true DE genes (ranging from 0 to 30%) from a total of 10,000 genes. Each gene in every simulated count matrix has a total of at least one expression in each group. Also, counts are simulated for varying numbers of replicates per group (2 to 40, 2 to 5, and 2 to 14 for simulations that start from the Zhang, NGP nutlin, and GTEx datasets, respectively). For joint simulation of mRNAs and lncRNAs (for Zhang- and NGP nutlin-based simulations), each simulated count matrix includes a number of lncRNAs and mRNAs in the set of true DE genes and non-DE genes, proportional to their numbers in the source dataset (for the Zhang data 70% mRNA and for NGP nutlin data 65% mRNA). We ran 100 independent simulations for each scenario. On each simulated dataset, we applied the 24 DE pipelines and calculated *p* values, adjusted *p* values, LFC, and other relevant statistics. These statistics were subsequently used for computing the comparative performance metrics.

For a given fraction of true DE genes, the SimSeq method generates data for which it is known which genes are truly DE. To each simulated dataset all DE tools are applied and genes are called SDE if their FDR-adjusted *p* values are smaller than the nominal FDR level (nominal FDR ∈ [0, 1]). For a single simulated dataset the observed false discovery proportion (FDP) is calculated as *FDP* = *FP*/(*FP* + *TP*), where *FP* and *TP* are the numbers of incorrect (false) and correct (true) rejection of the null hypothesis, respectively. The actual false discovery rate (FDR) is then computed as the average of the FDPs over all simulations. When the FDR is computed for a scenario with no DE genes at all, it is generally known as the false positive rate (FPR). For this scenario, the SimSeq procedure simply samples replicates from only one group of the source dataset and randomly assigns the samples to one of two arbitrary mock groups. The actual true positive rate (TPR), which is also called sensitivity, is defined as the average of the true positive proportions (*TPP*) across independent simulations, *TPP* = *TP*/(*TP* + *FN*), where *FN* is the number of incorrect decisions to fail rejecting the null hypothesis.

To demonstrate the importance of pre-processing of samples in reducing the intra-group variability (and hence improve the performance of DE tools), we ran an extra simulation that starts from the Zhang data (which contains the most variable samples) by filtering a set of outlying samples beforehand. We particularly used principal component analysis to identify such samples. After excluding the top 10% most extreme samples (in a two-dimensional space formed by the first two principal components) we applied the non-parametric simulation procedure on the remaining samples. Details can be found in Additional file 1: Section 4.2.3.

The quality of simulated data was examined using the quality metrics proposed by Soneson and Robinson [29] as implemented by their countsimQC R package (version 0.5.2). The metrics evaluate the average expressions of genes, variability, mean-variance relationship, correlations among replicates, correlations among genes, and fraction of zero counts. The quality assessments were positive in all aspects, and the reports generated by the countsimQC R package can be found in Additional file 5A. In addition, the representativeness of the simulated mRNA and lncRNA expression data is further explored using similar quality metrics (see Additional file 5B).

### Web application

A web application has been developed using the R Shiny package (version 1.0.5) [55]. This user interface allows the user to consult and visualize all detailed results from the simulation studies. It can be accessed at [35].

## Additional files

Additional file 1: Supplementary figures and methods. Figures and tables that are directly referenced in the main report. Review of previous comparative studies and additional descriptions of results and methods. (PDF 2971 kb)

Additional file 2: Supplementary data. Detailed results of comparisons of normalization methods. (HTML 2067 kb)

Additional file 3: Supplementary data. Detailed results of concordance analysis of DE tools. (HTML 15901 kb)

Additional file 4: Supplementary data. Detailed results from the study of DE tools’ recovering ability of biological truth. (HTML 2102 kb)

Additional file 5: Supplementary data (A and B). Results of simulation quality assessment. (ZIP 8405 kb)

## Abbreviations

AZA: Azacitidine
BCV: Biological coefficient of variation
CRC: Colorectal cancer
DGE: Differential gene expression
DE: Differential expression
EB: Empirical Bayes
FDR: False discovery rate
FDP: False discovery proportion
FPR: False positive rate
GEO: Gene Expression Omnibus
GLM: Generalized linear modeling
GTEx: Genotype-Tissue Expression
LFC: Log fold change
lncRNA: Long non-coding RNA
mRNA: Messenger RNA
NB: Negative binomial
QL: Quasi-likelihood
QN: Quantile normalization
QW: Quality weight
RNA: Ribonucleic acid
SDE: Significantly differentially expressed
STN: Signal-to-noise
TMM: Trimmed means of M values
TPR: True positive rate
VST: Variance stabilizing transformation
