# Supplementary material for: Differential gene expression analysis tools exhibit substandard performance for long non-coding RNA-sequencing data
Source: Genome Biol. 2018 Jul 24;19:96. doi: 10.1186/s13059-018-1466-5 (PMC6058388; doi:10.1186/s13059-018-1466-5)
Supplement: Supplementary file 1 — Supplementary figures and methods. Figures and tables that are directly referenced in the main report. Review of previous comparative studies and additional descriptions of results and methods. (PDF 2971 kb) [file 13059_2018_1466_MOESM1_ESM.pdf]

Additional File 1

Alemu Takele Assefa*[1], Katrijn De Paepe†[4], Celine Everaert‡[3], Pieter Mestdagh§[3], Olivier Thas¶[1,2] and Jo Vandesompele‖[3]

[1]Department of Data Analysis and Mathematical Modeling, Gent University, Belgium
[2]National Institute for Applied Statistics Research, University of Wollongong, Australia.
[3]Department of Pediatrics and medical Genetics, Gent University, Belgium
[4]Global Advanced Analytics Group, Bain & Company Belgium, Inc., Belgium

June 5, 2018

Contents

1 Summary of previous comparative studies **2**

2 Datasets **4**

3 Concordance analysis **6**

3.1 Sensitivity to outliers 8

3.2 Genes expressed only in one group 11

3.3 Computation time 13

4 Simulation study **15**

4.1 SimSeq simulation procedures 15

4.2 Additional simulation results 17

*AlemuTakele.Assefa@UGent.be
†Katrijn.DePaepe@Bain.com
‡Celine.Everaert@UGent.be
§Pieter.Mestdagh@UGent.be
¶Olivier.Thas@UGent.be
‖Jo Vandesompele@UGent.be

4.2.1 False discovery and true positive rates 17
4.2.2 Performance of DE tools for low count mRNAs 21
4.2.3 Sample filtering 27
4.2.4 Distribution of P values 30
4.2.5 False positive rates 37
4.2.6 Separate versus joint analysis of lncRNA and mRNA 38

1 Summary of previous comparative studies

Seven comparative studies, including this study, are summarized in Figure S1 in terms of the number of tested DE pipelines, the number of RNA-seq datasets used, the number of performance evaluation metrics, and consideration of low-expressed mRNAs or lncRNAs. In addition, Table S1 summarizes the six previous comparative studies on differential gene expression analysis tools for RNA-seq data, particularly for mRNAs. The summary includes the simulation method, the performance comparison metrics, and the recommended DE tools.

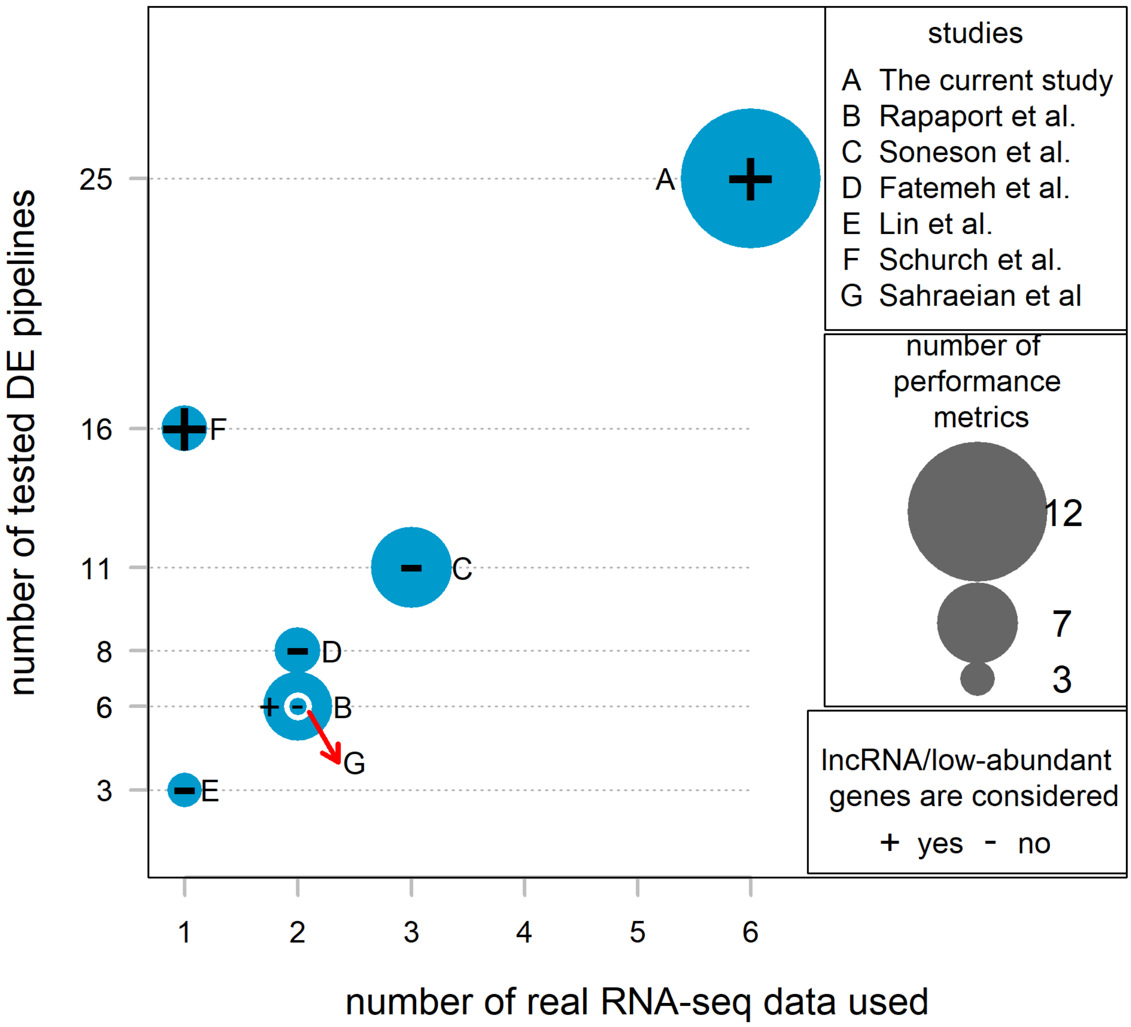

Figure S1: **Summary of previous DE tool comparison studies.** Seven comparative studies (including this study) on the performance of DE tools for RNA-seq dataset are summarized in terms of the number of DE pipelines tested (y-axis), the number of benchmark RNA-seq datasets used (x-axis), the number of performance metrics used (size of the bubble), and explicit consideration of lncRNAs or low-expressed genes in general (+/- inside bubble). The studies are labelled by letters: the current study (A), Rapaport et al (B) [1], Soneson et al (C)[2], Fatemeh et al (D)[3], Lin et al (E)[4], Schurch et al (F)[5], and Sahraeian et al (G)[6]. Further information about the studies can be found in Table S1.

Table S1: **Summary of previous DE tool comparison studies.** The table summarizes the method of simulation study (if any), performance metrics used, and the recommended DE pipelines from each comparative study. (Abbreviations: DE = differential expression, SEQC = Sequencing Quality Control Consortium, AUC = area under the operating characteristics curve, TPR = true positive rate, FNR=false negative rate, FPR=false positive rate, TNR=true negative rate.)

**study**	**simulation method**	**comparison metrics**	**recommended DE tools**
Rappaport et al[1]	using SEQC experimental dataset	sensitivity, specificity, performance on the subset of genes that are expressed only in one condition, performance at different sequencing depth and number of replicates.	(i) DESeq, edgeR, and baySeq, (ii) limma for large number of replicates, (iii) PoissonSeq and limma for genes expressed in one condition
Soneson et al[2]	gene exprression data from Negative Binomial and Poisson distribution	AUC, type I error rate, false discovery rate, computational time, concordance analysis (gene ranking agreement and overlap in DE classification)	variance-stabilizing transformation combined with limma (limmaVoom and limmaVst), SAMseq
Schurch et al[5]	comparing the DE results from subsets of replicates against the gold standard set of DE results obtained for each tool with the full set of clean replicates. The tool-specific gold standards were computed by running the tool on the full set of clean data.	TPR (sensitivity), FNR, FPR, TNR(specificity)	EBSeq, edgeR (exact), limma, and DESeq
Sahraeian et al[6]	using SEQC experimental dataset	sensitivity, and accuracy of expression change prediction	DESeq2
Fatemeh et al[3]	using multiple replicates within the sample groups by constructing artificial two-group comparisons to calculate proportion of false discoveries	number of detections, consistency within and between pipelines, proportion of false discoveries and the runtime	DESeq and limma
Lin et al[4]	based on real data analysis	power to accurately detect DE genes	edgeR and DESeq

2 Datasets

A total of 6 RNA-seq datasets were considered in our comparative study. All datasets contain biological replicates. The extent of biological variability (in terms of the estimated biological coefficient of variation (BCV)) in each dataset is summarized in Figure S2. Gene specific BCV was approximated by the estimates of the dispersion parameter of the negative binomial distribution. *edgeR* bioconductor package [7] was particularly used to obtain the BCV estimates (trended dispersions). The empirical distribution of gene-wise average expressions (log-transformed normalized counts) of mRNA and lncRNA are presented in Figure S3. The distributions are shown for the NGP nutlin and Zhang datasets as these two datasets contain both annotated mRNA and lncRNAs.

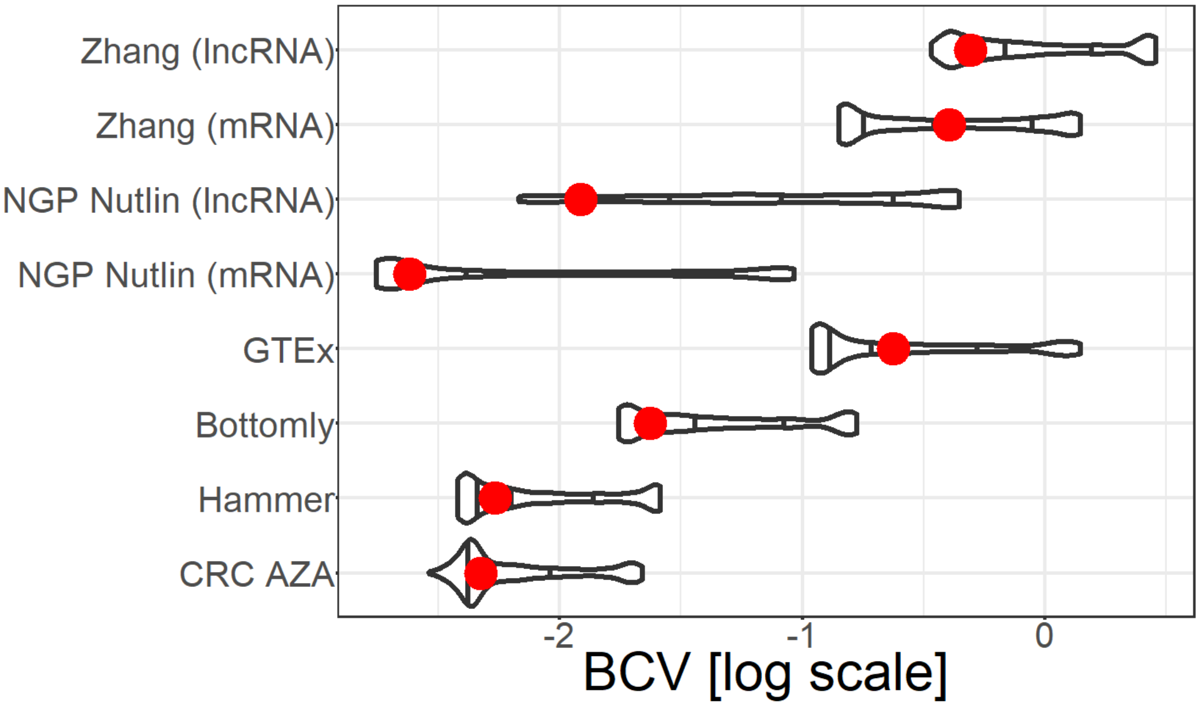

Figure S2: **Biological coefficients of variation (BCV).** The violin plots demonstrate the distribution of the estimated gene-specific BCVs in each RNA-seq dataset. For the Zhang and NGP nutlin datasets, the estimates for lncRNA and mRNA were obtained and presented separately. The red dots over each violin plot indicate the common dispersion estimate for all genes ($=\sqrt{\hat{\phi}}$, where ϕ is the common dispersion parameter of negative binomial distribution). The Zhang and GTEx datasets showed larger variability compared to the other datasets. In the Zhang and NGP nutlin datasets, higher BCV was observed for lncRNAs than for mRNAs.

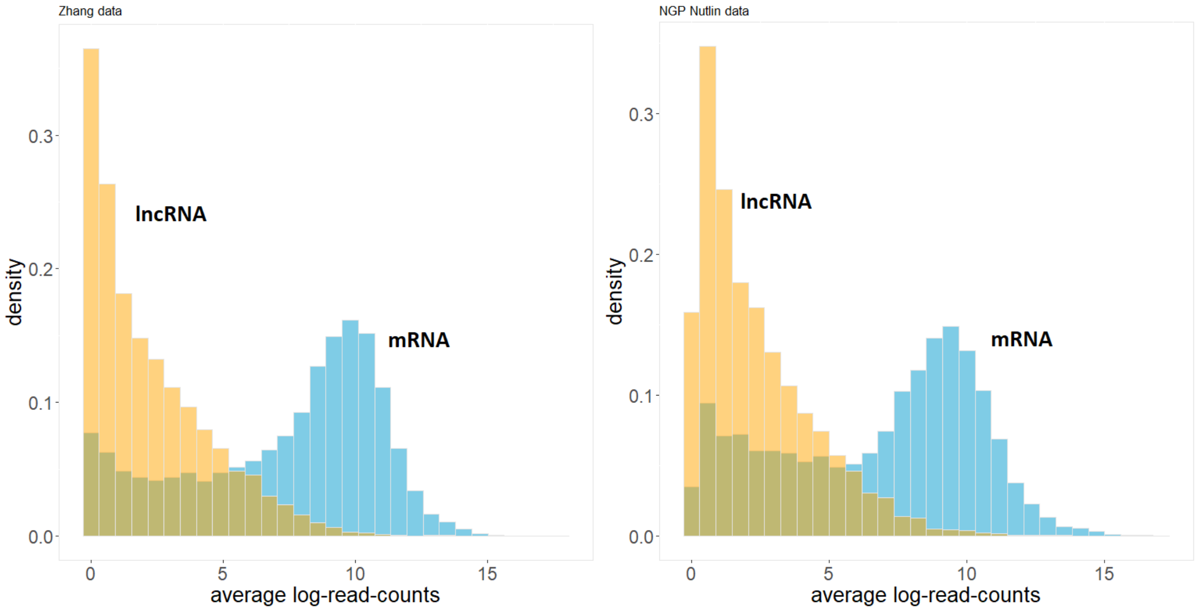

Figure S3: **Empirical distribution of average normalized counts for mRNAs and lncRNAs.** Empirical distribution of average normalized counts for mRNAs and lncRNAs. The histograms show the distribution of gene-wise average of $\log_2$-normalized-counts of mRNAs and lncRNAs from the Zhang and NGP nutlin datasets.

3 Concordance analysis

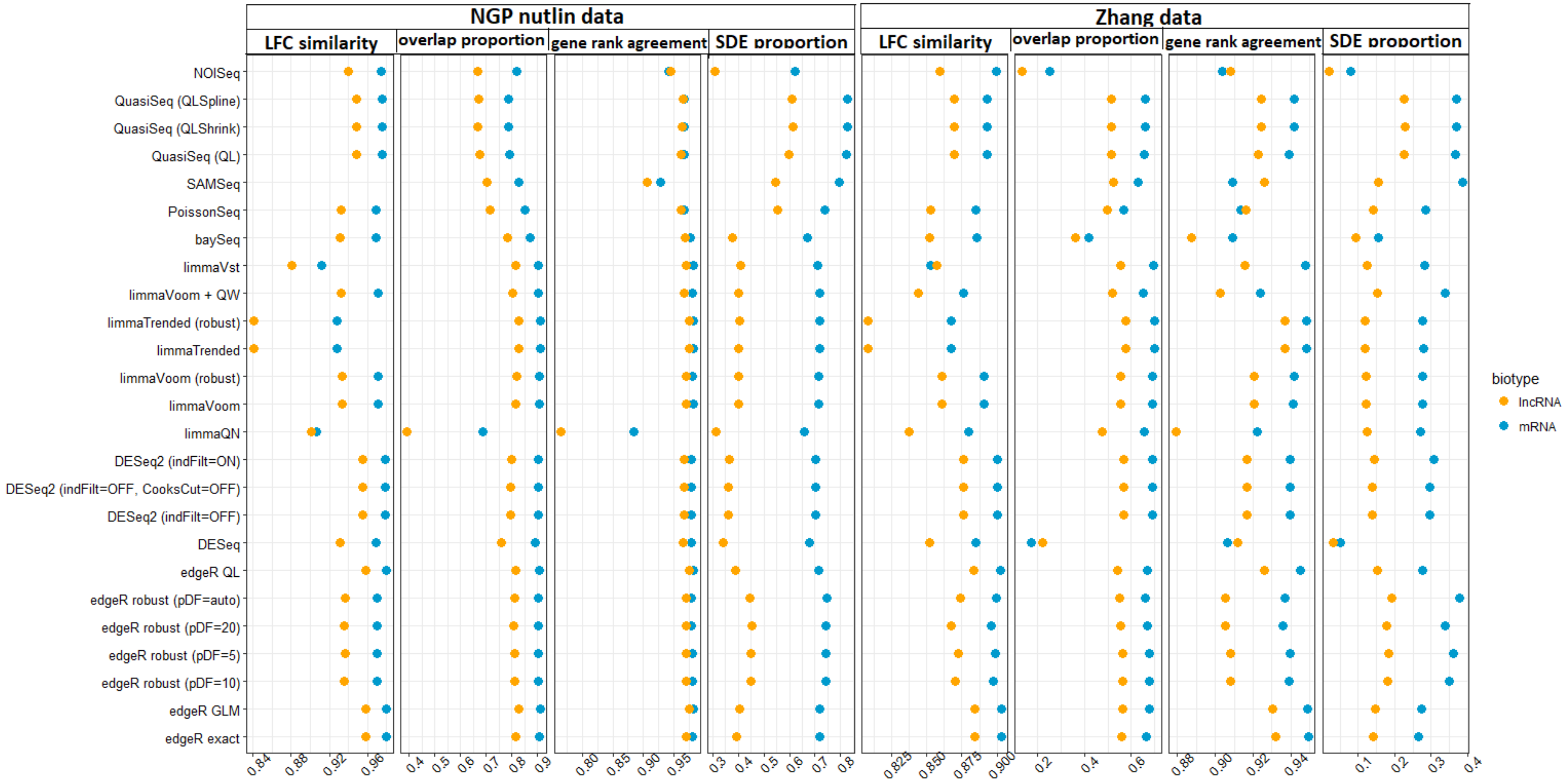

Figure S4: Concordance analysis of the 25 DE pipelines for mRNA and lncRNA based on the actual scores of the four concordance metrics (without standardizing) obtained from the analysis of the Zhang and NGP datasets.

3.1 Sensitivity to outliers

Because of either technical or biological reasons, RNA-seq data often contains genes with outlier counts across samples. Such a scenario is likely for lncRNAs due to their inherently noisy [8] or condition-specific expression [9]. DE tools may be sensitive to such outlier counts. Therefore, we qualitatively inspected the performance of DE tools for genes with extreme counts in one of the samples. We picked 8 such genes from the Zhang dataset (see the relative abundance of these genes in Figure S5). Outlier counts were defined as 5 median-absolute-deviations (MADs) [10] of relative abundances from the median. Further, the p-values from the Mann-Whitney and Wilcoxon rank sum test was larger than 0.05 when comparing the two groups with and without the outlier. The adjusted p-values returned from the 25 DE pipelines (for the selected 8 outlier genes) showed that edgeR exact, edgeR GLM, edgeR QL, PoissonSeq, QuasiSeq (both settings), and baySeq declared most of them SDE at 5% nominal FDR (Table S2) suggesting that they are sensitive to outlier expression. This conclusion is supported by the result from DE tools that are particularly developed to better cope with outliers, such as SAMSeq and edgeR robust, which declared none of the outlier genes as SDE.

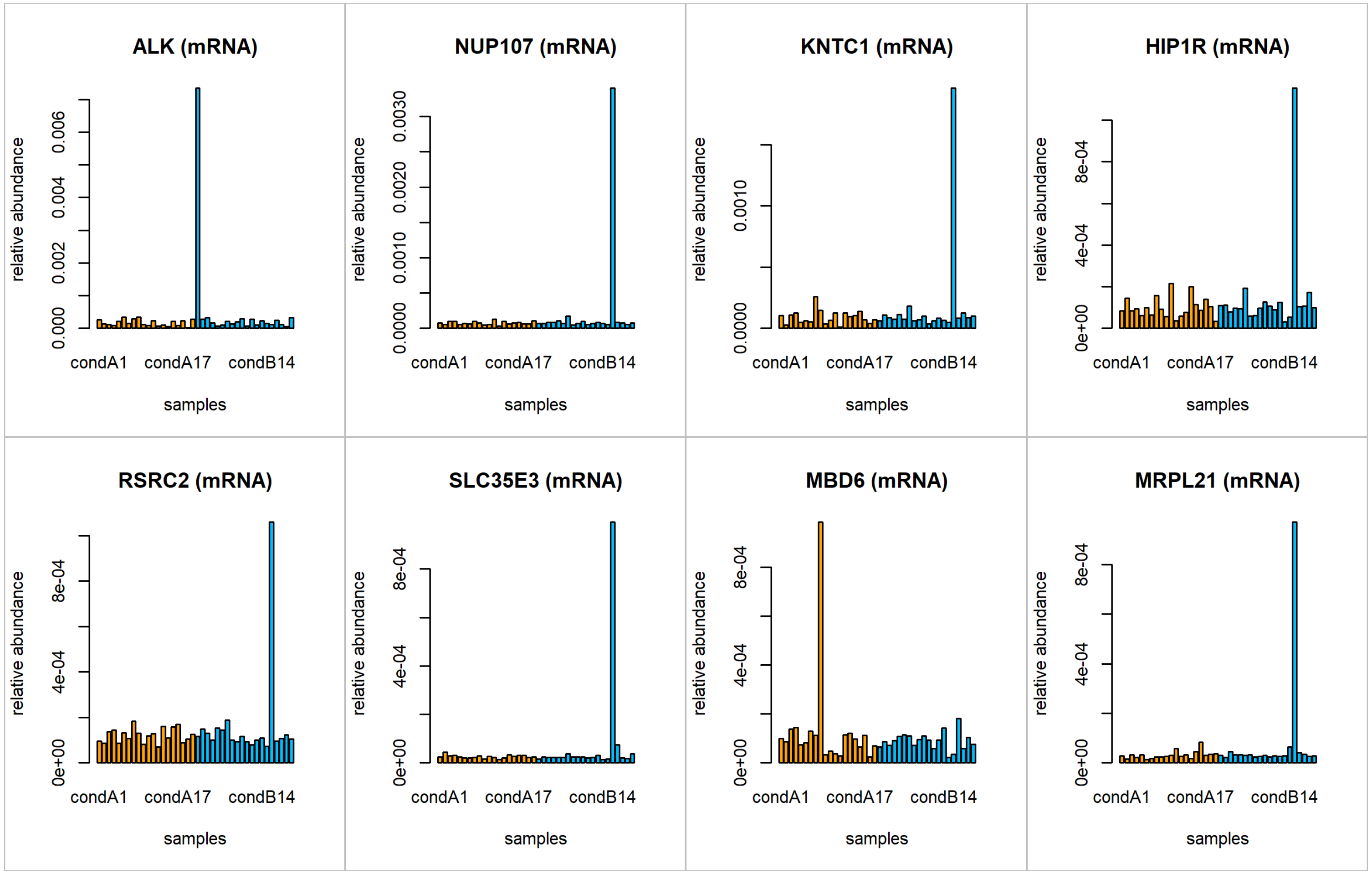

Figure S5: **Sensitivity to outliers:** The relative abundance of 8 genes with outlier expression in one of the samples. The colors represent the conditions (MYCN amplified and not-amplified). As it can be clearly seen, these genes have outlier expression in one of the samples.

Table S2: **Sensitivity to outliers:** Adjusted p-values returned from the 25 DE pipelines for the selected 8 genes with outlier count in one of the samples of the Zhang dataset.

	ALK	*NUP107*	*KNTC1*	*HIP1R*	*RSRC2*	*SLC35E3*	*MBD6*	*MRPL21*	#rejection*
edgeR robust (pDF=10)	0.403	0.394	0.792	0.480	0.901	0.867	0.858	0.473	0
edgeR robust (pDF=5)	0.421	0.377	0.789	0.479	0.893	0.864	0.854	0.458	0
edgeR robust (pDF=20)	0.369	0.416	0.794	0.477	0.909	0.870	0.863	0.491	0
edgeR robust (pDF='auto')	0.440	0.353	0.786	0.478	0.883	0.872	0.848	0.439	0
DESeq	0.704	0.757	0.767	0.742	0.803	0.758	0.977	0.757	0
DESeq2 (setting 1)	0.738	0.679	0.989	0.962	0.900	0.831	0.732	0.931	0
DESeq2 (setting 2)	0.738	0.679	0.989	0.962	0.900	0.831	0.732	0.931	0
DESeq2 (defaukt)	0.665	0.608	0.970	0.904	0.828	0.755	0.659	0.865	0
limmaQN	0.393	0.334	0.432	0.421	0.620	0.531	0.965	0.373	0
limmaVoom	0.309	0.305	0.337	0.336	0.523	0.450	0.913	0.316	0
limmaVoom (robust)	0.310	0.306	0.338	0.336	0.524	0.451	0.913	0.317	0
limmaTrended	0.324	0.310	0.355	0.374	0.540	0.471	0.950	0.339	0
limmaTrended (robust)	0.322	0.310	0.355	0.374	0.541	0.472	0.950	0.340	0
limmaVoom + QW	0.242	0.348	0.550	0.259	0.677	0.488	0.938	0.368	0
limmaVst	0.384	0.361	0.442	0.448	0.655	0.504	0.989	0.425	0
SAMSeq	0.387	0.326	0.482	0.349	1.000	1.000	1.000	0.463	0
NOISeq	0.111	0.107	0.173	0.220	0.292	0.128	0.264	0.160	0
edgeR QL	0.009	0.003	0.031	0.069	0.099	0.004	0.359	0.011	5
QuasiSeq (QL)	0.007	0.003	0.028	0.058	0.079	0.005	0.150	0.010	5
QuasiSeq (QLShrink)	0.005	0.002	0.023	0.051	0.072	0.003	0.146	0.008	5
QuasiSeq (QLSpline)	0.005	0.002	0.023	0.050	0.071	0.003	0.145	0.007	5
edgeR exact	0.001	0.000	0.010	0.041	0.076	0.000	0.335	0.002	6
edgeR GLM	0.001	0.000	0.009	0.038	0.071	0.000	0.320	0.002	6
baySeq	0.001	0.000	0.024	0.056	0.000	0.000	0.052	0.000	6
PoissonSeq	0.003	0.005	0.012	0.024	0.071	0.017	0.392	0.017	6

* number of SDE genes (adjusted p-values < 0.05)

3.2 Genes expressed only in one group

All results presented up to this point (including the simulation results) considered genes expressed in both groups of samples. However, it is quite common to notice a subset of genes detected only in one group of samples in RNA-seq data especially for low abundance genes such as lncRNAs. Such subset of genes can be problematic to DGE analysis as demonstrated by Rapaport et al [1]. To explore the ability of DE tools in identifying DE genes among such subset of genes, we randomly selected 200 genes from the Zhang data and changed their observed counts from one of the groups (also randomly chosen) to zero. This enables us to explore the ability of the tools at wider range of signal-to-noise (STN) ratio ($\frac{\mu}{\sigma}$, where μ and σ are the mean and standard deviation of normalized counts (from the non-zero group), respectively). The p-values and LFC estimates of such subset of genes from the 25 DE pipelines can be accessed at https://github.com/CenterForStatistics-UGent/Additional-File-6.git. The relationship between the adjusted p-value and STR for these genes is presented in Figure S6.

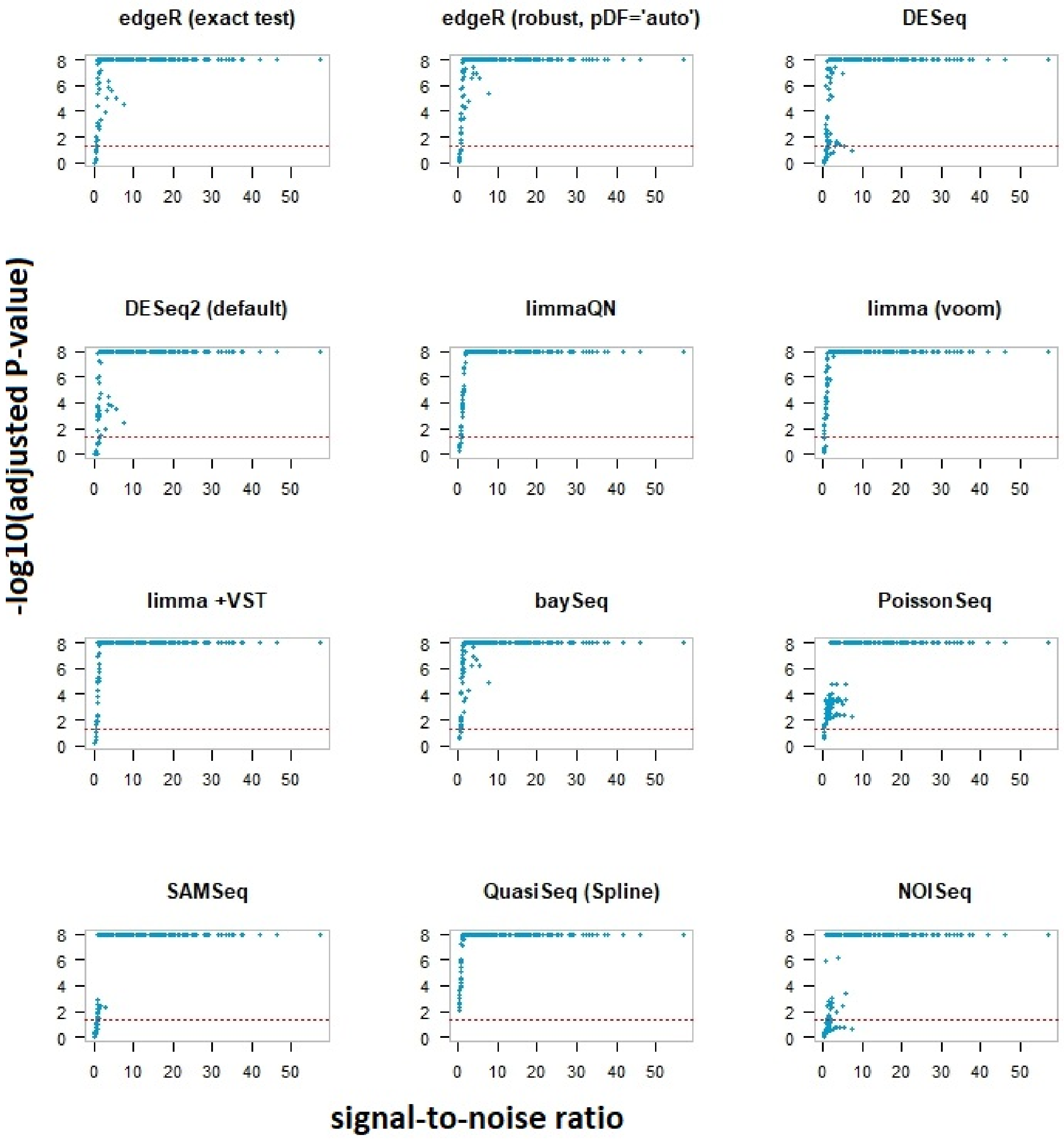

Figure S6: **Adjusted p-value versus signal-to-noise ratio for genes detected only in one group:** The red dashed line indicates the $-\log_{10}(0.05)$.

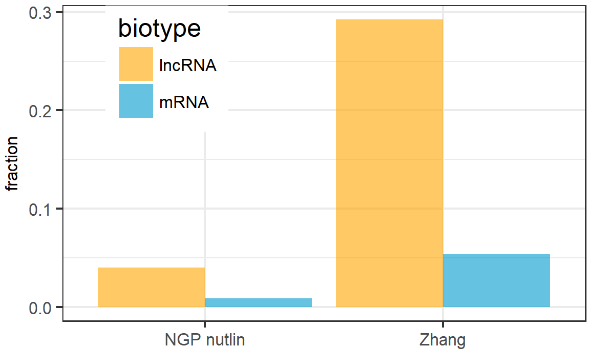

Figure S7: The fraction of mRNAs and lncRNAs excluded by the independent filtering feature in DESeq2 pipeline. The result is summarized for the NGP nutlin and Zhang data.

3.3 Computation time

We also looked at the computation times as a function of number of replicates. From the GTEx dataset, we randomly selected 2 to 25 replicates, without replacement, from each condition. We ran each tool on these datasets and computed the elapsed time from normalization to the final output without further post-processing of results. We ran the tools on R software (version 3.3.2) [11] installed on a machine with 8 logical processors (4 cores), 16GB RAM, and Windows 10 operating system. baySeq used 7 cores whereas all others were ran on a single core. The results (Figure S8) indicate that baySeq requires the longest computation time that increases fast with increasing number of replicates. DESeq was second slowest. The other tools required less time (less than 2 minutes). The limma tools were the fastest, followed by PoissonSeq and the edgeR tools (except edgeR robust); they completed the task within seconds with quite stable run time for increasing number of replicates.

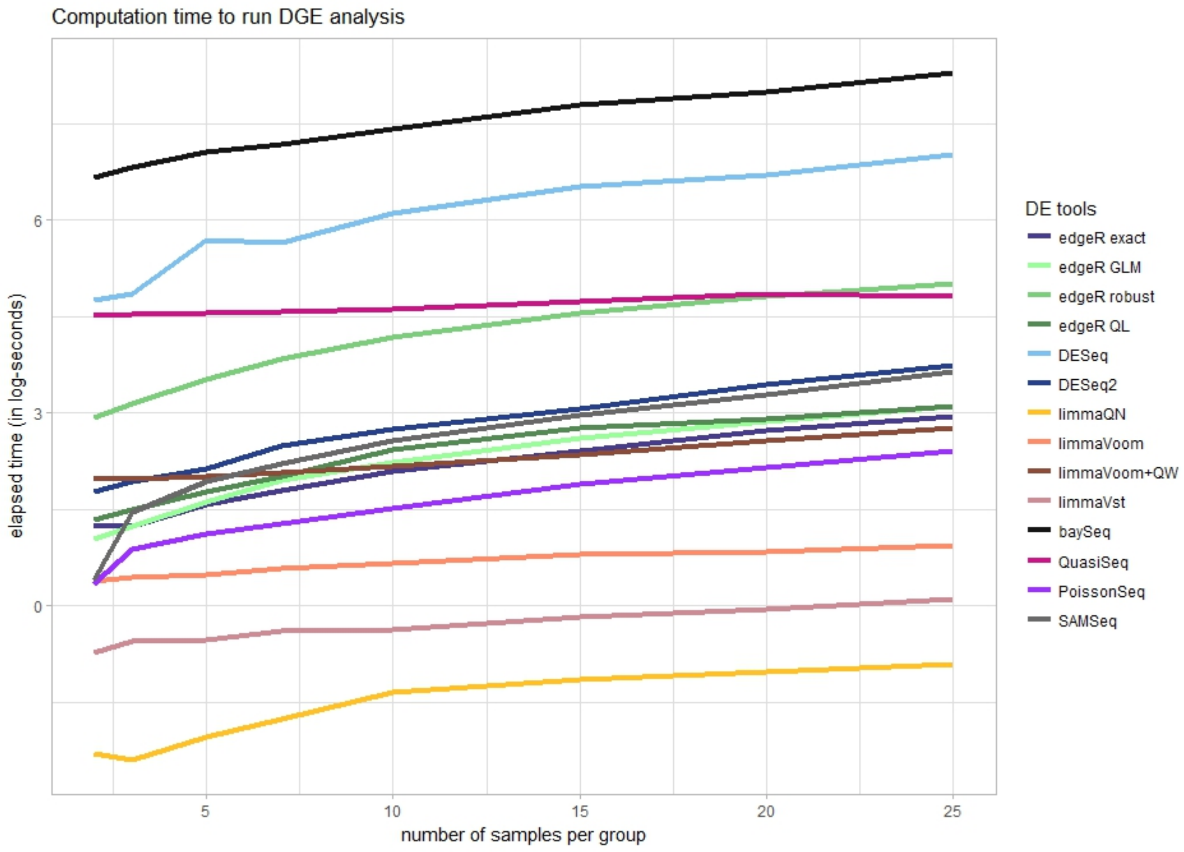

Figure S8: **Computation time:** Computation time of 14 DE tools for DGE analysis as a function of number of samples per group. The time is in log of seconds. limma tools often run DGE analysis faster, even with higher number of replicates. PoissonSeq and edgeR tools rank second best. baySeq takes the longest time to run DGE analysis that escalates to hours for higher number of sample replicates. DESeq, QuasiSeq, and edgeR robust also take at least 15 minutes.

4 Simulation study

4.1 SimSeq simulation procedures

Let us denote the source dataset by a $G \times n$ matrix $\mathbf{Y}$, with G rows for genes and n columns for samples with $n = n_1 + n_2$, where n_k is the number of samples from the kth group, $k \in (1, 2)$. Let $\mathbf{c}$ be the vector of multiplicative normalization factors with dimension $n \times 1$, which are computed using TMM normalization method with *calcNormFactors* function from the *edgeR* package. Note that G and n are the set of all genes and samples in $\mathbf{Y}$, respectively. The genes in the source dataset are expressed at least in one of the samples from the two groups.

First a set of DE genes, containing m_1 genes, (denoted as G_1 from the algorithm in [12]) is formulated by probability sampling without replacement with weight vector $\mathbf{w}^t = (w_1\ w_2\ ...\ w_G)$. Elements of the weight vector w_i is the weight to the ith gene, i = 1, 2, ..G, and $w_i = 1 - P_i^*$, where P_i^* is the local false discovery rate calculated using *fdrtool* package [13] based on the G P-values from Wilcoxon Rank Sum test applied to the source dataset $\mathbf{Y}$ with a vector of normalization factor $\mathbf{c}$ using *CalcPvalWilcox* function in *SimSeq* package as well. Therefore, during the probability sampling, priority is given to genes with higher evidence of differential expression from the source dataset (low p value yielding higher weight). Then, the selected genes designated as truly DE genes.

Second, a set of equivalently expressed (EE) genes, containing m_0 genes, (denoted as G_0 from the algorithm in [12]) is formulated by equal probability sampling without replacement from a set of genes that are not included in DE set of genes.

Therefore, $G' = m_0 + m_1 \leq G$ genes will be selected from the source dataset. For the selected G' genes, n' samples/replicates are sampled without replacement from the source count matrix $\mathbf{Y}$ in a fashion discussed clearly in the paper [12]. Finally, each simulated count matrix has a dimension of $G' \times n'$, where $n' \leq min\{n_1, \lfloor n_2/2 \rfloor\}$, where $\lfloor . \rfloor$ is the floor function.

Let p be the proportion of DE genes ($p = \frac{m_1}{G'}$) and $1 - p$ the proportion of EE genes. For the three simulation each starting from different distinct RNA-seq dataset, the values of G, G', n, n', and p are summarized as follows.

simulation number	source data	G	G'	n_k	n'_k	p
1	Zhang	29,305	10,000	172	2 - 40	0-30%
2	NGP nutlin	26,418	10,000	20	2 - 5	0-30%
3	GTEx	18,632	10,000	58	2- 14	0-30%

Each simulated count data from the first two simulation contain a number of mRNA and lncRNAs both in the set of DE genes and EE genes. The fractions are summarized and presented in the web tool (`http://statapps.ugent.be/tools/AppDGE/`) under the 'Features of simulated

data' panel (first click on a check box 'Show features of simulated gene expression data' from the first page to go to the desired panel).

4.2 Additional simulation results

4.2.1 False discovery and true positive rates

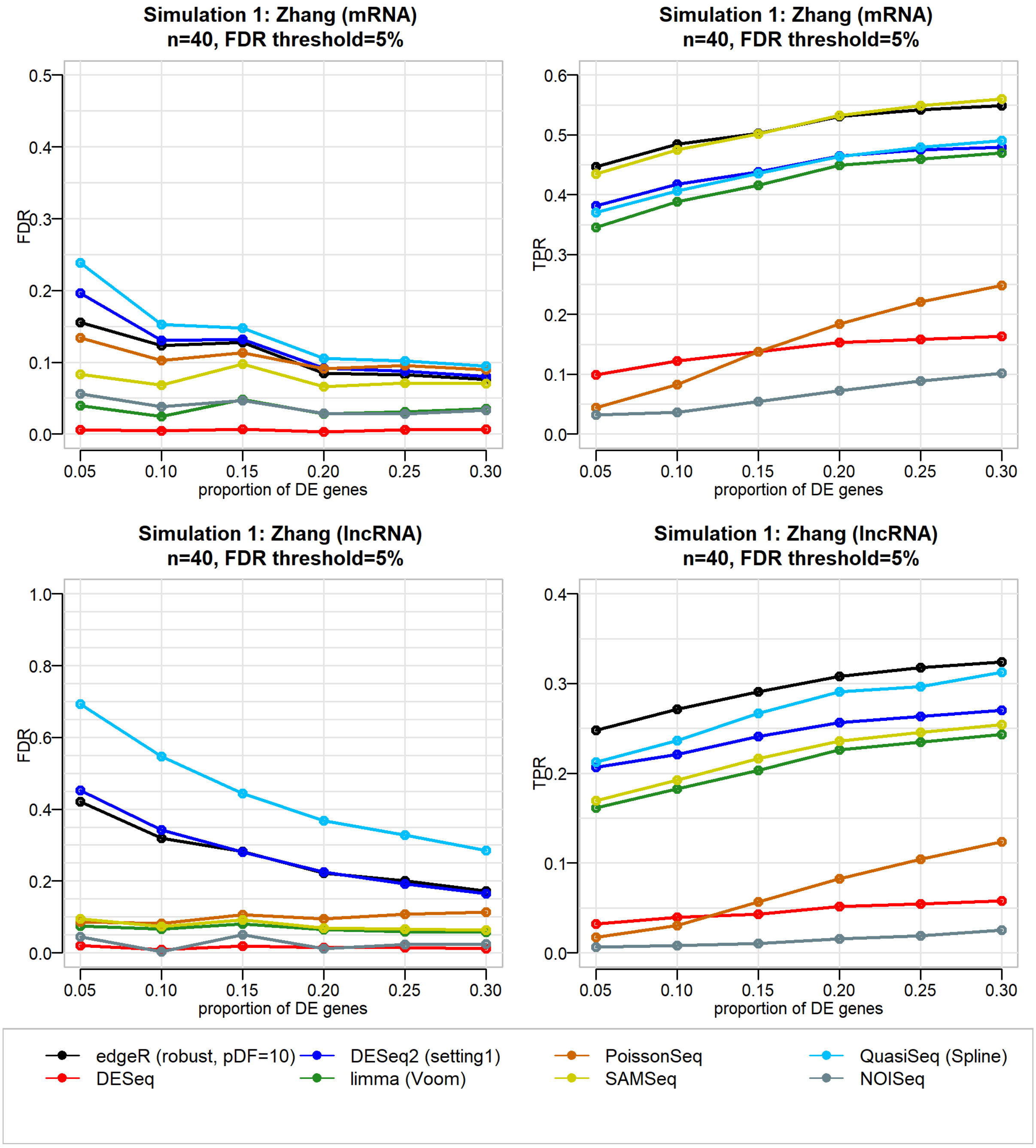

Figure S9: **Additional simulation results.** Performance (FDR and TPR) of 8 DE tools at different proportion of true DE genes (5-30%) in the simulated data. The result is from simulation 1 (with the Zhang data as a source data), 40 replicates per group,and 5% FDR threshold.

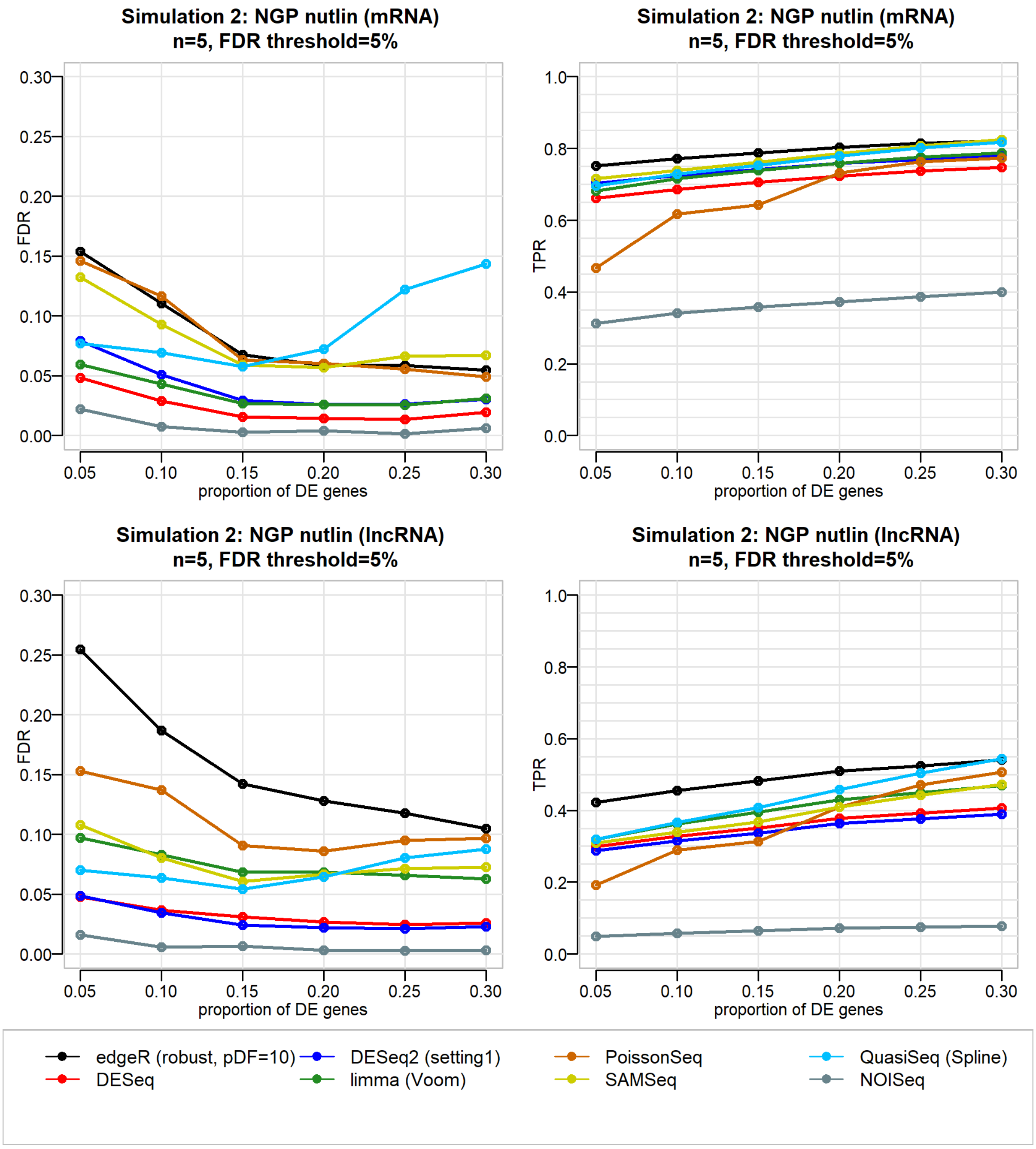

Figure S10: **Additional simulation results.** Performance (FDR and TPR) of 8 DE tools at different proportion of true DE genes (5-30%) in the simulated data. The result is from simulation 2 (with the NGP data as a source data), 5 replicates per group,and 5% FDR threshold.

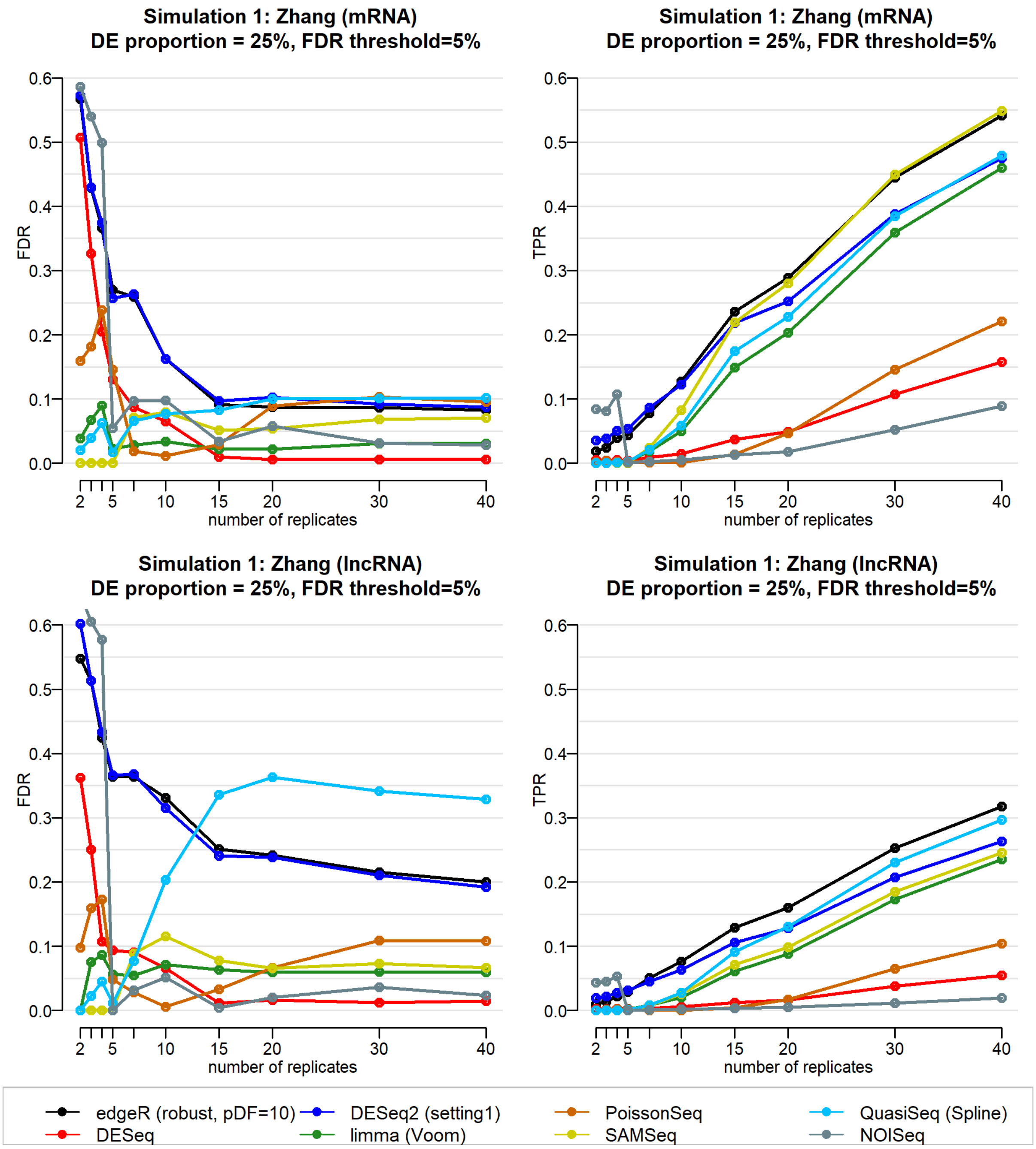

Figure S11: **Additional simulation results.** Performance (FDR and TPR) of 8 DE tools at different number of samples per group (2-40) in the simulated data. The result is from simulation 1 (with the Zhang data as a source data), 25% true DE genes, and 5% FDR threshold.

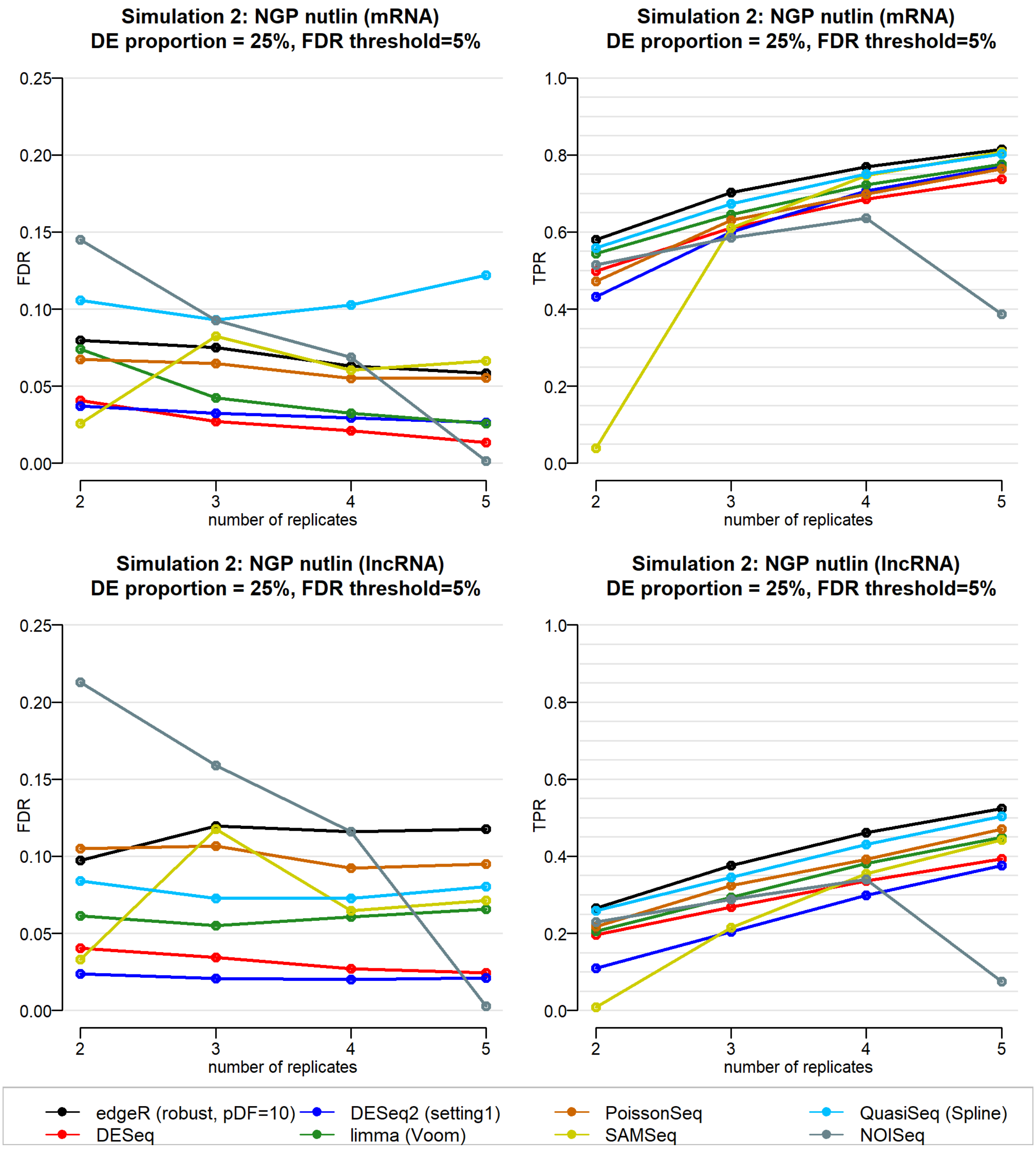

Figure S12: **Additional simulation results.** Performance (FDR and TPR) of 8 DE tools at different number of samples per group (2-5) in the simulated data. The result is from simulation 2 (with the NGP nutlin data as a source data), 25% true DE genes, and 5% FDR threshold.

4.2.2 Performance of DE tools for low count mRNAs

Up to this point, we have demonstrated that DE tools have inferior performance for lncRNAs compare to mRNAs. In this section, we explore the performance of DE tools for low-count mRNAs by partitioning simulated mRNA expression data (starting from the GTEx data) into two groups that reflect different level of abundance using the following procedures. First, mRNA genes are grouped into two non-overlapping classes in each simulated data: low and high-count genes. To create the groups, the count matrix is first converted to counts-per-millions (CPM) using *cpm()* function in *edgeR* package [7]. The CPM calculation uses the effective (normalized) library sizes obtained using TMM normalization method [14]. Afterwards, gene-specific average CPM values are calculated as the arithmetic mean of CPM across all samples. Genes with average CPM less than 10 were grouped together and designated as low-count genes (accounting 35% on average), whereas genes with at least 10 average CPM were grouped together and designated as high-count genes (accounting 65% on average). The cut point 10 CPM was chosen based on the evidence that from the NGP nutlin and Zhang datasets 99% of lncRNAs have average CPM less than 20 for average library size of ≈15.8 million, which is approximately equivalent to 10 CPM for 10 000 mRNAs with average library size ≈8.9 million. In both groups the proportion of true DE genes is approximately 20%. Results from performance evaluation are summarized in Figure S13. Like lncRNAs, the actual FDR for low-count mRNA genes was larger than that of high-count mRNA genes. Also, the sensitivity of DE tools was slightly lower for low-count mRNAs than for high-count mRNAs.

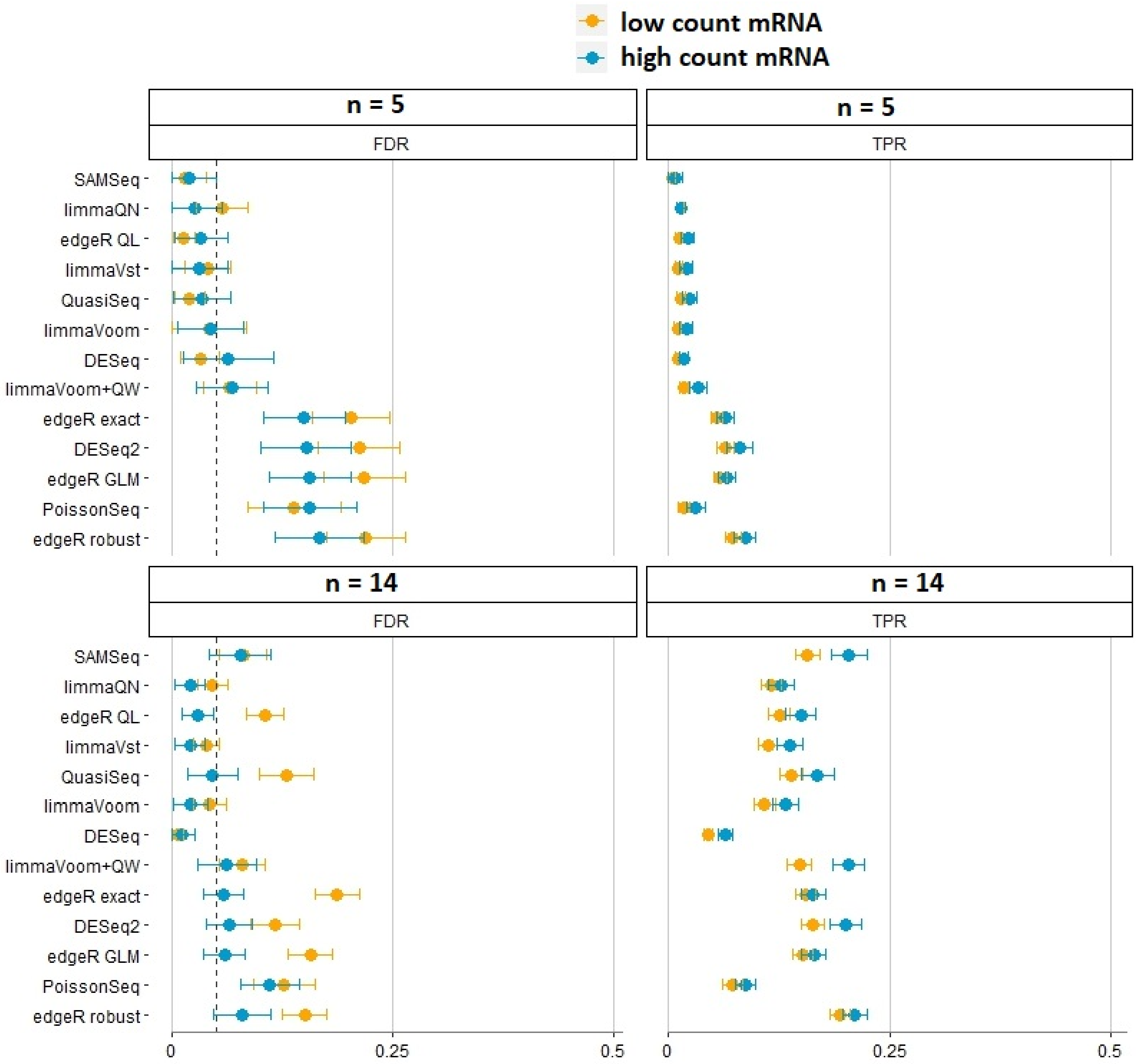

Figure S13: FDR and TPR of 13 DE pipelines for low and high-count mRNAs. The simulation settings includes 20% proportion of true DE genes and n=5 and 14 replicates per group. The simulation started from the GTEx data. All simulated genes are mRNAs, with N=10,000.

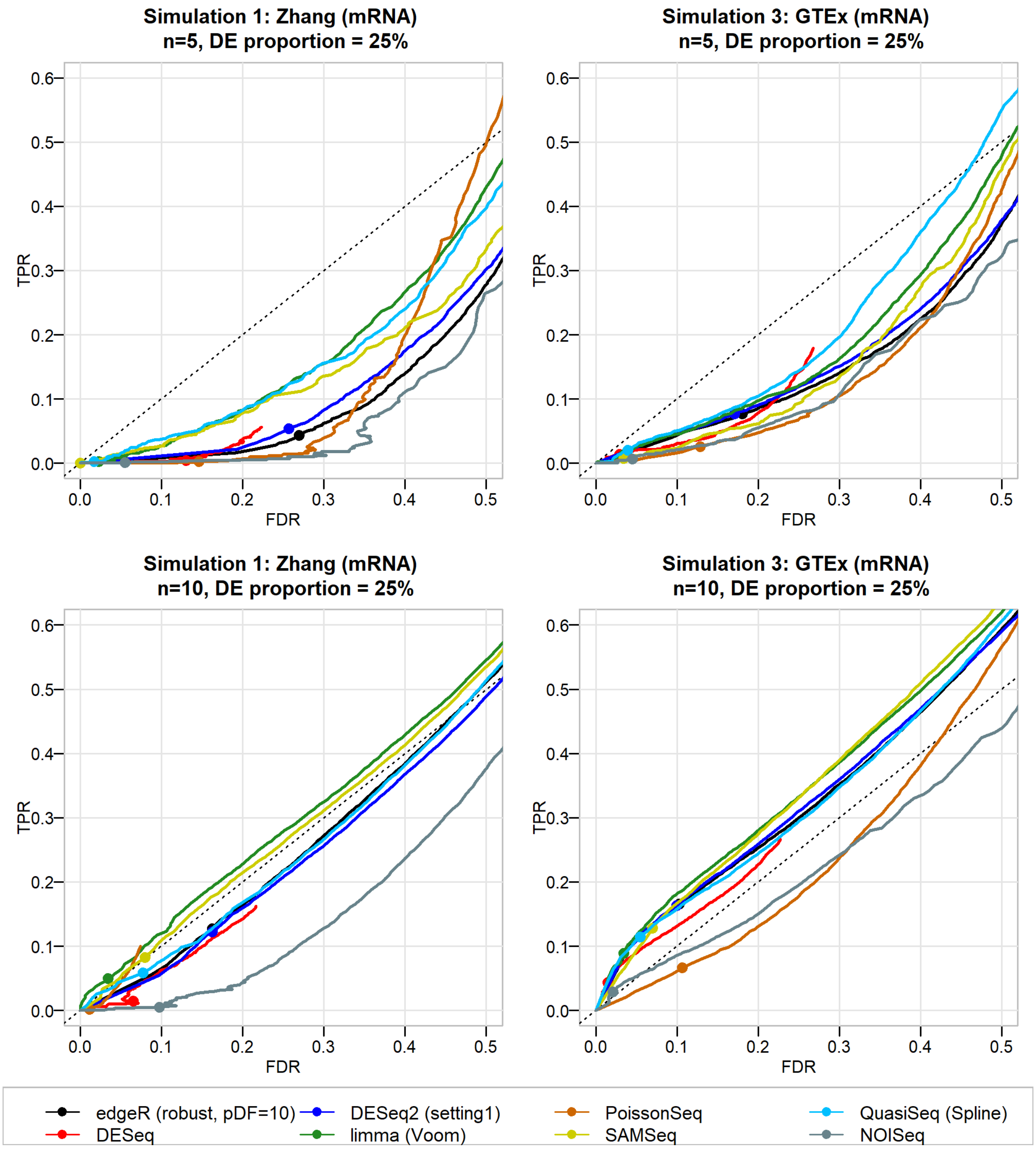

Figure S14: **Additional simulation results.** Performance (FDR and TPR) of 8 DE tools from simulation 1 (Zhang) and 3 (GTEx). The result is for simulated data with 25% true DE genes, only mRNA genes, and 5 and 10 replicates per group.

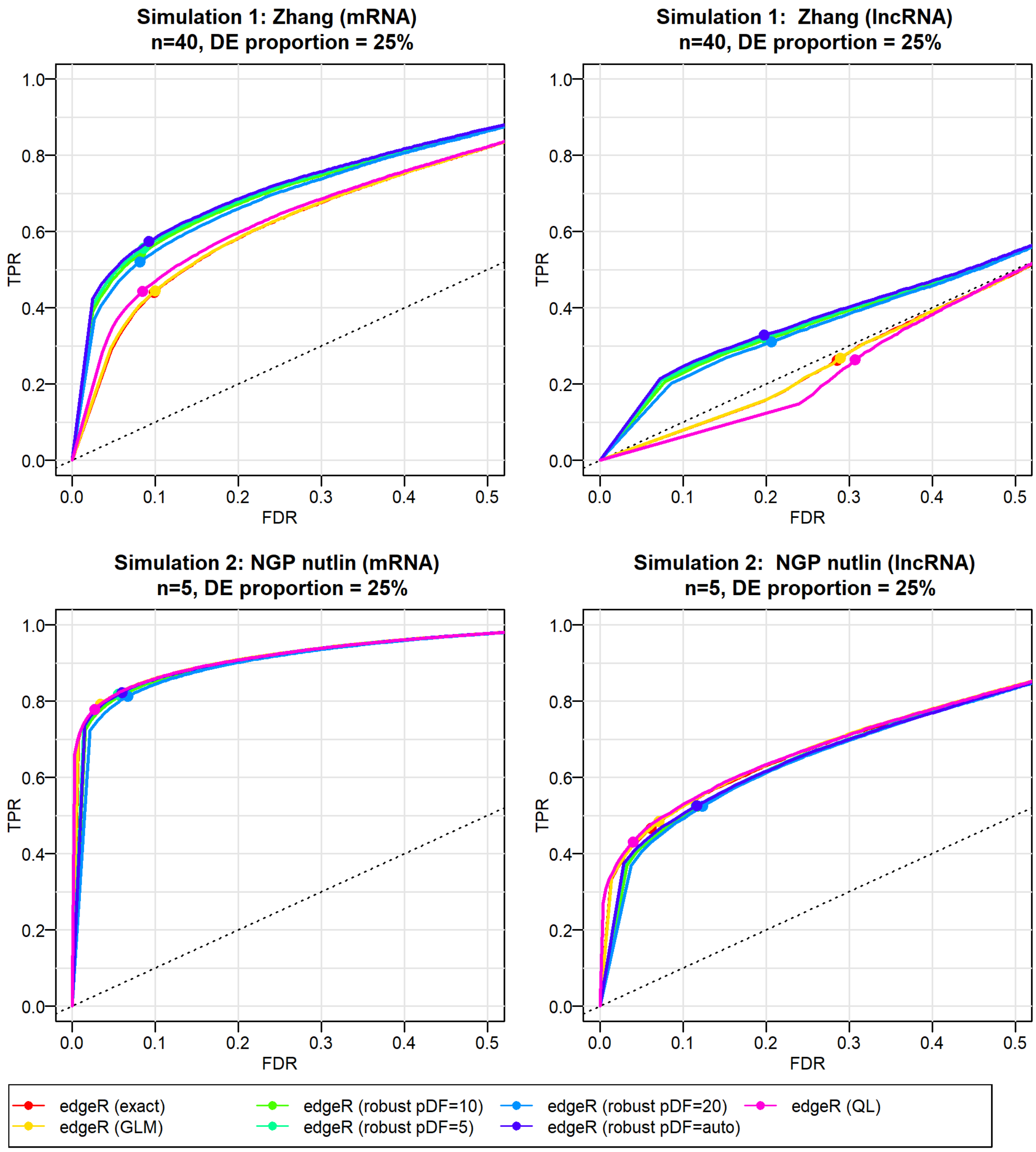

Figure S15: **Additional simulation results.** Performance (FDR and TPR) of the 7 edgeR pipelines for a simulated data with 25% true DE genes. The result is from simulation 1 and 2, with 40 and 5 replicates per group, respectively.

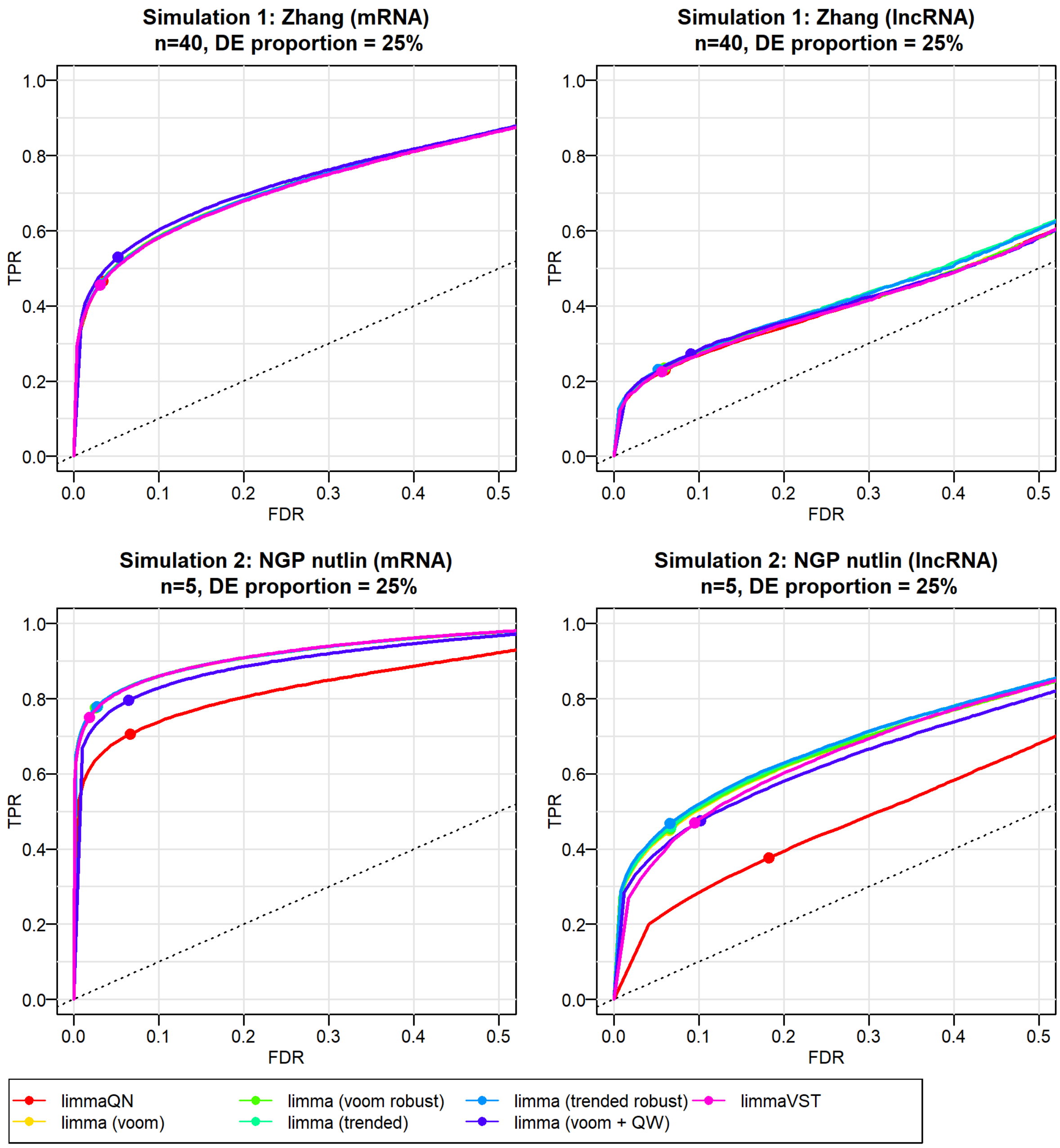

Figure S16: **Additional simulation results.** Performance (FDR and TPR) of the 7 limma pipelines for a simulated data with 25% true DE genes. The results are from simulation 1 and 2, with 40 and 5 replicates per group, respectively.

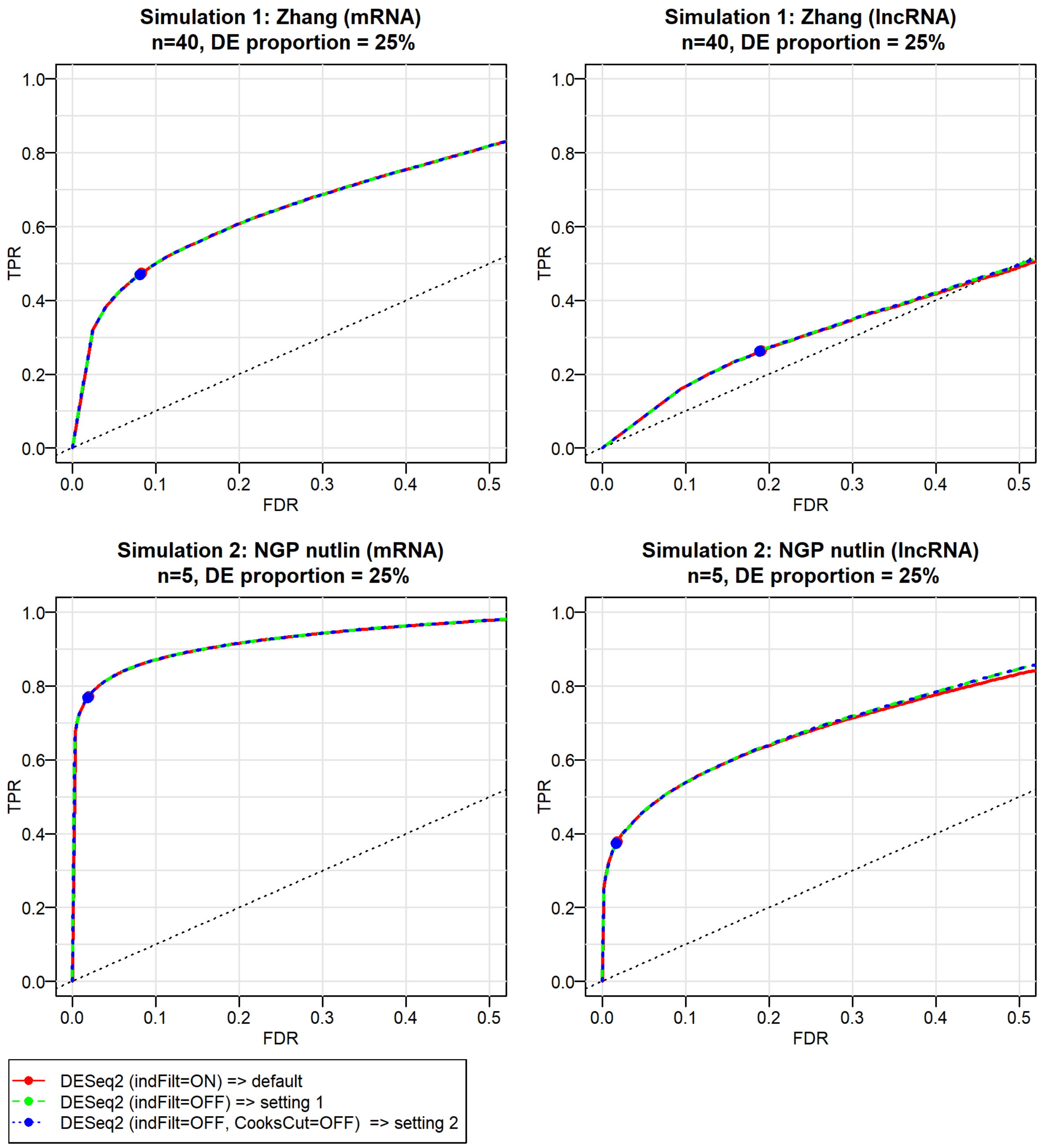

Figure S17: **Additional simulation results.** Performance (FDR and TPR) of the 3 DESeq2 pipelines for a simulated data with 25% true DE genes. The results are from simulation 1 and 2, with 40 and 5 replicates per group, respectively.

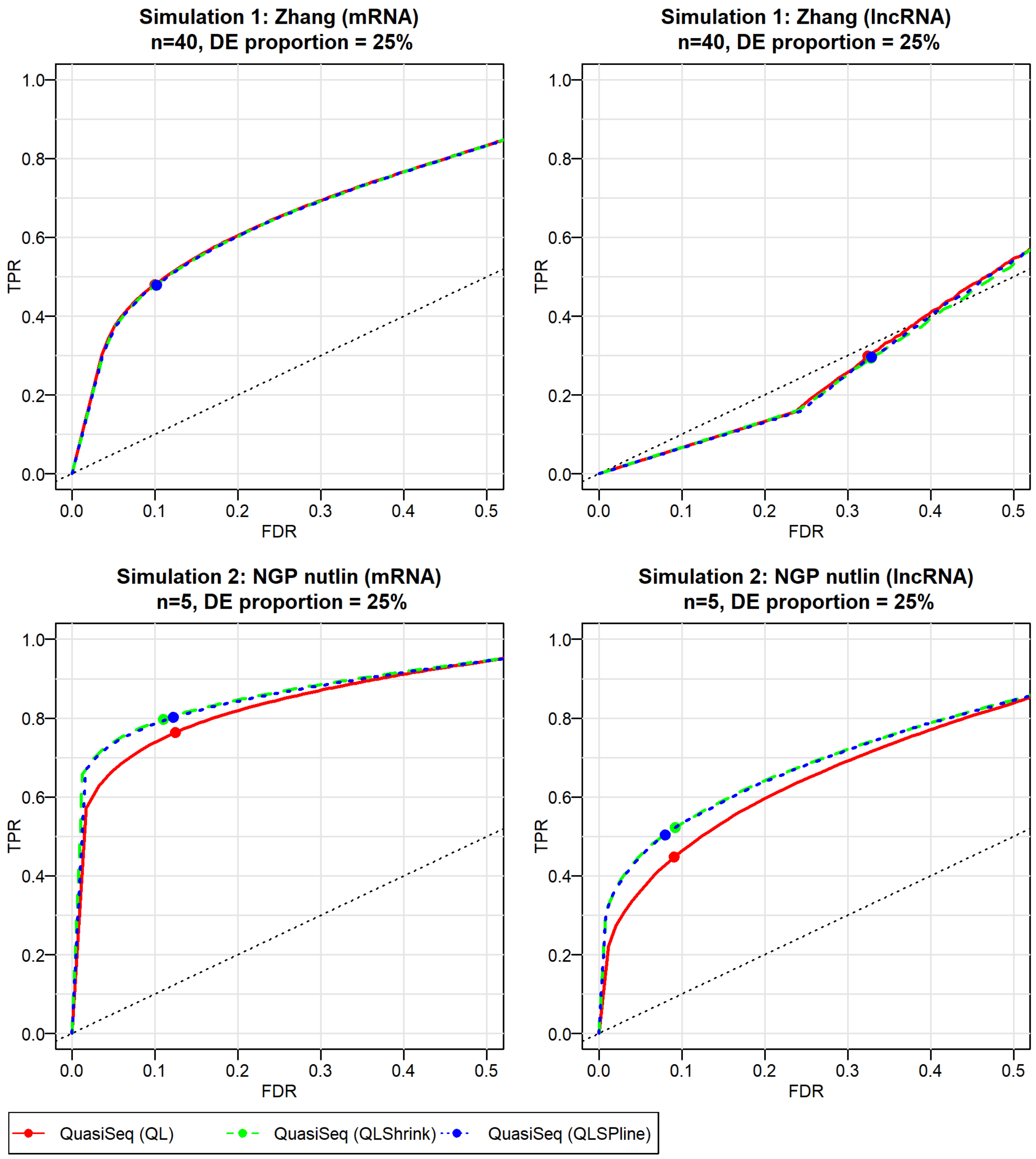

Figure S18: **Additional simulation results.** Performance (FDR and TPR) of the 3 QuasiSeq pipelines for a simulated data with 25% true DE genes. The results are from simulation 1 and 2, with 40 and 5 replicates per group, respectively.

4.2.3 Sample filtering

We ran an extra simulation that starts from the Zhang data (which contains the most variable samples) by filtering a set of outlying samples beforehand. First, principal component analysis (PCA) was done on the full normalized Zhang data (n_1 = 81 and n_2=91) by excluding genes

with zero expression. The PCA was done using *plotPCA* function under DESeq2 bioconductor package based on variance-stabilizing-transformation of the read counts. The first two principal components (PC1 and PC2) that explain 16% and 9% of the total variability, respectively, were used to visualize samples in a two-dimensional space (Figure S19-panel A). Samples within the top and bottom 10% of the two PCs (37 samples out of 172) were excluded as these sample are furthest from the rest of the samples. The extent of biological variability was relatively lower in the filtered Zhang data than that of the original Zhang data (Figure S19-panel B). The remaining set of samples were subsequently used as a new source data to run SimSeq simulation. In particular, 100 RNA-seq data each with 20 samples per group, 10,000 genes (70% mRNA and 30% lncRNA) and 25% DE genes were simulated. The performance of DE tools (with respect to FDR and TPR) from the new simulation procedure is presented in Figure S20. Readers should compare this result with the plot in Figure 4 (the top 2 panels) of the main manuscript.

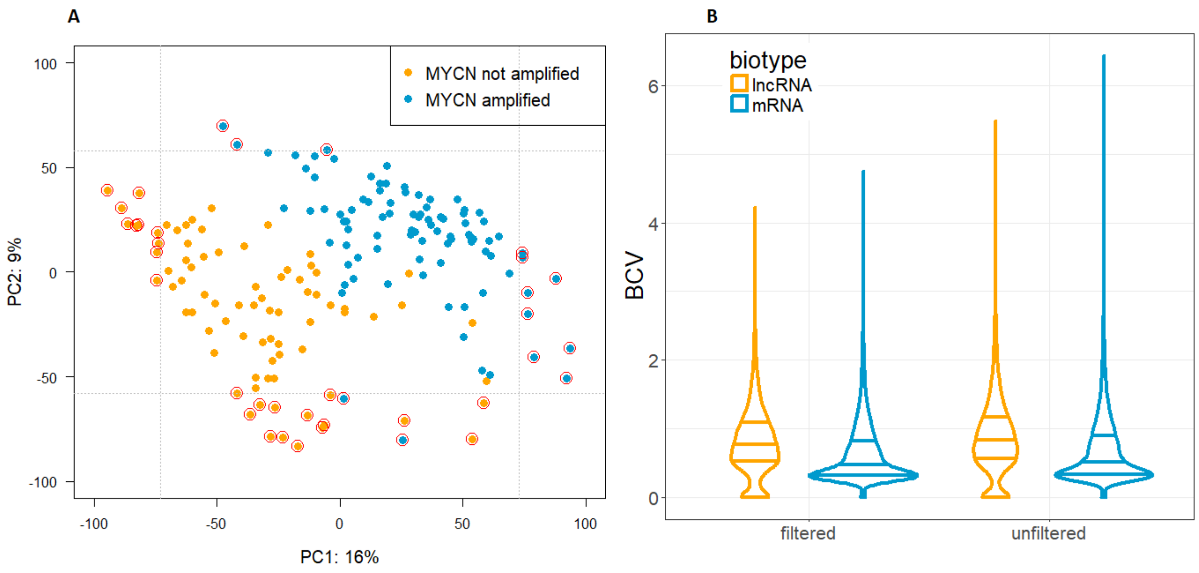

Figure S19: **Sample filtering and intra-group variability.** A-The first two PCs that jointly explain 25% of the total variability. Each dot represents a sample from the Zhang data and dots enclosed in a red circle indicate samples that have the top and bottom 10% of PC1 and PC2 (indicated by the grey dashed lines). B-The estimated biological coefficients of variation (BCV) for the filtered and unfiltered Zhang data. The BCVs are separately shown for mRNA and lncRNAs

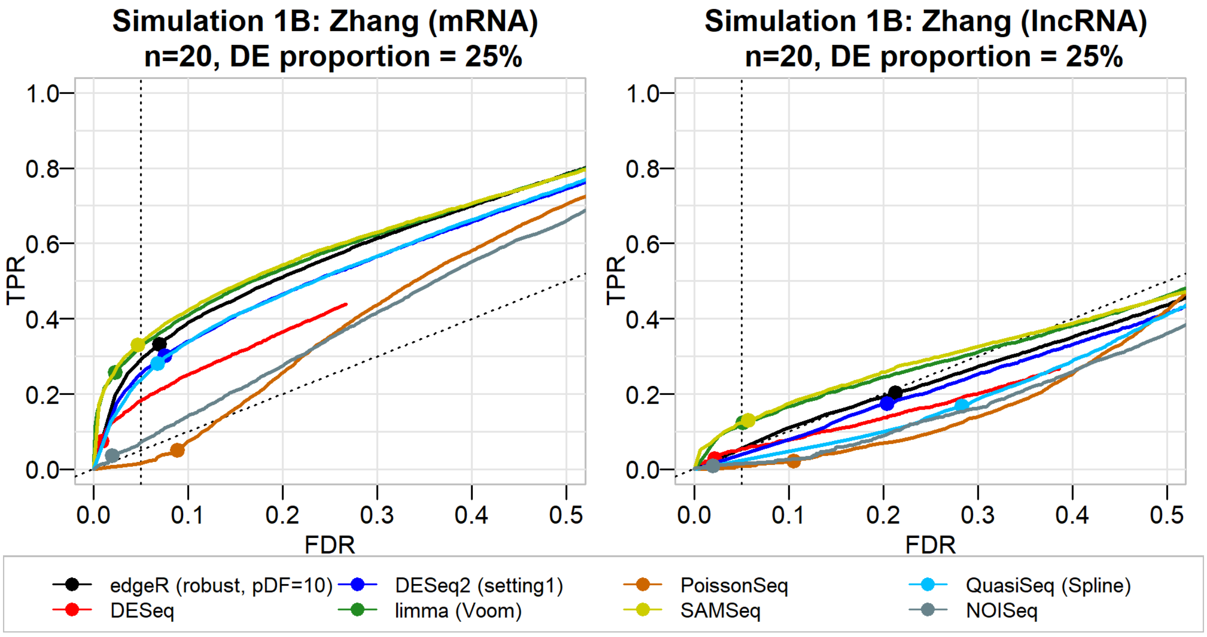

Figure S20: **Additional simulation results.** Performance (FDR and TPR) of DE tools for a simulated data with 25% true DE genes. The results are from simulation that started from a filtered Zhang data. The simulated data includes 20 samples per group and 25% DE genes.

4.2.4 Distribution of P values

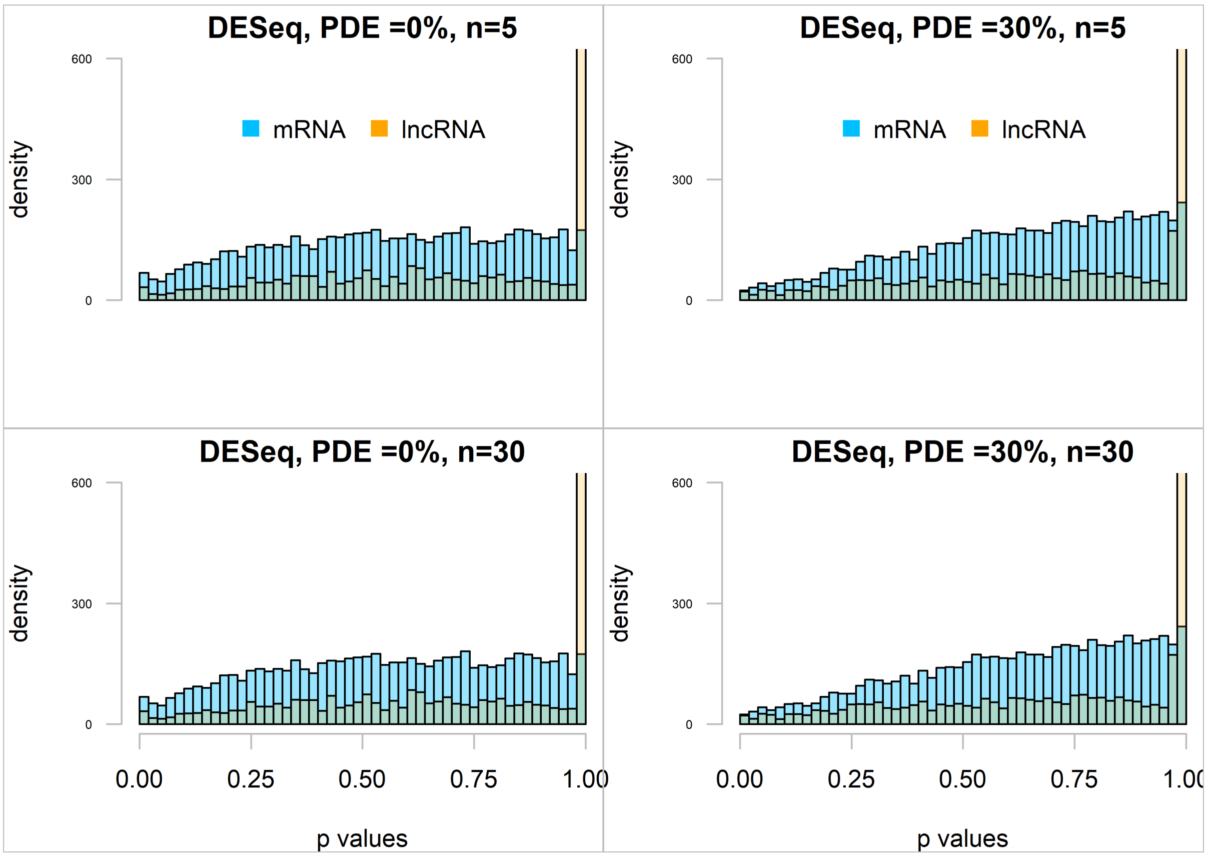

Figure S21: Distribution of p-values. The result is shown for a randomly selected simulation that starts from the Zhang data.

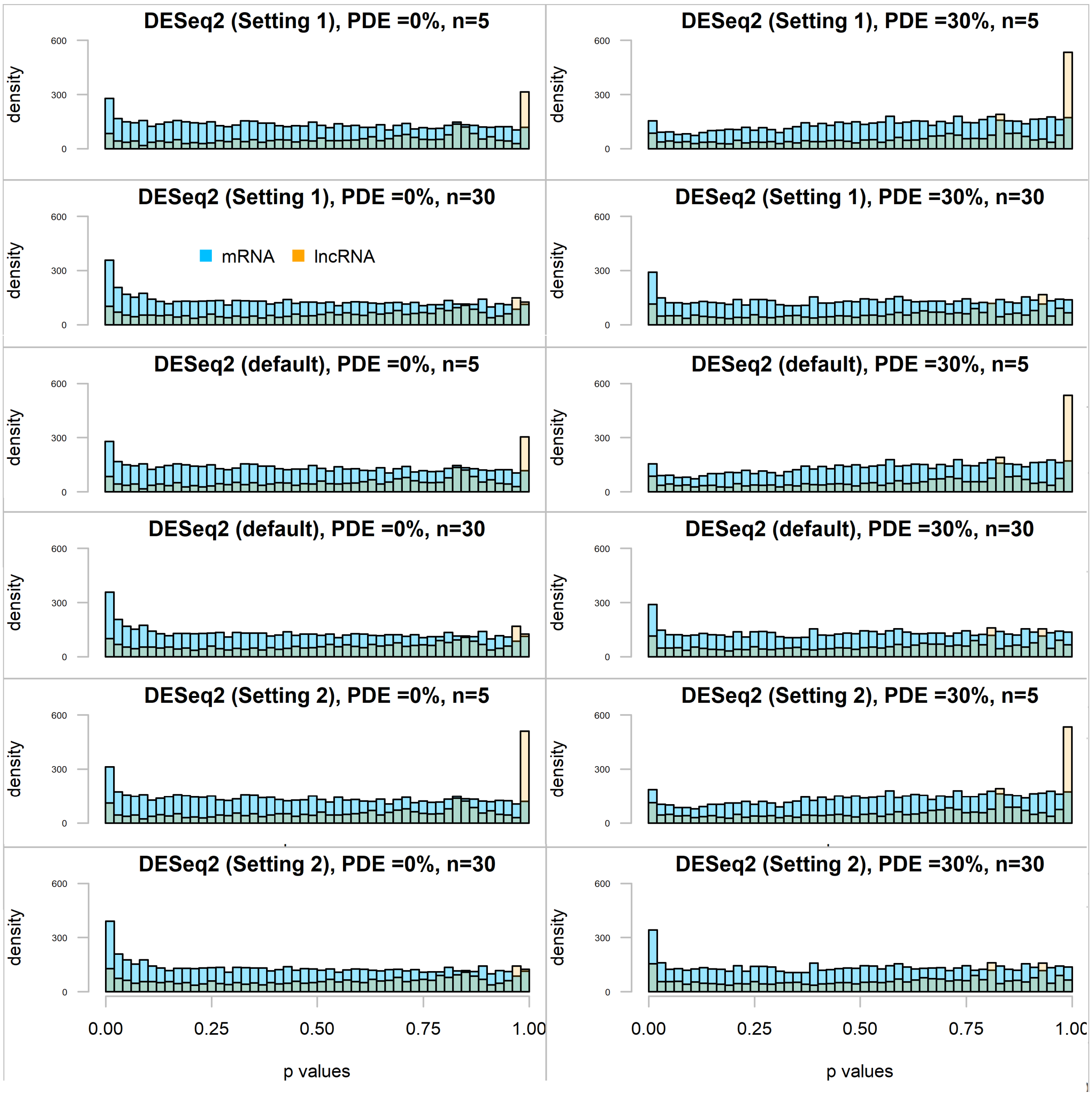

Figure S22: Distribution of p-values. The result is shown for a randomly selected simulation that starts from the Zhang data.

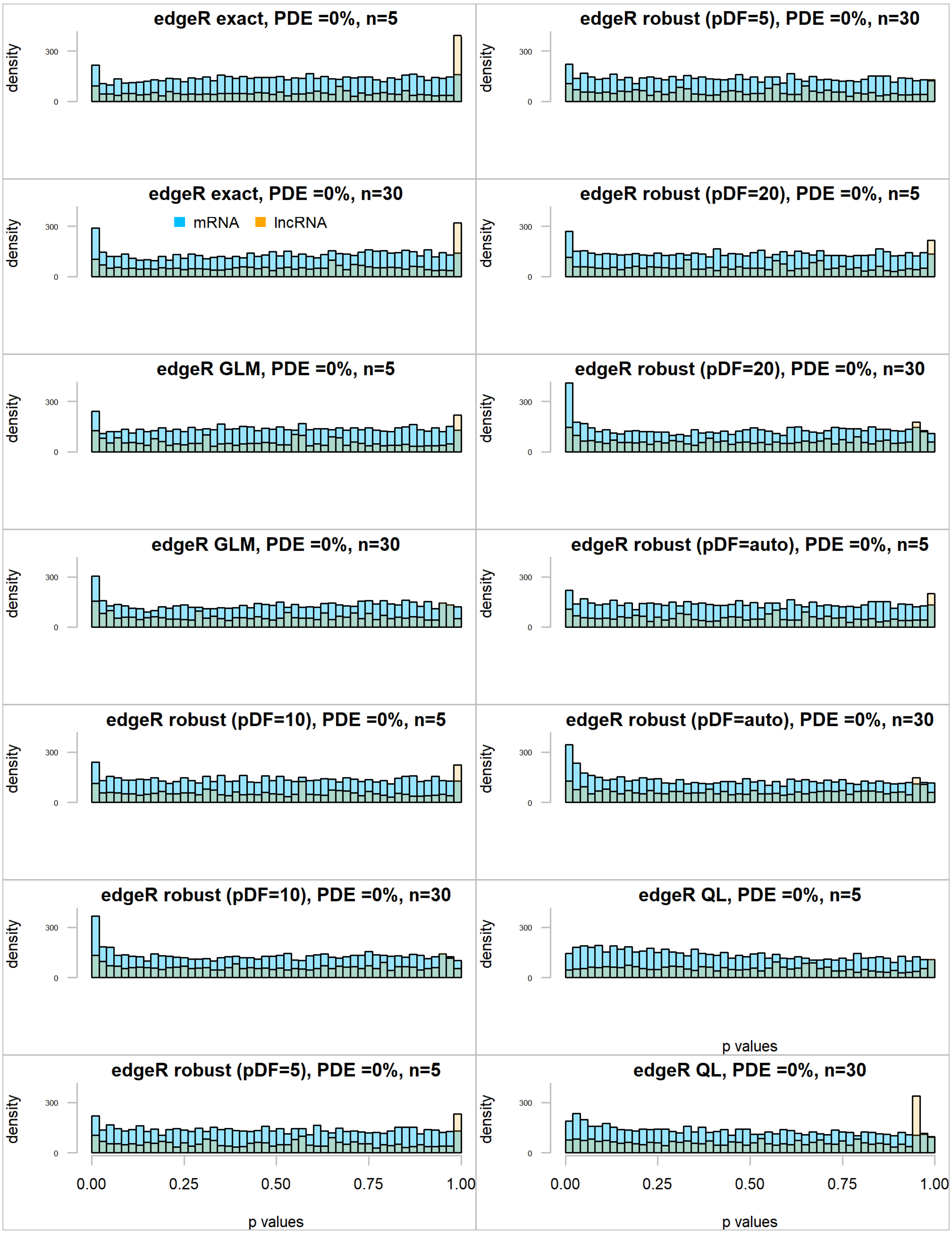

Figure S23: Distribution of p-values. The result is shown for a randomly selected simulation that starts from the Zhang data.

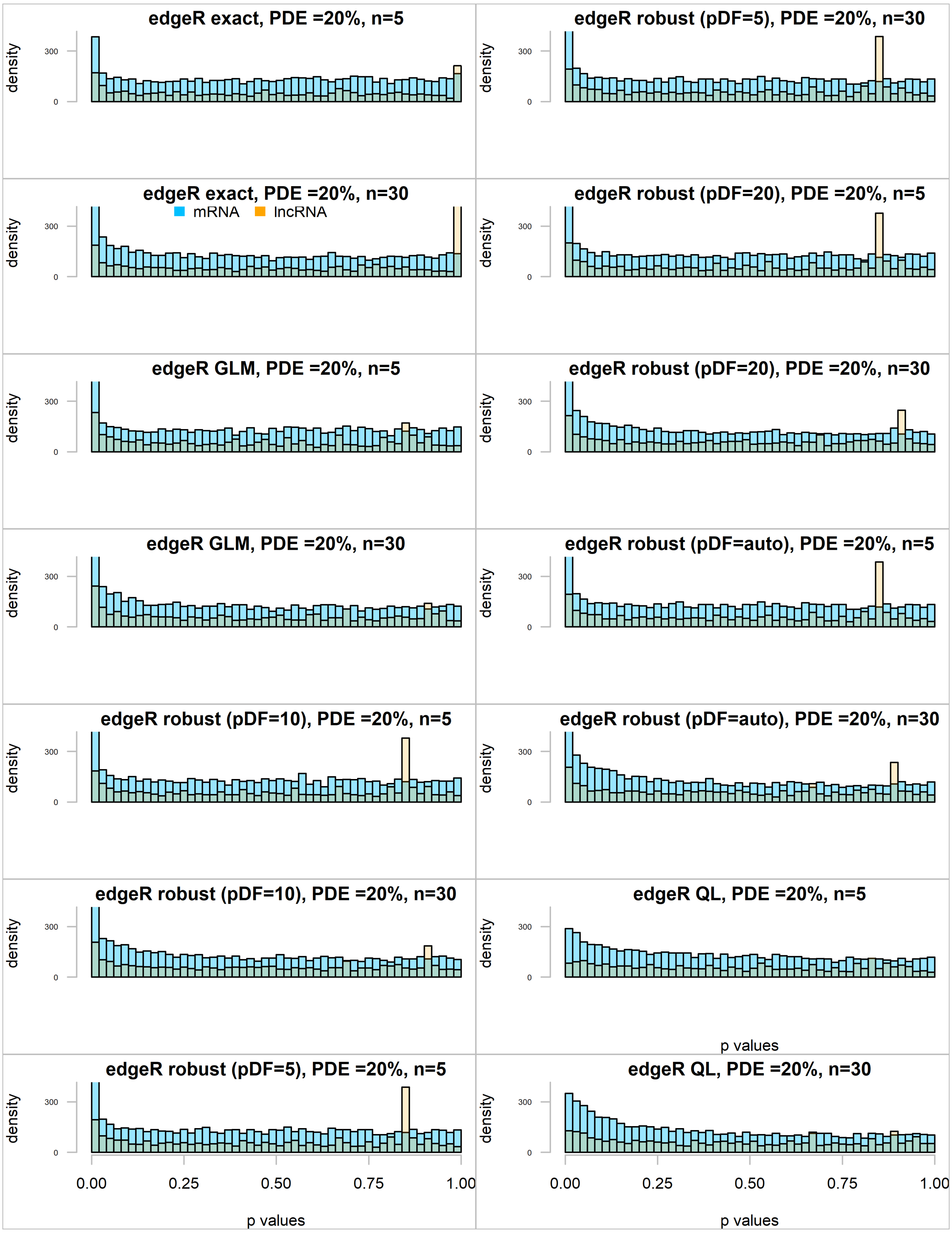

Figure S24: Distribution of p-values. The result is shown for a randomly selected simulation that starts from the Zhang data.

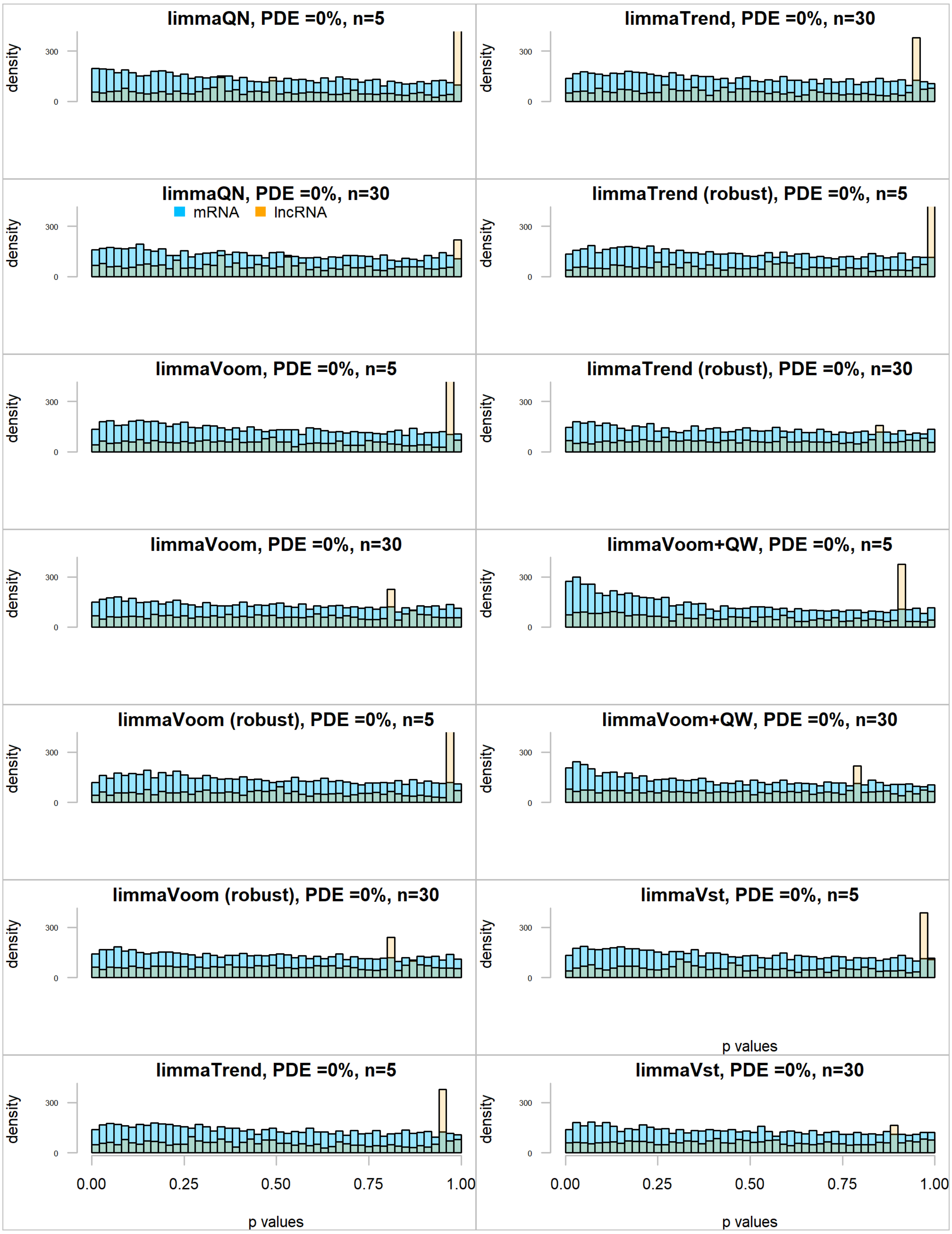

Figure S25: Distribution of p-values. The result is shown for a randomly selected simulation that starts from the Zhang data.

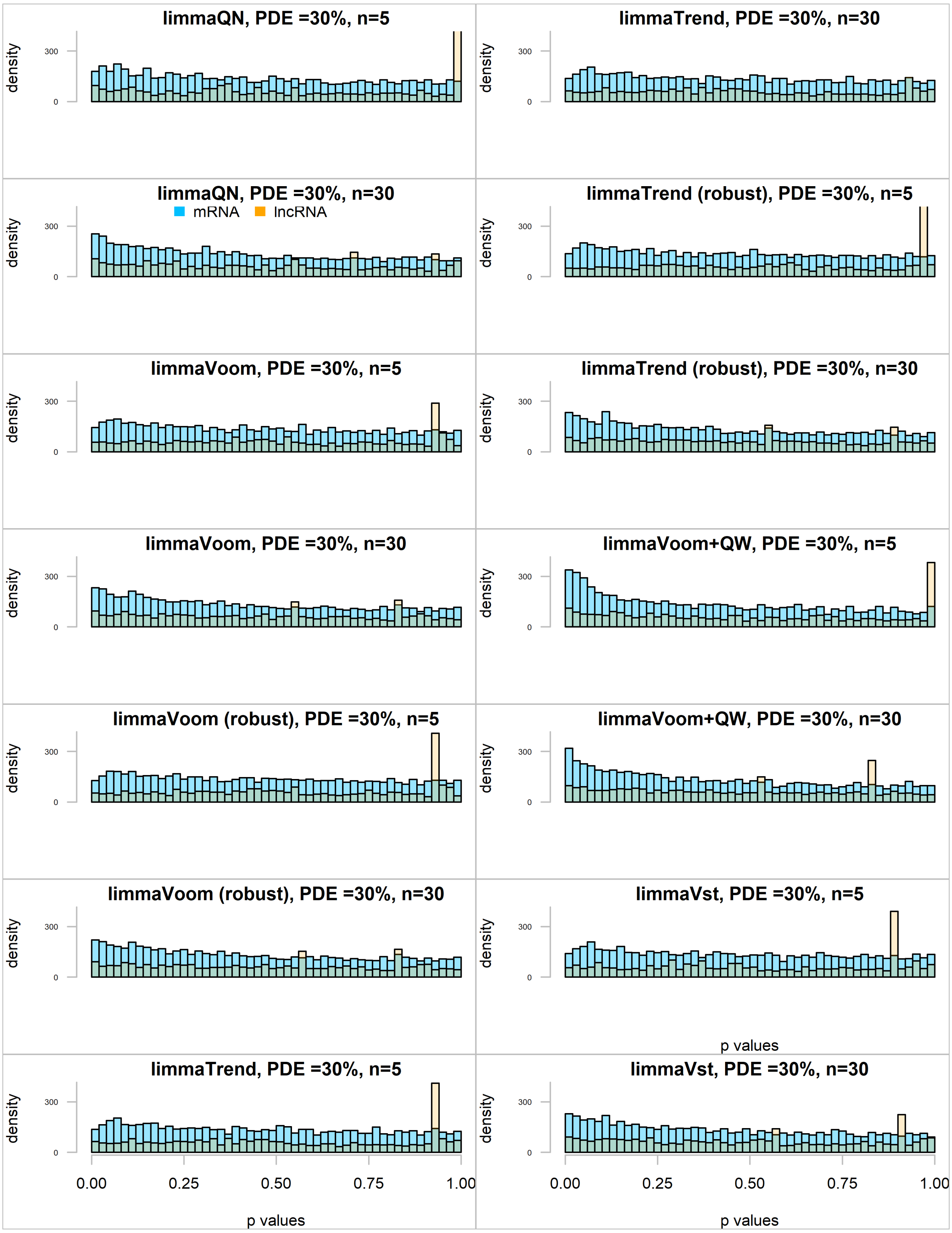

Figure S26: Distribution of p-values. The result is shown for a randomly selected simulation that starts from the Zhang data.

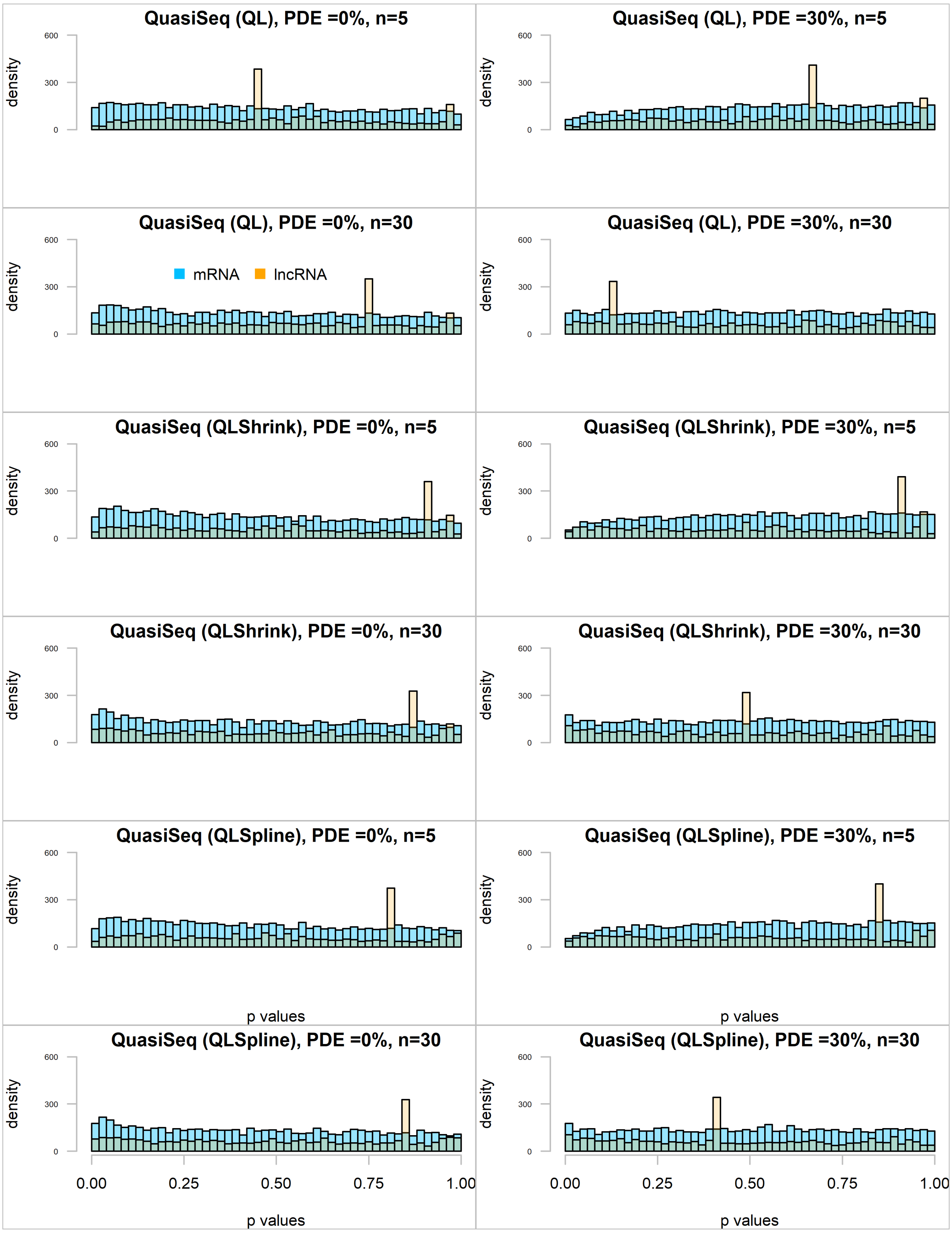

Figure S27: Distribution of p-values. The result is shown for a randomly selected simulation that starts from the Zhang data.

4.2.5 False positive rates

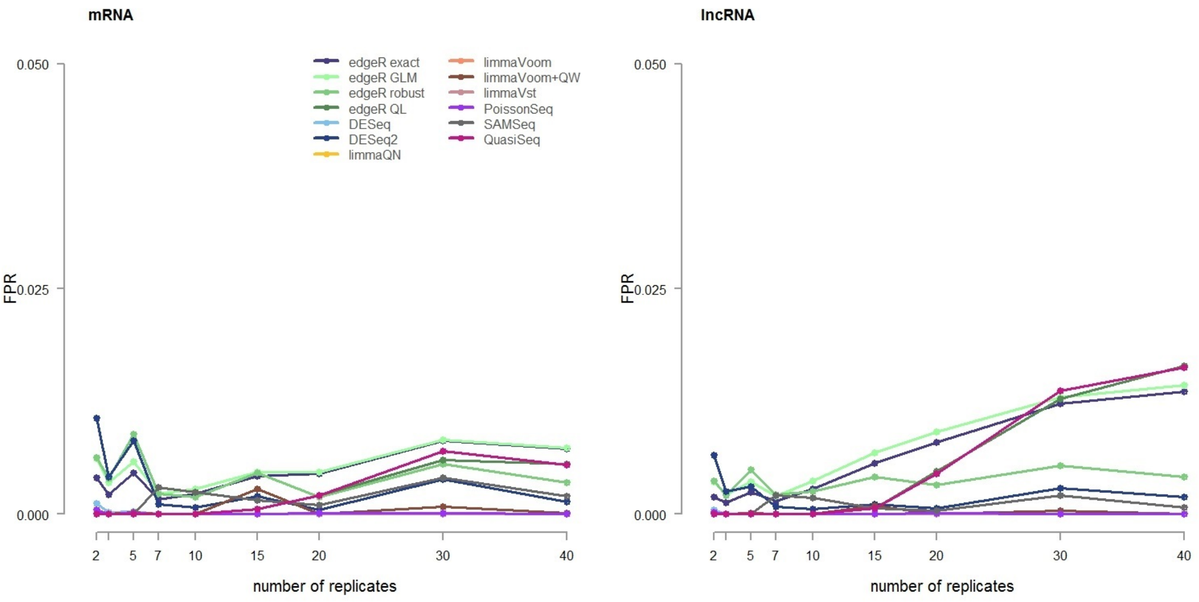

Figure S28: **Proportion of false positives.** It shows the average false positive rate of 13 DE tools. The average was calculated from 100 independent simulations each of with 0% true DE genes. The results are summarized for different number of replicates (2-40) and separately for mRNAs and lncRNAs.

4.2.6 Separate versus joint analysis of lncRNA and mRNA

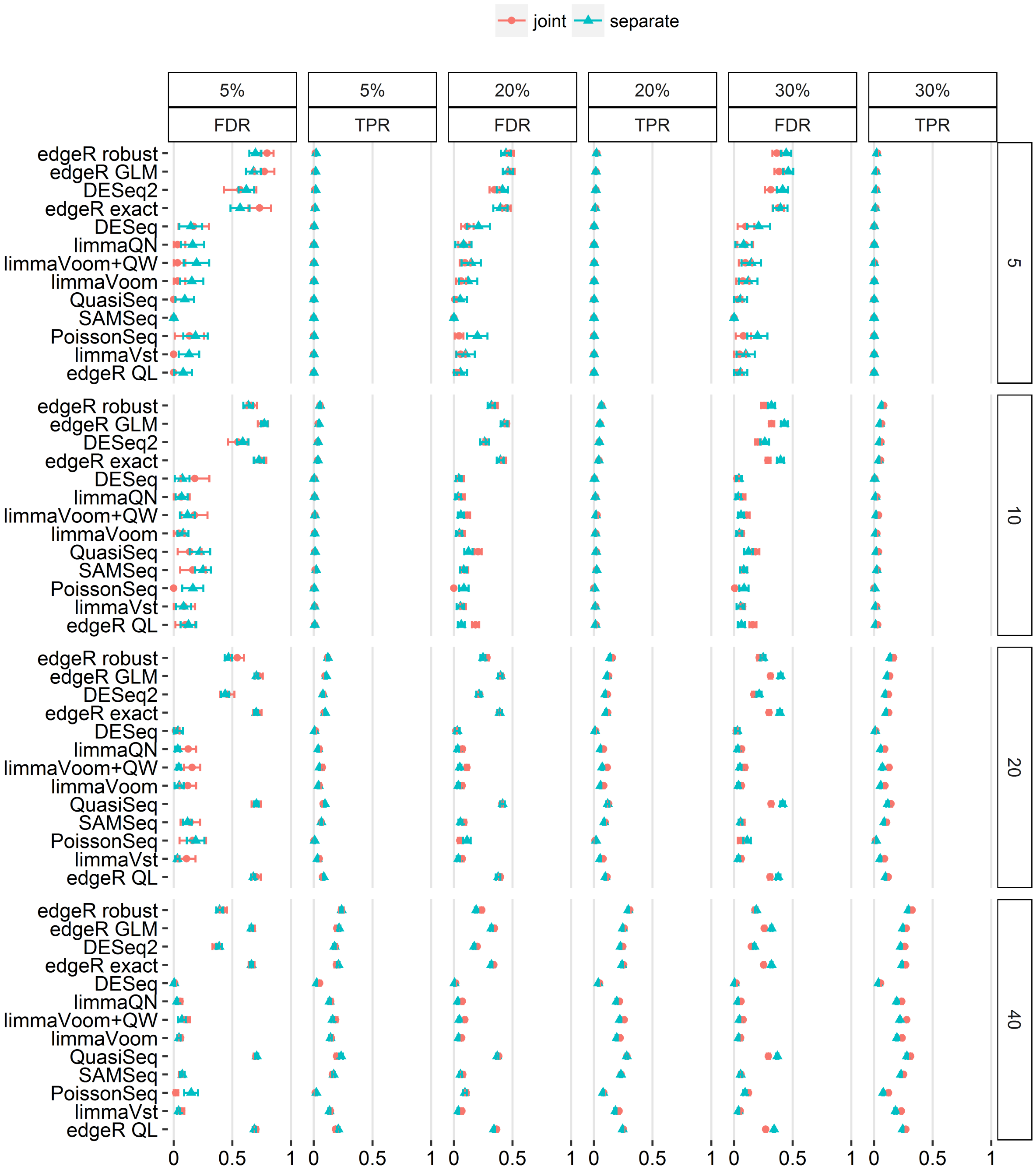

Figure S29: FDR and TPR of 13 DE pipelines for lncRNA from joint simulation with mRNA and separate simulation of lncRNAs (avoiding mRNAs first). It shows results for simulations with 5%, 20% and 30% proportion of true DE genes and n=5, 10, 20, and 40 replicates per group. The dots represent average results (FDR/TPR) calculated over 100 independent simulations. The error bars indicate the 95% interval estimate.

References

1. Rapaport, F. *et al.* Comprehensive evaluation of differential gene expression analysis methods for RNA-seq data. *Genome biology* **14,** 1 (2013).
2. Soneson, C. & Delorenzi, M. A comparison of methods for differential expression analysis of RNA-seq data. *BMC bioinformatics* **14,** 1 (2013).
3. Seyednasrollah, F., Laiho, A. & Elo, L. L. Comparison of software packages for detecting differential expression in RNA-seq studies. *Briefings in bioinformatics* **16,** 59–70 (2015).
4. Li, J., Witten, D. M., Johnstone, I. M. & Tibshirani, R. Normalization, testing, and false discovery rate estimation for RNA-sequencing data. *Biostatistics,* kxr031 (2011).
5. Schurch, N. J. *et al.* How many biological replicates are needed in an RNA-seq experiment and which differential expression tool should you use? *RNA* (2016).
6. Sahraeian, S. M. E. *et al.* Gaining comprehensive biological insight into the transcriptome by performing a broad-spectrum RNA-seq analysis. *Nature Communications* **8** (2017).
7. Robinson, M. D., McCarthy, D. J. & Smyth, G. K. edgeR: a Bioconductor package for differential expression analysis of digital gene expression data. *Bioinformatics* **26,** 139–140 (2010).
8. Kornienko, A. E. *et al.* Long non-coding RNAs display higher natural expression variation than protein-coding genes in healthy humans. *Genome biology* **17,** 14 (2016).
9. Tsoi, L. C. *et al.* Analysis of long non-coding RNAs highlights tissue-specific expression patterns and epigenetic profiles in normal and psoriatic skin. *Genome biology* **16,** 1 (2015).
10. McCarthy, D. J., Campbell, K. R., Lun, A. T. L. & Wills, Q. F. Scater: pre-processing, quality control, normalisation and visualisation of single-cell RNA-seq data in R. *Bioinformatics* **14 Jan.** doi:10.1093/bioinformatics/btw777. <http://dx.doi.org/10.1093/bioinformatics/btw777> (2017).
11. R Development Core Team. *R: A Language and Environment for Statistical Computing* ISBN 3-900051-07-0. R Foundation for Statistical Computing (Vienna, Austria, 2008). <http://www.R-project.org>.
12. Benidt, S. & Nettleton, D. SimSeq: a nonparametric approach to simulation of RNA-sequence datasets. *Bioinformatics* **31,** 2131–2140 (2015).
13. Strimmer, K. fdrtool: a versatile R package for estimating local and tail area-based false discovery rates. *Bioinformatics* **24,** 1461–1462 (2008).
14. Robinson, M. D. & Oshlack, A. A scaling normalization method for differential expression analysis of RNA-seq data. *Genome biology* **11,** 1 (2010).
