# Supplementary material for: Differential gene expression analysis tools exhibit substandard performance for long non-coding RNA-sequencing data
Source: Genome Biol. 2018 Jul 24;19:96. doi: 10.1186/s13059-018-1466-5 (PMC6058388; doi:10.1186/s13059-018-1466-5)
Supplement: Supplementary file 2 — Supplementary data. Detailed results of comparisons of normalization methods. (HTML 2067 kb) [file 13059_2018_1466_MOESM2_ESM.html]

Additional File 2: Comparing normalization methods


# Additional File 2: Comparing normalization methods

#### *Alemu Takele Assefa, Katrijn De Paepe, Celine Everaert, Pieter Mestdagh, Olivier Thas, Jo Vandesompele*

#### *February 26, 2018*

- 1 Normalization Methods
- 2 Comparison of normalization methods
  - 2.1 Preliminary processes
  - 2.2 Distribution of the relative-log-expression
  - 2.3 Distribution of coefficients of variations (CV)
  - 2.4 Effect of normalization methods on DGE analysis
- 3 Overall summary and conclusion
- 4 References

# 1 Normalization Methods

Normalization is an essential procedure in transcriptome data analysis. Its aim is to remove technical artifacts such as the differences in the number of reads per sample, biases introduced by library preparation protocols, sequencing platforms and nucleotide compositions (e.g. the GC-content) [1]. A number of normalization methods have been introduced that come with DGE analysis tools. Previous studies [1–4] have pointed out that the normalization procedure can affect the DE results. Therefore, comparing DE tools requires careful attention to the normalization methods. Hence, we first explored the performance of 5 normalization methods that are used in conjunction with the DE methods evaluated in our study. The normalization methods were compared using the metrics from Dillies et al. [1], such as their capability to reduce variability from technical sources, their capability of eliminating bias due to library size differences, and their effect on DGE analysis.

Five normalization methods are evaluated in this study: quantile normalization [5] implemented in limma (limmaQN), Trimmed Mean of M-values [6] implemented in edgeR, limma (limmaVoom and limmaVoom+QW), baySeq, and QuasiSeq, Medians of Ratios (MR) [7] implemented in DESeq, DESeq2, and limma (limmaVst), the goodness-of-fit statistics [8] approach in PoissonSeq, and the re-sampling technique [9] implemented in SAMSeq. To examine the effect of normalization on DGE analysis, we applied a moderated t-test [10] in combination to each of the five normalization methods. Afterwards, the extent of (dis)similarity of results is used as an indicator of normalization effect on DGE analysis.

**Quantile normalization (QN)**[5] makes the empirical distribution of gene expression levels from each sample identical. To do so, it forces each quantile to be the same across samples. Given a matrix of gene expression (genes are represented in rows and samples are in columns), QN involves three steps: order values in each sample, replace the values of each row with the average of that row, and finally re-order back to the original order.

**Trimmed Mean of M-values (TMM)**[6]: assumes that most genes are not DE. Considering one sample (typically the first) as a reference and others as test samples, it computes a TMM factor for each sample. For each test sample, TMM is computed as the weighted mean of log ratios between this test and the reference, after exclusion of the most expressed genes and the genes with the largest log ratios. Since most genes are assumed to be not DE, the TMM factor is expected be close to 1. Otherwise, its value provides an estimate of the correction factor that must be applied to the library sizes (and not the raw counts) in order to fulfill the assumption. In particular, the *calcNormFactors()* function in the edgeR package provides these scaling factors. To obtain normalized read counts, these normalization factors are re-scaled by the mean of the normalized library sizes. Normalized read counts are obtained by dividing raw read counts by these re-scaled normalization factors.

**DESeq**[7]: This normalization method is introduced with the DESeq package[7] (and hence we call it DESeq normalization). It also assumes that most genes are not DE. A DESeq scaling factor for a given sample is computed as the median of the ratio, for each gene, of its read count over its geometric mean across all samples. The underlying idea is that non-DE genes should have similar read counts across samples, leading to a ratio of 1. Assuming most genes are not DE, the median of this ratio for the lane provides an estimate of the correction factor that should be applied to all read counts of this sample to fulfill the assumption. The *estimateSizeFactors()* and *sizeFactors()* functions in the DESeq package provide the DESeq factors. To obtain the normalized counts, raw read counts are divided by the factor associated with their sequencing sample.

**PoissonSeq normalization**[8] estimates the sequencing depth for each sample based on counts from candidate genes. Genes with Poisson goodness-of-fit statistics within (\(\epsilon\), \(1-\epsilon\)) will be included to the candidate genes. \(\epsilon\) is 0.25 by default, such that 50% of genes are used to estimate the sequencing depth. The concept is similar to the total count normalization (TCN) except that calculation of the sequencing depth in TCN uses read-counts from all genes. PoissonSeq sequencing depth are obtained using the function *PS.Est.Depth()*, and subsequently normalized counts are obtained by scaling counts by the inverse of the estimated sequencing depth.

**SAMSeq normalization**[9] uses resampling strategy to alleviate the sequencing depth differences between samples. RNA fragment reads mapped to each feature are resampled under the assumption that read-counts follow Poisson distribution. Specifically, the so-called âPoisson re-samplingâ strategy resamples each sample to a sequencing depth that is the geometric mean of the sequencing depths for all experiments. To minimize its limitations for features with low counts, it repeats the resampling S times (S = 20 is recommended) and take the average.

# 2 Comparison of normalization methods

## 2.1 Preliminary processes

#### 2.1.0.1 Load required packages

```
library(edgeR)
library(DESeq2)
library(samr)
library(PoissonSeq)
library(preprocessCore)
library(ConsRank)
library(dplyr)
library(magrittr)
library(ggplot2)
require(gridExtra)
library(broman)
library(psych)
library(UpSetR) 
library(knitr)
library(kableExtra)
```

#### 2.1.0.2 Load working datasets

```
## [1] "CRC AZA data"
```

```
## [1] "... ... number of mRNA genes = 14658"
```

```
## [1] "... ... number of samples    = 6"
```

```
## [1] "NGP nutlin data"
```

```
## [1] "... ... number of mRNA genes   = 17489"
```

```
## [1] "... ... number of lncRNA genes = 8929"
```

```
## [1] "... ... number of samples      = 20"
```

```
## [1] "Hammer data"
```

```
## [1] "... ... number of mRAN genes = 15908"
```

```
## [1] "... ... number of samples    = 4"
```

```
## [1] "Bottomly data"
```

```
## [1] "... ... number of mRNA genes = 12784"
```

```
## [1] "... ... number of samples    = 21"
```

```
## [1] "GTEx data"
```

```
## [1] "... ... number of mRNA genes = 18636"
```

```
## [1] "... ... number of samples    = 40"
```

```
## [1] "Zhang data"
```

```
## [1] "... ... number of mRNA genes   = 19381"
```

```
## [1] "... ... number of lncRNA genes = 12509"
```

```
## [1] "... ... number of samples      = 40"
```

#### 2.1.0.3 Load functions created to facilitate comparison of normalization methods

```
source("functions for normalization comparison.R")
```

#### 2.1.0.4 Normalize datasets

```
  QN.counts.Zhang          <- Normalize(counts.Zhang, group.Zhang, norm.method="QN")
  TMM.counts.Zhang         <- Normalize(counts.Zhang, group.Zhang, norm.method="TMM")
  DESeq.counts.Zhang       <- Normalize(counts.Zhang, group.Zhang, norm.method="DESeq")
  PoissonSeq.counts.Zhang  <- Normalize(counts.Zhang, group.Zhang, norm.method="PoissonSeq")
  SAMSeq.counts.Zhang      <- Normalize(counts.Zhang, group.Zhang, norm.method="SAMSeq")
  
  QN.counts.NGP          <- Normalize(counts.NGP, group.NGP, norm.method="QN")
  TMM.counts.NGP         <- Normalize(counts.NGP, group.NGP, norm.method="TMM")
  DESeq.counts.NGP       <- Normalize(counts.NGP, group.NGP, norm.method="DESeq")
  PoissonSeq.counts.NGP  <- Normalize(counts.NGP, group.NGP, norm.method="PoissonSeq")
  SAMSeq.counts.NGP      <- Normalize(counts.NGP, group.NGP, norm.method="SAMSeq")
  
  QN.counts.CRC          <- Normalize(counts.CRC, group.CRC, norm.method="QN")
  TMM.counts.CRC         <- Normalize(counts.CRC, group.CRC, norm.method="TMM")
  DESeq.counts.CRC       <- Normalize(counts.CRC, group.CRC, norm.method="DESeq")
  PoissonSeq.counts.CRC  <- Normalize(counts.CRC, group.CRC, norm.method="PoissonSeq")
  SAMSeq.counts.CRC      <- Normalize(counts.CRC, group.CRC, norm.method="SAMSeq")
  
  QN.counts.Hammer          <- Normalize(counts.Hammer, group.Hammer, norm.method="QN")
  TMM.counts.Hammer         <- Normalize(counts.Hammer, group.Hammer, norm.method="TMM")
  DESeq.counts.Hammer       <- Normalize(counts.Hammer, group.Hammer, norm.method="DESeq")
  PoissonSeq.counts.Hammer  <- Normalize(counts.Hammer, group.Hammer, norm.method="PoissonSeq")
  SAMSeq.counts.Hammer      <- Normalize(counts.Hammer, group.Hammer, norm.method="SAMSeq")
  
  QN.counts.Bottomly          <- Normalize(counts.Bottomly, group.Bottomly, norm.method="QN")
  TMM.counts.Bottomly         <- Normalize(counts.Bottomly, group.Bottomly, norm.method="TMM")
  DESeq.counts.Bottomly       <- Normalize(counts.Bottomly, group.Bottomly, norm.method="DESeq")
  PoissonSeq.counts.Bottomly  <- Normalize(counts.Bottomly, group.Bottomly, norm.method="PoissonSeq")
  SAMSeq.counts.Bottomly      <- Normalize(counts.Bottomly, group.Bottomly, norm.method="SAMSeq")
  
  QN.counts.GTEx          <- Normalize(counts.GTEx, group.GTEx, norm.method="QN")
  TMM.counts.GTEx         <- Normalize(counts.GTEx, group.GTEx, norm.method="TMM")
  DESeq.counts.GTEx       <- Normalize(counts.GTEx, group.GTEx, norm.method="DESeq")
  PoissonSeq.counts.GTEx  <- Normalize(counts.GTEx, group.GTEx, norm.method="PoissonSeq")
  SAMSeq.counts.GTEx      <- Normalize(counts.GTEx, group.GTEx, norm.method="SAMSeq")
```

## 2.2 Distribution of the relative-log-expression

The relative-log-expression (RLE) plots [11] are used to show the distribution of relative expression in each sample before and after normalization (QN, DESeq, TMM, PoissonSeq, and SAMSeq) for the 6 datasets. Box plots of RLE for each sample indicate the presence of unwanted variation related to sample quality and/or library size differences. Ideally, the boxes show small spread around 0. A sample that has quality problems may result in a box that has greater spread or is not centered near 0 [11]. A constant 1 is added to each observation in order to avoid the problem of taking logarithmic of 0. The two colors represent the two conditions. For clarity, the title of each plot refers the type of normalization method applied.

#### 2.2.0.1 RLE plots of the CRC AZA data

Figure S1

#### 2.2.0.2 RLE plots of the Hammer data

Figure S2

#### 2.2.0.3 RLE plots of the Bottomly data

Figure S3

#### 2.2.0.4 RLE plots of the GTEx data

A random selection of 20 samples in each group is used  Figure S4

#### 2.2.0.5 RLE plots of the Zhang data

A random selection of 20 samples in each group is used  Figure S5

#### 2.2.0.6 RLE plots of the NGP nutlin data

Figure S6

## 2.3 Distribution of coefficients of variations (CV)

Violin plots are used to compare and demonstrate the distribution of empirically calculated gene-wise group-specific coefficients of variation (standard deviation/mean) before and after normalization for each dataset. The three black horizontal lines in each violin plot represent the third, second, and first quartiles of CV values respectively from upper to lower.

#### 2.3.0.1 Distribution of coefficients of variation from the CRC AZA data

Figure S7

#### 2.3.0.2 Distribution of coefficients of variation from the Hammer data

Figure S8

#### 2.3.0.3 Distribution of coefficients of variation from the Bottomly data

Figure S9

#### 2.3.0.4 Distribution of coefficients of variation from the GTEx data

A random selection of 20 samples in each group is used  Figure S10

#### 2.3.0.5 Distribution of coefficients of variation from the Zhang data

A random selection of 20 samples in each group is used  Figure S11

#### 2.3.0.6 Distribution of coefficients of variation from the NGP nutlin data

Figure S12

## 2.4 Effect of normalization methods on DGE analysis

To examine the effect of normalization methods on differential gene expression (DGE) analysis, we applied moderated t-test in combination to each of the five normalization methods. The moderated t-test was applied on a log2-transformed normalized counts with a constant 1 is added to avoid taking logarithm of 0. Genes are called significantly differentially expressed (SDE) at 5% nominal FDR. Afterwards, the extent of dissimilarity of results was used as an indicator of normalization effect on DGE analysis.

#### 2.4.0.1 The CRC AZA data

|  |  |  |  |  |  |  |  |  |  |  |  |  |  |  |  |  |  |  |  |  |  |  |  |  |  |  |  |  |  |  |  |  |  |  |  |  |  |  |  |  |  |  |  |  |  |  |  |  |  |  |  |  |  |  |  |  |  |  |  |  |  |  |  |  |  |  |  |  |  |  |  |  |  |  |  |  |  |  |  |  |  |  |  |  |  |  |  |  |  |  |  |  |  |  |  |  |  |  |  |
| --- | --- | --- | --- | --- | --- | --- | --- | --- | --- | --- | --- | --- | --- | --- | --- | --- | --- | --- | --- | --- | --- | --- | --- | --- | --- | --- | --- | --- | --- | --- | --- | --- | --- | --- | --- | --- | --- | --- | --- | --- | --- | --- | --- | --- | --- | --- | --- | --- | --- | --- | --- | --- | --- | --- | --- | --- | --- | --- | --- | --- | --- | --- | --- | --- | --- | --- | --- | --- | --- | --- | --- | --- | --- | --- | --- | --- | --- | --- | --- | --- | --- | --- | --- | --- | --- | --- | --- | --- | --- | --- | --- | --- | --- | --- | --- | --- | --- | --- | --- |
| Table 1A: Number of SDE genes shared by pairs of normalization methods  |  | Raw Count | QN | TMM | DESeq | PoissonSeq | SAMSeq | | --- | --- | --- | --- | --- | --- | --- | | Raw Count | 722 | 642 | 689 | 686 | 691 | 691 | | QN | 642 | 3813 | 3075 | 3052 | 2941 | 2941 | | TMM | 689 | 3075 | 3906 | 3793 | 3443 | 3426 | | DESeq | 686 | 3052 | 3793 | 3894 | 3504 | 3486 | | PoissonSeq | 691 | 2941 | 3443 | 3504 | 3803 | 3778 | | SAMSeq | 691 | 2941 | 3426 | 3486 | 3778 | 3783 | | Table 1B: Pairwise proportion of overlap between normalization methods.  |  | Raw Count | QN | TMM | DESeq | PoissonSeq | SAMSeq | | --- | --- | --- | --- | --- | --- | --- | | Raw Count | 1.000 | 0.165 | 0.175 | 0.175 | 0.180 | 0.181 | | QN | 0.165 | 1.000 | 0.662 | 0.656 | 0.629 | 0.632 | | TMM | 0.175 | 0.662 | 1.000 | 0.947 | 0.807 | 0.804 | | DESeq | 0.175 | 0.656 | 0.947 | 1.000 | 0.836 | 0.832 | | PoissonSeq | 0.180 | 0.629 | 0.807 | 0.836 | 1.000 | 0.992 | | SAMSeq | 0.181 | 0.632 | 0.804 | 0.832 | 0.992 | 1.000 | |

Figure S13: UpSet plot to visualize intersection size between the five normalization methods based on analysis of the CRC AZA data using moderated t test.

#### 2.4.0.2 The Hammer data

|  |  |  |  |  |  |  |  |  |  |  |  |  |  |  |  |  |  |  |  |  |  |  |  |  |  |  |  |  |  |  |  |  |  |  |  |  |  |  |  |  |  |  |  |  |  |  |  |  |  |  |  |  |  |  |  |  |  |  |  |  |  |  |  |  |  |  |  |  |  |  |  |  |  |  |  |  |  |  |  |  |  |  |  |  |  |  |  |  |  |  |  |  |  |  |  |  |  |  |  |
| --- | --- | --- | --- | --- | --- | --- | --- | --- | --- | --- | --- | --- | --- | --- | --- | --- | --- | --- | --- | --- | --- | --- | --- | --- | --- | --- | --- | --- | --- | --- | --- | --- | --- | --- | --- | --- | --- | --- | --- | --- | --- | --- | --- | --- | --- | --- | --- | --- | --- | --- | --- | --- | --- | --- | --- | --- | --- | --- | --- | --- | --- | --- | --- | --- | --- | --- | --- | --- | --- | --- | --- | --- | --- | --- | --- | --- | --- | --- | --- | --- | --- | --- | --- | --- | --- | --- | --- | --- | --- | --- | --- | --- | --- | --- | --- | --- | --- | --- | --- |
| Table 2A: Number of SDE genes shared by pairs of normalization methods  |  | Raw Count | QN | TMM | DESeq | PoissonSeq | SAMSeq | | --- | --- | --- | --- | --- | --- | --- | | Raw Count | 6069 | 2209 | 2262 | 2201 | 2259 | 2264 | | QN | 2209 | 3055 | 2755 | 2727 | 2767 | 2774 | | TMM | 2262 | 2755 | 3103 | 2938 | 3051 | 3056 | | DESeq | 2201 | 2727 | 2938 | 3075 | 2888 | 2895 | | PoissonSeq | 2259 | 2767 | 3051 | 2888 | 3094 | 3087 | | SAMSeq | 2264 | 2774 | 3056 | 2895 | 3087 | 3108 | | Table 2B: Pairwise proportion of overlap between normalization methods.  |  | Raw Count | QN | TMM | DESeq | PoissonSeq | SAMSeq | | --- | --- | --- | --- | --- | --- | --- | | Raw Count | 1.000 | 0.319 | 0.327 | 0.317 | 0.327 | 0.327 | | QN | 0.319 | 1.000 | 0.810 | 0.801 | 0.818 | 0.819 | | TMM | 0.327 | 0.810 | 1.000 | 0.907 | 0.970 | 0.969 | | DESeq | 0.317 | 0.801 | 0.907 | 1.000 | 0.880 | 0.880 | | PoissonSeq | 0.327 | 0.818 | 0.970 | 0.880 | 1.000 | 0.991 | | SAMSeq | 0.327 | 0.819 | 0.969 | 0.880 | 0.991 | 1.000 | |

Figure S14: UpSet plot to visualize intersection size between the five normalization methods based on analysis of the Hammer data using moderated t test.

#### 2.4.0.3 The Bottomly data

|  |  |  |  |  |  |  |  |  |  |  |  |  |  |  |  |  |  |  |  |  |  |  |  |  |  |  |  |  |  |  |  |  |  |  |  |  |  |  |  |  |  |  |  |  |  |  |  |  |  |  |  |  |  |  |  |  |  |  |  |  |  |  |  |  |  |  |  |  |  |  |  |  |  |  |  |  |  |  |  |  |  |  |  |  |  |  |  |  |  |  |  |  |  |  |  |  |  |  |  |
| --- | --- | --- | --- | --- | --- | --- | --- | --- | --- | --- | --- | --- | --- | --- | --- | --- | --- | --- | --- | --- | --- | --- | --- | --- | --- | --- | --- | --- | --- | --- | --- | --- | --- | --- | --- | --- | --- | --- | --- | --- | --- | --- | --- | --- | --- | --- | --- | --- | --- | --- | --- | --- | --- | --- | --- | --- | --- | --- | --- | --- | --- | --- | --- | --- | --- | --- | --- | --- | --- | --- | --- | --- | --- | --- | --- | --- | --- | --- | --- | --- | --- | --- | --- | --- | --- | --- | --- | --- | --- | --- | --- | --- | --- | --- | --- | --- | --- | --- | --- |
| Table 3A: Number of SDE genes shared by pairs of normalization methods  |  | Raw Count | QN | TMM | DESeq | PoissonSeq | SAMSeq | | --- | --- | --- | --- | --- | --- | --- | | Raw Count | 157 | 153 | 154 | 152 | 152 | 152 | | QN | 153 | 984 | 955 | 951 | 935 | 932 | | TMM | 154 | 955 | 999 | 966 | 947 | 944 | | DESeq | 152 | 951 | 966 | 995 | 963 | 959 | | PoissonSeq | 152 | 935 | 947 | 963 | 1014 | 1008 | | SAMSeq | 152 | 932 | 944 | 959 | 1008 | 1018 | | Table 3B: Pairwise proportion of overlap between normalization methods.  |  | Raw Count | QN | TMM | DESeq | PoissonSeq | SAMSeq | | --- | --- | --- | --- | --- | --- | --- | | Raw Count | 1.000 | 0.155 | 0.154 | 0.152 | 0.149 | 0.149 | | QN | 0.155 | 1.000 | 0.929 | 0.925 | 0.880 | 0.871 | | TMM | 0.154 | 0.929 | 1.000 | 0.940 | 0.888 | 0.880 | | DESeq | 0.152 | 0.925 | 0.940 | 1.000 | 0.921 | 0.910 | | PoissonSeq | 0.149 | 0.880 | 0.888 | 0.921 | 1.000 | 0.984 | | SAMSeq | 0.149 | 0.871 | 0.880 | 0.910 | 0.984 | 1.000 | |

Figure S15: UpSet plot to visualize intersection size between the five normalization methods based on analysis of the Bottomly data using moderated t test.

#### 2.4.0.4 The GTEx data

A random selection of 20 samples in each group is used.

|  |  |  |  |  |  |  |  |  |  |  |  |  |  |  |  |  |  |  |  |  |  |  |  |  |  |  |  |  |  |  |  |  |  |  |  |  |  |  |  |  |  |  |  |  |  |  |  |  |  |  |  |  |  |  |  |  |  |  |  |  |  |  |  |  |  |  |  |  |  |  |  |  |  |  |  |  |  |  |  |  |  |  |  |  |  |  |  |  |  |  |  |  |  |  |  |  |  |  |  |
| --- | --- | --- | --- | --- | --- | --- | --- | --- | --- | --- | --- | --- | --- | --- | --- | --- | --- | --- | --- | --- | --- | --- | --- | --- | --- | --- | --- | --- | --- | --- | --- | --- | --- | --- | --- | --- | --- | --- | --- | --- | --- | --- | --- | --- | --- | --- | --- | --- | --- | --- | --- | --- | --- | --- | --- | --- | --- | --- | --- | --- | --- | --- | --- | --- | --- | --- | --- | --- | --- | --- | --- | --- | --- | --- | --- | --- | --- | --- | --- | --- | --- | --- | --- | --- | --- | --- | --- | --- | --- | --- | --- | --- | --- | --- | --- | --- | --- | --- | --- |
| Table 4A: Number of SDE genes shared by pairs of normalization methods  |  | Raw Count | QN | TMM | DESeq | PoissonSeq | SAMSeq | | --- | --- | --- | --- | --- | --- | --- | | Raw Count | 464 | 442 | 447 | 448 | 446 | 446 | | QN | 442 | 2382 | 1982 | 2014 | 2017 | 2012 | | TMM | 447 | 1982 | 2317 | 2238 | 2295 | 2287 | | DESeq | 448 | 2014 | 2238 | 2325 | 2262 | 2255 | | PoissonSeq | 446 | 2017 | 2295 | 2262 | 2398 | 2380 | | SAMSeq | 446 | 2012 | 2287 | 2255 | 2380 | 2383 | | Table 4B: Pairwise proportion of overlap between normalization methods.  |  | Raw Count | QN | TMM | DESeq | PoissonSeq | SAMSeq | | --- | --- | --- | --- | --- | --- | --- | | Raw Count | 1.000 | 0.184 | 0.192 | 0.191 | 0.185 | 0.186 | | QN | 0.184 | 1.000 | 0.729 | 0.748 | 0.730 | 0.731 | | TMM | 0.192 | 0.729 | 1.000 | 0.931 | 0.948 | 0.948 | | DESeq | 0.191 | 0.748 | 0.931 | 1.000 | 0.919 | 0.919 | | PoissonSeq | 0.185 | 0.730 | 0.948 | 0.919 | 1.000 | 0.991 | | SAMSeq | 0.186 | 0.731 | 0.948 | 0.919 | 0.991 | 1.000 | |

Figure S16: UpSet plot to visualize intersection size between the five normalization methods based on analysis of the GTEx data using moderated t test.

#### 2.4.0.5 The Zhang data

A random selection of 20 samples in each group is used.

|  |  |  |  |  |  |  |  |  |  |  |  |  |  |  |  |  |  |  |  |  |  |  |  |  |  |  |  |  |  |  |  |  |  |  |  |  |  |  |  |  |  |  |  |  |  |  |  |  |  |  |  |  |  |  |  |  |  |  |  |  |  |  |  |  |  |  |  |  |  |  |  |  |  |  |  |  |  |  |  |  |  |  |  |  |  |  |  |  |  |  |  |  |  |  |  |  |  |  |  |
| --- | --- | --- | --- | --- | --- | --- | --- | --- | --- | --- | --- | --- | --- | --- | --- | --- | --- | --- | --- | --- | --- | --- | --- | --- | --- | --- | --- | --- | --- | --- | --- | --- | --- | --- | --- | --- | --- | --- | --- | --- | --- | --- | --- | --- | --- | --- | --- | --- | --- | --- | --- | --- | --- | --- | --- | --- | --- | --- | --- | --- | --- | --- | --- | --- | --- | --- | --- | --- | --- | --- | --- | --- | --- | --- | --- | --- | --- | --- | --- | --- | --- | --- | --- | --- | --- | --- | --- | --- | --- | --- | --- | --- | --- | --- | --- | --- | --- | --- | --- |
| Table 5A: Number of SDE genes shared by pairs of normalization methods  |  | Raw Count | QN | TMM | DESeq | PoissonSeq | SAMSeq | | --- | --- | --- | --- | --- | --- | --- | | Raw Count | 2168 | 1660 | 1537 | 1611 | 1744 | 1743 | | QN | 1660 | 2523 | 2035 | 2063 | 2228 | 2241 | | TMM | 1537 | 2035 | 2240 | 2162 | 2123 | 2132 | | DESeq | 1611 | 2063 | 2162 | 2256 | 2161 | 2165 | | PoissonSeq | 1744 | 2228 | 2123 | 2161 | 2482 | 2468 | | SAMSeq | 1743 | 2241 | 2132 | 2165 | 2468 | 2500 | | Table 5B: Pairwise proportion of overlap between normalization methods.  |  | Raw Count | QN | TMM | DESeq | PoissonSeq | SAMSeq | | --- | --- | --- | --- | --- | --- | --- | | Raw Count | 1.000 | 0.548 | 0.535 | 0.573 | 0.600 | 0.596 | | QN | 0.548 | 1.000 | 0.746 | 0.760 | 0.802 | 0.806 | | TMM | 0.535 | 0.746 | 1.000 | 0.926 | 0.817 | 0.817 | | DESeq | 0.573 | 0.760 | 0.926 | 1.000 | 0.839 | 0.836 | | PoissonSeq | 0.600 | 0.802 | 0.817 | 0.839 | 1.000 | 0.982 | | SAMSeq | 0.596 | 0.806 | 0.817 | 0.836 | 0.982 | 1.000 | |

Figure S17: UpSet plot to visualize intersection size between the five normalization methods based on analysis of the Zhang data using moderated t test.

#### 2.4.0.6 NGP nutlin data

|  |  |  |  |  |  |  |  |  |  |  |  |  |  |  |  |  |  |  |  |  |  |  |  |  |  |  |  |  |  |  |  |  |  |  |  |  |  |  |  |  |  |  |  |  |  |  |  |  |  |  |  |  |  |  |  |  |  |  |  |  |  |  |  |  |  |  |  |  |  |  |  |  |  |  |  |  |  |  |  |  |  |  |  |  |  |  |  |  |  |  |  |  |  |  |  |  |  |  |  |
| --- | --- | --- | --- | --- | --- | --- | --- | --- | --- | --- | --- | --- | --- | --- | --- | --- | --- | --- | --- | --- | --- | --- | --- | --- | --- | --- | --- | --- | --- | --- | --- | --- | --- | --- | --- | --- | --- | --- | --- | --- | --- | --- | --- | --- | --- | --- | --- | --- | --- | --- | --- | --- | --- | --- | --- | --- | --- | --- | --- | --- | --- | --- | --- | --- | --- | --- | --- | --- | --- | --- | --- | --- | --- | --- | --- | --- | --- | --- | --- | --- | --- | --- | --- | --- | --- | --- | --- | --- | --- | --- | --- | --- | --- | --- | --- | --- | --- | --- | --- |
| Table 6A: Number of SDE genes shared by pairs of normalization methods  |  | Raw Count | QN | TMM | DESeq | PoissonSeq | SAMSeq | | --- | --- | --- | --- | --- | --- | --- | | Raw Count | 14736 | 11029 | 14670 | 14685 | 14407 | 14405 | | QN | 11029 | 14139 | 11544 | 11531 | 11669 | 11673 | | TMM | 14670 | 11544 | 15680 | 15396 | 14852 | 14853 | | DESeq | 14685 | 11531 | 15396 | 15719 | 15175 | 15174 | | PoissonSeq | 14407 | 11669 | 14852 | 15175 | 16003 | 15945 | | SAMSeq | 14405 | 11673 | 14853 | 15174 | 15945 | 16008 | | Table 6B: Pairwise proportion of overlap between normalization methods.  |  | Raw Count | QN | TMM | DESeq | PoissonSeq | SAMSeq | | --- | --- | --- | --- | --- | --- | --- | | Raw Count | 1.000 | 0.618 | 0.932 | 0.931 | 0.882 | 0.882 | | QN | 0.618 | 1.000 | 0.632 | 0.629 | 0.632 | 0.632 | | TMM | 0.932 | 0.632 | 1.000 | 0.962 | 0.882 | 0.882 | | DESeq | 0.931 | 0.629 | 0.962 | 1.000 | 0.917 | 0.917 | | PoissonSeq | 0.882 | 0.632 | 0.882 | 0.917 | 1.000 | 0.992 | | SAMSeq | 0.882 | 0.632 | 0.882 | 0.917 | 0.992 | 1.000 | |

Figure S18: UpSet plot to visualize intersection size between the five normalization methods based on analysis of the NGP nutlin data using moderated t test.

# 3 Overall summary and conclusion

The large difference in the number of reads per sample (library size) is one main hamper for direct comparison of samples. This systematic technical variation can potentially confound the true biological differences. The primary objective of normalization is to alleviate such unwanted variation prior to the down-stream analysis. The distribution of normalized counts is one and the simplest way to demonstrate the existence of unwanted variation related the difference in library sizes. From results presented in [Section 2.2][Distribution of raw and normalized counts], box plots of log-transformed counts showed that for all six datasets, all normalization methods succeeded in aligning the sample specific distributions and hence no library size effects were noticeable after normalization.

Studies demonstrated that the gene wise coefficients of variation (CV) has two components, i.e. a technical and a biological component [7, 12]. Since normalization aims at removing sources of technical variations, the overall variability (as measured by CV) across samples is expected to be lower (only biological variation remains) for normalized counts compared to that of non-normalized counts. In order to ascertain this assumption, we compared the empirical distribution of condition-specific gene-wise CV (= standard deviation divided by arithmetic mean of counts) computed before and after normalization across the six datasets. This metric was significantly reduced for all datasets by all normalization procedures (see Section 2.3).

To examine the effect of normalization on DGE analysis, we applied a moderated t-test [10] in combination with each of the five normalization methods. Afterwards, the extent of (dis)similarity of results is used as an indicator of normalization effect on DGE analysis. Similarity between any two normalization methods is quantified using the number of SDE genes they share in common (at 5% FDR), as well as proportion of overlap. This result is visualized using UpSet diagram [13] (see Section 2.4). The overlap of DE genes with different normalization methods was generally high (see Section 2.4). Ignoring quantile normalization (QN), on average (across the 6 dataset) a minimum of 86% similarity was observed. QN-based DE analysis gives deviating results, particularly for designs with small numbers of replicates ($<$5); the average minimum proportion of similarity was 70.1%. In line with this result for QN, the UpSet plot shows that the DGE test with QN results in relatively higher number of SDE genes that are not shared with the other procedures. On the other hand, TMM showed a minimum of 90.3%, 80.7%, and 80.3% proportion of overlap with DESeq, PoissonSeq and SAMSeq normalizations, respectively. DESeq showed a minimum of 83.6% and 83.2% similarity with PoissonSeq and SAMSeq, respectively. In addition, PoissonSeq and SAMSeq showed the highest similarity, a minimum of 98.2% proportion of overlap. Overall, the results suggest that all normalization methods have very similar performance, except QN.

# 4 References


1. Dillies M-A, Rau A, Aubert J, Hennequet-Antier C, Jeanmougin M, Servant N, et al. A comprehensive evaluation of normalization methods for illumina high-throughput rna sequencing data analysis. Briefings in bioinformatics. 2013;14:671–83.

2. Zyprych-Walczak J, Szabelska A, Handschuh L, Górczak K, Klamecka K, Figlerowicz M, et al. The impact of normalization methods on rna-seq data analysis. BioMed research international. 2015;2015.

3. Bullard JH, Purdom E, Hansen KD, Dudoit S. Evaluation of statistical methods for normalization and differential expression in mRNA-seq experiments. BMC bioinformatics. 2010;11:94.

4. Rapaport F, Khanin R, Liang Y, Pirun M, Krek A, Zumbo P, et al. Comprehensive evaluation of differential gene expression analysis methods for rna-seq data. Genome biology. 2013;14:3158.

5. Bolstad BM, Irizarry RA, Åstrand M, Speed TP. A comparison of normalization methods for high density oligonucleotide array data based on variance and bias. Bioinformatics. 2003;19:185–93.

6. Robinson MD, Oshlack A. A scaling normalization method for differential expression analysis of rna-seq data. Genome biology. 2010;11:R25.

7. Anders S, Huber W. Differential expression analysis for sequence count data. Genome biology. 2010;11:R106.

8. Li J, Witten DM, Johnstone IM, Tibshirani R. Normalization, testing, and false discovery rate estimation for rna-sequencing data. Biostatistics. 2012;13:523–38.

9. Li J, Tibshirani R. Finding consistent patterns: A nonparametric approach for identifying differential expression in rna-seq data. Statistical methods in medical research. 2013;22:519–36.

10. Ritchie ME, Phipson B, Wu D, Hu Y, Law CW, Shi W, et al. Limma powers differential expression analyses for rna-sequencing and microarray studies. Nucleic acids research. 2015;43:e47–7.

11. Gentleman R, Carey V, Huber W, Irizarry R, Dudoit S. Bioinformatics and computational biology solutions using r and bioconductor. Springer Science & Business Media; 2006.

12. Robinson MD, Smyth GK. Small-sample estimation of negative binomial dispersion, with applications to sage data. Biostatistics. 2007;9:321–32.

13. Lex A, Gehlenborg N, Strobelt H, Vuillemot R, Pfister H. UpSet: Visualization of intersecting sets. IEEE transactions on visualization and computer graphics. 2014;20:1983–92.
