# Supplementary material for: Differential gene expression analysis tools exhibit substandard performance for long non-coding RNA-sequencing data
Source: Genome Biol. 2018 Jul 24;19:96. doi: 10.1186/s13059-018-1466-5 (PMC6058388; doi:10.1186/s13059-018-1466-5)
Supplement: Supplementary file 3 — Supplementary data. Detailed results of concordance analysis of DE tools. (HTML 15901 kb) [file 13059_2018_1466_MOESM3_ESM.html]

Additional File 3:Concordance analysis for individual dataset


# Additional File 3:Concordance analysis for individual dataset

#### *Alemu Takele Assefa, Katrijn De Paepe, Celine Everaert, Pieter Mestdagh, Olivier Thas, Jo Vandesompele*

#### *February 26, 2018*

- 1 Analysis of the CRC AZA data
  - 1.1 Number of SDE genes
  - 1.2 Proportion of overlap
  - 1.3 Gene ranking agreement
  - 1.4 Similarity of log-fold-change estimates
  - 1.5 Distribution of raw p-values
- 2 Analysis of the Bottomly data
  - 2.1 Number of SDE genes
  - 2.2 Proportion of overlap
  - 2.3 Gene ranking agreement
  - 2.4 Similarity of log-fold-change estimates
  - 2.5 Distribution of raw p-values
- 3 Analysis of the Hammer data
  - 3.1 Number of SDE genes
  - 3.2 Proportion of overlap
  - 3.3 Gene ranking agreement
  - 3.4 Similarity of log-fold-change estimates
  - 3.5 Distribution of raw p-values
- 4 Analysis of the GTEx data
  - 4.1 Number of SDE genes
  - 4.2 Proportion of overlap
  - 4.3 Gene ranking agreement
  - 4.4 Similarity of log-fold-change estimates
  - 4.5 Distribution of raw p-values
- 5 Analysis of the Zhang data
  - 5.1 Number of SDE genes
  - 5.2 Proportion of overlap
  - 5.3 Gene ranking agreement
  - 5.4 Similarity of log-fold-change estimates
  - 5.5 Distribution of raw p-values
- 6 Analysis of the NGP nutlin data
  - 6.1 Number of SDE genes
  - 6.2 Proportion of overlap
  - 6.3 Gene ranking agreement
  - 6.4 Similarity of log-fold-change estimates
  - 6.5 Distribution of raw p-values

---

This supplementary file contains the results of the DE tools concordance analysis for 6 individual datasets. The file is organized into 6 sections (for each dataset). As described in the main report, the concordance analysis focuses on the number of significantly differentially expressed (SDE) genes (at 5% FDR), similarity in terms of the set of SDE genes (overlap), gene ranking agreement (using \(\pi\)-score), and similarity of the log-fold-change (LFC) estimates. In addition to these concordance metrics, the distribution of raw p-values is visualized for DE tools that return raw p-values. At the end of the file, tables of p-values and LFC estimates for genes expressed only in one group of the Zhang data are included.

# 1 Analysis of the CRC AZA data

## 1.1 Number of SDE genes

Figure S1: **Concordance analysis of 25 DE pipelines based on the CRC AZA data**. (A) The number of SDE genes identified at 5% FDR. The colors represent the quartiles (4 equal groups, each with 25% weight) of the mean of normalized counts. (B) Proportion of SDE genes in each quartile group.

## 1.2 Proportion of overlap

Figure S2: **Concordance analysis of 25 DE pipelines based on the CRC AZA data**. Proportion of overlap (similarity) between pairs of DE pipelines in terms of the set of SDE genes at 5% FDR (left panel). Average proportion of overlap of across DE pipelines (right panel).

## 1.3 Gene ranking agreement

Figure S3: **Concordance analysis of 25 DE pipelines based on the CRC AZA data**. Spearman’s rank correlation coefficient between gene ranking (rank is based on \(\pi\)-score) of pairs of DE pipelines (left panel). Average rank correlation across DE pipelines (right panel).

## 1.4 Similarity of log-fold-change estimates

Figure S4: **Concordance analysis of 25 DE pipelines based on the CRC AZA data**. Pearson’s correlation coefficient between the log-fold-change estimates of pairs of DE pipelines (left panel). Average correlation across DE pipelines (right panel).

## 1.5 Distribution of raw p-values

Figure S5: **Concordance analysis of 25 DE pipelines based on the CRC AZA data**. Distribution of the raw p-values from selected DE tools.

# 2 Analysis of the Bottomly data

## 2.1 Number of SDE genes

Figure S1: **Concordance analysis of 25 DE pipelines based on the Bottomly data**. (A) The number of SDE genes identified at 5% FDR. The colors represent the quartiles (4 equal groups, each with 25% weight) of the mean of normalized counts. (B) Proportion of SDE genes in each quartile group.

## 2.2 Proportion of overlap

Figure S2: **Concordance analysis of 25 DE pipelines based on the Bottomly data**. Proportion of overlap (similarity) between pairs of DE pipelines in terms of the set of SDE genes at 5% FDR (left panel). Average proportion of overlap of across DE pipelines (right panel).

## 2.3 Gene ranking agreement

Figure S3: **Concordance analysis of 25 DE pipelines based on the Bottomly data**. Spearman’s rank correlation coefficient between gene ranking (rank is based on \(\pi\)-score) of pairs of DE pipelines (left panel). Average rank correlation across DE pipelines (right panel).

## 2.4 Similarity of log-fold-change estimates

Figure S4: **Concordance analysis of 25 DE pipelines based on the Bottomly data**. Pearson’s correlation coefficient between the log-fold-change estimates of pairs of DE pipelines (left panel). Average correlation across DE pipelines (right panel).

## 2.5 Distribution of raw p-values

Figure S5: **Concordance analysis of 25 DE pipelines based on the Bottomly data**. Distribution of the raw p-values from selected DE tools.

# 3 Analysis of the Hammer data

## 3.1 Number of SDE genes

Figure S1: **Concordance analysis of 25 DE pipelines based on the Hammer data**. (A) The number of SDE genes identified at 5% FDR. The colors represent the quartiles (4 equal groups, each with 25% weight) of the mean of normalized counts. (B) Proportion of SDE genes in each quartile group.

## 3.2 Proportion of overlap

Figure S2: **Concordance analysis of 25 DE pipelines based on the Hammer data**. Proportion of overlap (similarity) between pairs of DE pipelines in terms of the set of SDE genes at 5% FDR (left panel). Average proportion of overlap of across DE pipelines (right panel).

## 3.3 Gene ranking agreement

Figure S3: **Concordance analysis of 25 DE pipelines based on the Hammer data**. Spearman’s rank correlation coefficient between gene ranking (rank is based on \(\pi\)-score) of pairs of DE pipelines (left panel). Average rank correlation across DE pipelines (right panel).

## 3.4 Similarity of log-fold-change estimates

Figure S4: **Concordance analysis of 25 DE pipelines based on the Hammer data**. Pearson’s correlation coefficient between the log-fold-change estimates of pairs of DE pipelines (left panel). Average correlation across DE pipelines (right panel).

## 3.5 Distribution of raw p-values

Figure S5: **Concordance analysis of 25 DE pipelines based on the Hammer data**. Distribution of the raw p-values from selected DE tools.

# 4 Analysis of the GTEx data

## 4.1 Number of SDE genes

Figure S1: **Concordance analysis of 25 DE pipelines based on the GTEx data**. (A) The number of SDE genes identified at 5% FDR. The colors represent the quartiles (4 equal groups, each with 25% weight) of the mean of normalized counts. (B) Proportion of SDE genes in each quartile group.

## 4.2 Proportion of overlap

Figure S2: **Concordance analysis of 25 DE pipelines based on the GTEx data**. Proportion of overlap (similarity) between pairs of DE pipelines in terms of the set of SDE genes at 5% FDR (left panel). Average proportion of overlap of across DE pipelines (right panel).

## 4.3 Gene ranking agreement

Figure S3: **Concordance analysis of 25 DE pipelines based on the GTEx data**. Spearman’s rank correlation coefficient between gene ranking (rank is based on \(\pi\)-score) of pairs of DE pipelines (left panel). Average rank correlation across DE pipelines (right panel).

## 4.4 Similarity of log-fold-change estimates

Figure S4: **Concordance analysis of 25 DE pipelines based on the GTEx data**. Pearson’s correlation coefficient between the log-fold-change estimates of pairs of DE pipelines (left panel). Average correlation across DE pipelines (right panel).

## 4.5 Distribution of raw p-values

Figure S5: **Concordance analysis of 25 DE pipelines based on the GTEx data**. Distribution of the raw p-values from selected DE tools.

# 5 Analysis of the Zhang data

## 5.1 Number of SDE genes

Figure S1: **Concordance analysis of 25 DE pipelines based on the Zhang data**. (A) The number of SDE genes identified at 5% FDR. The colors represent the two gene biotypes (mRNA and lncRNA) of the mean of normalized counts. (B) Proportion of SDE genes in each biotype.

## 5.2 Proportion of overlap

Figure S2: **Concordance analysis of 25 DE pipelines based on the Zhang data**. Average proportion of overlap (similarity) of each DE pipeline with other pipelines. The extent of similarity for mRNA and lncRNA is presented separately.

## 5.3 Gene ranking agreement

Figure S3: **Concordance analysis of 25 DE pipelines based on the Zhang data**. Average Spearman’s rank correlation coefficient for each DE pipeline with other pipelines (rank is based on \(\pi\)-score). The extent of ranking agreement for mRNA and lncRNA is presented separately.

## 5.4 Similarity of log-fold-change estimates

Figure S4: **Concordance analysis of 25 DE pipelines based on the Zhang data**. The average Pearson’s correlation coefficient between the log-fold-change estimates of each DE pipelines with others. The degree of similarity for mRNA and lncRNA is presented separately.

## 5.5 Distribution of raw p-values

Figure S5: **Concordance analysis of 25 DE pipelines based on the Zhang data**. Distribution of the raw p-values from selected DE tools for mRNA and lncRNA genes.

# 6 Analysis of the NGP nutlin data

## 6.1 Number of SDE genes

Figure S1: **Concordance analysis of 25 DE pipelines based on the NGP nutlin data**. (A) The number of SDE genes identified at 5% FDR. The colors represent the two gene biotypes (mRNA and lncRNA) of the mean of normalized counts. (B) Proportion of SDE genes in each biotype.

## 6.2 Proportion of overlap

Figure S2: **Concordance analysis of 25 DE pipelines based on the NGP nutlin data**. Average proportion of overlap (similarity) of each DE pipeline with other pipelines. The extent of similarity for mRNA and lncRNA is presented separately.

## 6.3 Gene ranking agreement

Figure S3: **Concordance analysis of 25 DE pipelines based on the NGP nutlin data**. Average Spearman’s rank correlation coefficient for each DE pipeline with other pipelines (rank is based on \(\pi\)-score). The extent of ranking agreement for mRNA and lncRNA is presented separately.

## 6.4 Similarity of log-fold-change estimates

Figure S4: **Concordance analysis of 25 DE pipelines based on the NGP nutlin data**. The average Pearson’s correlation coefficient between the log-fold-change estimates of each DE pipelines with others. The degree of similarity for mRNA and lncRNA is presented separately.

## 6.5 Distribution of raw p-values

Figure S5: **Concordance analysis of 25 DE pipelines based on the NGP nutlin data**. Distribution of the raw p-values from selected DE tools for mRNA and lncRNA genes.
