# Supplementary material for: Differential gene expression analysis tools exhibit substandard performance for long non-coding RNA-sequencing data
Source: Genome Biol. 2018 Jul 24;19:96. doi: 10.1186/s13059-018-1466-5 (PMC6058388; doi:10.1186/s13059-018-1466-5)
Supplement: Supplementary file 4 — Supplementary data. Detailed results from the study of DE tools’ recovering ability of biological truth. (HTML 2102 kb) [file 13059_2018_1466_MOESM4_ESM.html]

Additional File 4: Performance of DE tools to recover biologiacl facts


# Additional File 4: Performance of DE tools to recover biologiacl facts

#### *Alemu Takele Assefa, Katrijn De Paepe, Celine Everaert, Pieter Mestdagh, Olivier Thas, Jo Vandesompele*

#### *February 26, 2018*

- 1 Recovering biological truth
  - 1.1 Recovering genes with evidence of gender-biased expression
  - 1.2 Recovering MYCN regulated genes
  - 1.3 Recovering TP53 pathway genes
- 2 Conclusions
- 3 References

# 1 Recovering biological truth

In addition to the concordance analysis, we also assessed the capability of the DE tools to recover genes with known evidence of differential expression in the benchmark datasets. To this purpose, results from three published studies were used to define the truth: genes with gender-biased expression, MYCN regulated genes, and TP53 pathway genes. Recovering ability is evaluated using four different metrics: number of recovered genes, similarity among DE pipelines in terms of the set of recovered genes, gene classification agreement with the truth, and gene set enrichment analysis (GSEA).

The number of recovered genes is the number of genes among the reference set that are identified as DE at a particular level of nominal FDR. The nominal FDR is harmonized with the level used in the truth study. On the other hand, similarity (overlap) of the set of recovered genes among DE pipelines is examined. Specifically, overlap proportion (\(p\_{ij}\)) between two DE tools \(i\) and \(j\), is calculated as

\[p\_{ij} = \frac{c\_{ij}}{a\_i+b\_j+c\_{ij}}\] where \(c\_{ij}\) is the number of commonly recovered genes (interaction) by tool \(i\) and \(j\), \(a\_i\) and \(b\_j\) are the number of genes recovered by only tool i or only tool j, respectively. If \(a\_i+b\_j+c\_{ij} = 0\), then \(p\_{ij}\) is set to 0. For each tool, the average proportion of overlap across all DE tools (except itself) is presented in the subsequent sections.

Classification agreement between each DE pipeline and the truth studies is examined using Fisher’s exact test. The objective is to test the chance of observing enrichment as extreme as the observed one due to chance. The test simultaneously assesses the true positive and true negative agreements. In particular, the truth is used as a control variable and DE classification (at a synchronized nominal FDR) by a particular pipeline is considered as a case variable. The test is done for each pipeline, and the \(-\log\_{10}\)(p-value) of the test (a one-sided test, “greater”) is used to rank DE pipelines.

Finally, GSEA is a powerful post-hoc analysis to validate results from DE analysis under the form of a ranked list of genes according to a particular suitable ranking variable, such as p-value and/or log-fold-change. Given a priori list of genes (truth reference set, e.g. TP53 pathway genes), the goal of GSEA is to determine whether members of the reference set tend to occur towards one of the extremes (top or bottom) of the list of ranked genes [1]. In this study, the \(\pi-\)score (a combination of p-value and log-fold-change) was used to rank genes in a benchmark data set. The analysis was done using GSEA software (version 3.0) [1]. Afterwards, DE pipelines are ranked using the normalized enrichment score (NES). A lower NES indicates a more random distribution of the reference genes through the list of ranked genes.

## 1.1 Recovering genes with evidence of gender-biased expression

In this section, we present the capability of test DE pipelines in recovering genes with evidence of gender-biased expression. In particular, a list of 54 genes with sex-biased expression evidence in at least one central nervous system (CNS) region (passing FDR 0.01) are obtained from [2] study. Among these 54 genes, 48 of them are available in the Zhang benchmark data (see Figure S2). DE tools’ recovering ability is then examined from comparing two groups of neuroblastoma samples in the Zhang data: girls (\(n\_1\)=44) versus boys (\(n\_2\)=48).

At 1% FDR, most of the pipelines recovered approximately 50% of the gender-biased genes, whereas SAMSeq and PoissonSeq detected none (these two also result in insignificant Fisher’s exact test (Figure S1)). However, ignoring SAMSeq, PoissonSeq, NOISeq, and DESeq, all pipelines showed more than 80% of similarity in terms of the set of recovered gender-biased genes. The Fisher’s exact test also reveals that the set of DE genes predicted by most DE pipelines is significantly associated with the reference set (exception for SAMSeq and PoissonSeq). Furthermore, the NES from GSEA indicates that apart from NOISeq, all DE pipelines ranked the reference genes at the top of all genes from comparing boys’ and girls’ neuroblastoma samples. In this regard, DESeq2 (all settings) and edgeR robust ranked at the top with slightly better performnace.

Figure S1: DE tools’ performance in recovering genes with evidence of gender-biased expression. The plot summarizes the fraction of recovered gender-biased genes (at 1% FDR, as used in [2]), the average proportion of overlap among DE pipelines, the odds ratio (log scale) of Fisher’s exact test (the error bars indicate the lower limit of a one sided 99% confidence interval), and normalized enrichment scores from GSEA (all reported NES have FDR \(<\) 0.0001).

Figure S2: Panels A and B show the coefficients of variation (CV) and log-fold-change estimates of the 48 gender-biased genes over their mean normalized count (based on their observed read counts from the Zhang data). The genes are numbered from 1 to 48. The name of the genes can be seen on the Y-axis of panel C, with gene numbers in square brackets and their chromosome number in parenthesis. Panel C shows the normalized raw counts of the 48 gender-biased genes for boys and girls with neuroblastoma (represented by colored bars) along with their average expression (represented by colored circles). On the right side of panel C, the number of DE pipelines (from a total of 25) that detected the corresponding gene (at 1% FDR) is indicated.

## 1.2 Recovering MYCN regulated genes

MYCN is an oncogenic transcription factor, activated in high-risk neuroblastoma through high-level gene amplification. A set of 157 MYCN pathway genes are obtained from a microarray study on primary tumors and shRNA model systems [3]. Among the 157 MYCN regulated genes, 141 of them are available in the Zhang data (Figure S4 shows the lower and upper 20% expressed MYCN regulated genes). By comparing MYCN amplified (\(n\_1\)=20) and MYCN non-amplified neuroblastoma samples (\(n\_2\)=20), DE pipelines’ recovering ability is further examined.

All pipelines detected less than 50% of MYCN regulated genes as DE, at 1% FDR (Figure S3). For NOISeq, DESeq and PoissonSeq this fraction drops below 20%. Except for NOISeq, DESeq, PoissonSeq, SAMSeq and baySeq, the average proportion of overlap is about 80%, indicating substantial similarity among pipelines in terms of the set of recovered genes. Similarly, the Fisher’s exact test indicates that DE classification by all DE pipelines is significantly associated (at 1% type I error rate) with the MYCN pathway classification (MYCN regulated vs. not regulated). However, the strength of the association was lower for PoissonSeq and SAMSeq. Additionally, the NES for SAMSeq is the lowest, implying that many of MYCN regulated genes are not on the top of all ranked genes by SAMSeq. This can be due to using the inverse of the Wilcoxon statistic instead of p-value when calculating \(\pi\)-score because SAMSeq does not return raw p-values.

Figure S3: DE tools’ performance in recovering MYCN pathway genes. The plot summarizes the fraction of recovered MYCN regulated genes (at 1% FDR), the average proportion of overlap among pipelines (similarity of the set of recovered genes), the odds ratio (log scale) of Fisher’s exact test (the error bars indicate the lower limit of a one sided 99% confidence interval), and normalized enrichment scores from GSEA (all reported NES have FDR \(<\) 0.0001).

Figure S4: Panels A and B show the coefficients of variation (CV) and log-fold-change estimates of the 141 MYCN regulated genes over their mean normalized count (based on their observed read counts from the Zhang data). The genes are numbered from 1 to 141. The name of the genes (the top and lower 20% expressed genes) can be seen on the Y-axis of panel C, with gene numbers between square brackets. Panel C shows the normalized raw counts of the the top and lower 20% expressed MYCN regulated genes for MYCN-not-amplified and MYCN-amplified neuroblastoma samples (represented by colored bars) along with their average expression (represented by colored circles). On the right side of panel C, the number of DE pipelines that detected the corresponding gene is indicated (from a total of 25).

## 1.3 Recovering TP53 pathway genes

TP53 is the most frequently mutated and best studied gene in cancer. The protein itself is a transcription factor regulating a set of genes involved in cell-cycle control and programmed cell death. The consensus between detected DE genes by the 25 DE pipelines and known TP53 pathway genes is examined using a set of 116 TP53 pathway genes obtained from [4]. In particular, we used DE results from the NGP nutlin data, in which 114 of the TP53 pathway genes are available. Nutlin-3a liberates TP53 from MDM2 mediated inhibition and hence activates the TP53 transcriptional response pathway. Similar to the previous results (the gender-biased expression and MYCN regulated genes), we examined fractions of recovered TP53 pathway genes, similarity among pipelines in terms of the set of recovered genes, classification agreement, and GSEA. Results can be seen in Figure S5.

Unlike the rate at which gender-biased and MYCN pathway genes are recovered, more than 95% of the TP53 pathway genes are identified as DE (at 1% FDR) by many of the pipelines. SAMSeq and PoissonSeq identified nearly 70% and 60% of them, respectively. This can be due to the higher average expression difference (up regulated) between the control and nutlin-3 samples that is observed in many of the TP53 regulated genes relative to the within variability in each group (Figure S6 Panel B and C). The overlap proportion is also strong among tools. However, DE classification by SAMSeq and PoissonSeq showed weak association with TP53 pathway that could be due to the higher false positives compared to true positives. For PoissonSeq, the association was not significant at 1% type-I error rate (as the lower limit of the one-sided 99% confidence interval crosses the null value, which is 0 = \(\log 1\)). From GSEA, all DE pipelines but SAMSeq had NES higher than 9 with minor differences between them.

Figure S5: DE tools’ performance in recovering TP53 pathway genes. The plot summarizes the proportion of recovered TP53 pathway genes (at 1% FDR), the average proportion of, the odds ratio (log scale) of Fisher’s exact test (the error bars indicate the lower limit of a one sided 99% confidence interval), and normalized enrichment scores from GSEA (all reported NES have FDR \(<\) 0.0001)

Figure S6: Panels A and B show the coefficients of variation (CV) and log-fold-change estimates of the 114 MYCN regulated genes over their mean normalized count (based on their observed read counts from the NGP nutlin data). The genes are numbered from 1 to 114. The name of the genes (the top and lower 20% expressed genes) can be seen on the Y-axis of panel C, with gene numbers are in a square brackets. Panel C shows the normalized raw counts of the the top and lower 20% expressed TP53 pathway genes forcontrol and nutlin-3 treated samples (represented by colored bars) along with their average expression (represented by colored circles). On the right side of panel C, the number of DE pipelines that detected the corresponding gene is indicated.

Furthermore, we explore the number of recovered TP53 pathway genes by expression magnitude (within the lower and upper 25% mean normalized count) and within genes with absolute value of the log2-fold-change \(<1\) and \(\ge 1\) (see Figure S7). For all DE tools, the proportion of detected genes (1% FDR) in the lower 25% expressed TP53 pathway genes is smaller than genes in the top 25%. Additionally, unlike the other tools, SAMSeq and PoissonSeq detected higher fraction of TP53 pathway genes with absolute LFC less than 1 than the opposite. This result for SAMSeq is in line with the result of another comparative study [5] indicating that rank based tools, such as SAMSeq, detect genes with biologically irrelevant signals. The possible explanation for this result is that SAMSeq uses rank scores instead of the actual observed counts that makes is robust from outliers but at the same time detects biologically negligible differences (among counts) as long as the order is maintained.

Figure S7: Fraction of DE TP53 pathway genes (at 5% FDR) in the lower/upper 25% expressed TP53 pathway genes and with less or more than 1 log2-fold-change.

# 2 Conclusions

To come to an overall conclusion across the three truth studies, consensus ranking of the 25 DE pipelines (Figure S8) is established using ConsRank (version 2.0.1) R software package [6]. The consensus rank is obtained for each of the four metrics across the three studies. Low ranks are given to tools with the worst performance of recovering the biological truth.

Despite the challenge of defining biological truth, several pipelines show relatively good performance in recovering the known truth, definitely considering that the experimental conditions are not identical. However, the performance variation among DE tools is not uniform across studies. In terms of the number of recovered genes and the degree of similarity to each other, the pipelines show substantial variation. In agreement with the concordance analysis, conservative tools (DESeq, baySeq, and NOISeq) recovered relatively few truly differentially expressed genes and showed lower similarity to other tools (in terms of the set of recovered genes). In contrast to the concordance analysis where SAMSeq and PoissonSeq were categorized with tools that detect the highest number of SDE genes, these tools recovered the least number of genes with known biological truth across the three studies and exhibited the lowest agreement with other tools. On the other hand, edgeR robust and DESeq2 pipelines ranked top, recovering the highest number of genes and showing strong consensus with other tools in terms of the set of recovered genes. In terms of the significance from Fisher’s exact, DESeq2 and limma tools are top ranked indicating that these tools show the highest agreement with the reference truth set of genes. In contrast, edgeR tools tend to occupy the lower rank. This shift relies on the number of false positives. Specifically, edgeR tools (also QuasiSeq tools) are relatively liberal and consequently result in high true positive and false positive rates. Similarly, baySeq showed a major shift towards the middle rank (it appears at low ranks in terms of other metrics) because of its higher true negative rate.

Rank of DE pipelines based on GSEA (specifically using NES) is more or less similar to the rank based on the first three metrics. The competition to occupy the top ranks is again between DESeq2, edgeR, and limma tools. However, in terms of NES, NOISeq also appears in the top rank, indicating that it distinguishes the reference set of genes among all ranked genes.

Generally, with respect to recovering genes with known biological truth, DESeq2 (both settings), edgeR (robust), and limma (voom+QW, voom, and Trended) outperformed all other tools, whereas PoissonSeq, SAMSeq, NOISeq, DESeq and QuasiSeq (both settings) showed inferior capability. However, for SAMSeq, baySeq, and NOISeq, the GSEA results should not be over interpreted because genes are ranked using \(\pi\)-score that use different statistic instead of p-values for these tools (the inverse of the Wilcoxon statistic for SAMSeq, the Bayesian false discovery rate for baySeq, and one minus the probability of differential expression for NOISeq).

Figure S8: The consensus rank of DE pipelines’ recovering ability across the three set of reference genes; genes with gender-biased expression, MYCN pathway genes, and TP53 pathway genes. A-consensus rank of DE tools based on proportion of recovered genes at 1% FDR, B-consensus rank of DE tools based on the average proportion of similarity in terms of the set of recovered genes, C-consensus rank of DE tools based on the \(-\log(p-values)\) of Fisher’s exact test, and D-consensus rank of DE tools based on normalized enrichment score from genes set enrichment analysis.

# 3 References


1. Subramanian A, Tamayo P, Mootha VK, Mukherjee S, Ebert BL, Gillette MA, et al. Gene set enrichment analysis: A knowledge-based approach for interpreting genome-wide expression profiles. Proceedings of the National Academy of Sciences. 2005;102:15545–50.

2. Trabzuni D, Ramasamy A, Imran S, Walker R, Smith C, Weale ME, et al. Widespread sex differences in gene expression and splicing in the adult human brain. Nature communications. 2013;4:2771.

3. Valentijn LJ, Koster J, Haneveld F, Aissa RA, Sluis P van, Broekmans ME, et al. Functional MYCN signature predicts outcome of neuroblastoma irrespective of MYCN amplification. Proceedings of the National Academy of Sciences. 2012;109:19190–5.

4. Fischer M. Census and evaluation of p53 target genes. Oncogene. 2017;36:3943.

5. Soneson C, Delorenzi M. A comparison of methods for differential expression analysis of rna-seq data. BMC bioinformatics. 2013;14:91.

6. Chermak E, Petta A, Serra L, Vangone A, Scarano V, Cavallo L, et al. CONSRANK: A server for the analysis, comparison and ranking of docking models based on inter-residue contacts. Bioinformatics. 2014;31:1481–3.
