# Supplementary material for: Differential gene expression analysis tools exhibit substandard performance for long non-coding RNA-sequencing data
Source: Genome Biol. 2018 Jul 24;19:96. doi: 10.1186/s13059-018-1466-5 (PMC6058388; doi:10.1186/s13059-018-1466-5)
Supplement: Supplementary file 5 — Supplementary data (A and B). Results of simulation quality assessment. (ZIP 8405 kb) [file 13059_2018_1466_MOESM5_ESM.zip › Additional file 5/Additional File 5B.html]

Additional File 5B: Additional Simulation Quality Assessments


# Additional File 5B: Additional Simulation Quality Assessments

#### *Alemu Takele Assefa, Katrijn De Paepe, Celine Everaert, Pieter Mestdagh, Olivier Thas, Jo Vandesompele*

#### *February 26, 2018*

- 1 Introduction
- 2 Quality assessment for Zhang simulation
- 3 Quality assessment for NGP nutlin simulation
- 4 Conclusion
- 5 References

# 1 Introduction

The simulated RNA-seq datasets were assessed by various quality metrics proposed by Soneson and Robinson [1] and implemented by their countsimQC R package (version 0.5.2). The metrics evaluate the average expressions of genes, variability, mean-variance relationship, correlations among replicates, correlations among genes, and fractions of zero counts. The quality assessments were positive in all aspects, and the reports generated by the countsimQC R package can be found in Additional File 5A. The assessments in the Additional File 5A were done jointly for mRNA and lncRNA. In this report, we demonstrate how the simulated mRNA and lncRNA expressions mimic features of their respective expressions from the source RNA-seq data. We particularly focus on the following characteristics

- distribution of gene-wise average expressions
- gene-wise total coefficients of variation (technical + biological)
- sample-to-sample correlation
- gene-wise fraction of zero expression
- fraction of outliers

Outlier counts were defined in terms of the median absolute deviation (MAD) as implemented in the scatter [2] package (*isOutlier()* function). Expressions are flagged as outliers if their MAD value is larger than 3 (the default is 5).

The assessment is applied for the two simulations (the Zhang and NGP nutlin), as these are the only simulations that comprise both mRNA and lncRNA.

# 2 Quality assessment for Zhang simulation

To avoid extended report, we show the quality assessments for one of the simulated data with 20 samples per group, 10,000 genes (constituting of 67% mRNA and 33% lncRNA) and 5% DE genes. This simulated data is compared with the source data that contains 10,000 genes with 70% mRNA and 30% lncRNA. The characteristics are demonstrated separately for lncRNA and mRNA expressions.

# 3 Quality assessment for NGP nutlin simulation

To avoid extended report, we show the quality assessments for one of the simulated data with 5 samples per group, 10,000 genes (constituting of 67% mRNA and 33% lncRNA), and 5% DE genes. This simulated data is compared with the source data (NGP nutlin) that contains 10,000 genes with 66% mRNA and 34% lncRNA. The characteristics are demonstrated separately for lncRNA and mRNA expressions.

```
##   mRNA lncRNA 
## 0.6563 0.3437
```

# 4 Conclusion

Generally, the low abundance and high variability of lncRNA data observed in the real RNA-seq datasets were also observed in the simulated datasets. The results demonstrate that the simulated lncRNAs expression retain their characteristics as in the source data. In conclusion, we believe that we have demonstrated that the simulated data sufficiently mimics real data so that our conclusions are relevant for the several types of source data that we included in our study.

# 5 References


1. Soneson C, Robinson MD. Towards unified quality verification of synthetic count data with countsimQC. Bioinformatics. 2017.

2. McCarthy DJ, Campbell KR, Lun ATL, Wills QF. Scater: Pre-processing, quality control, normalisation and visualisation of single-cell rna-seq data in r. Bioinformatics. 2017;14 Jan. doi:10.1093/bioinformatics/btw777.
